# Transcriptomics integrated with widely targeted metabolomics reveals the cold resistance mechanism in *Hevea brasiliensis*

**DOI:** 10.3389/fpls.2022.1092411

**Published:** 2023-01-10

**Authors:** Changli Mao, Ling Li, Tian Yang, Mingchun Gui, Xiaoqin Li, Fengliang Zhang, Qi Zhao, Yu Wu

**Affiliations:** The Center of Molecular Biology, Yunnan Institute of Tropical Crops, Xishuangbanna, China

**Keywords:** multi-omics analysis, transcriptomics, metabolomics, cold-resistance, rubber tree

## Abstract

The rubber tree is the primary source of natural rubber and is mainly cultivated in Southeast Asian countries. Low temperature is the major abiotic stress affecting the yield of the rubber tree. Therefore, uncovering the cold resistance mechanism in the rubber tree is necessary. The present study used RNA-sequencing technology and ultra-performance liquid chromatography-tandem mass spectrometry (UPLC-MS/MS) to analyze the transcriptomic and metabolomic changes in two rubber tree clones with different cold resistance capacities (temperature-sensitive Reyan 8-79 and cold-resistant Yunyan 77-4) at 0 h, 2 h, 6 h, and 20 h of exposure to 4°C. Independent analysis of the transcriptome and metabolitome showed that under prolonged low-temperature treatment, Yunyan 77-4 expressed more genes involved in regulating enzyme activity, changing cell permeability, and synthesizing significant metabolites, such as flavonoids and amino acids, than Reyan 8-79. The KEGG annotation and enrichment analysis identified arginine metabolism and biosynthesis of flavonoids as the major pathway associated with cold resistance. Integrated transcriptome and metabolome analysis showed that the increase in the expression of genes modulated flavonoid biosynthesis, arginine biosynthesis, and anthocyanins biosynthesis, resulting in higher levels of metabolites, such as naringenin chalcone, apigenin, dihydroquercetin, cyanidin 3-glucoside, L-arginosuccinate, N-acetyl-ornithine, ornithine, and N-acetyl-glutamate, in Yunyan 77-4 than in Reyan 8-79 after prolonged low-temperature treatment. Phylogenetic analysis identified the genes, such as CHS (*gene356*) and F3H (*gene33147*) of flavonoid biosynthesis and NAGS (*gene16028, gene33765*), ArgC (*gene2487*), and ASS (*gene6161*) of arginine biosynthesis were the key genes involved in the cold resistant of rubber tree. Thus, the present study provides novel insights into how rubber clones resist cold and is a valuable reference for cold-resistance breeding.

## Introduction

The rubber tree (*Hevea brasiliensis* Muell. Arg. 2n = 36), a typical tropical tree from the Amazon Basin, is the primary source of natural rubber. It is mainly grown in the warm, humid rain forests within 0–5°C latitude near the equator and is well adapted to the humid tropics between 10°C south and 10°C north of the equator (Vinod et al., 2010). Natural rubber obtained from *H. brasiliensis* has strong elasticity and toughness and is used to make products such as shoes, tires, and medical equipment (Gronover et al., 2011; Priyadarshan, 2011). During the late 1970s, rubber trees were planted in the Southeast Asian non-traditional rubber planting areas of, such as northeast India, the highlands, and coastal regions of Vietnam and southern China (2009; Raj et al., 2005).

In non-traditional rubber planting areas, abiotic stresses affect the growth and production of rubber. Among the various abiotic stresses, low temperature is a key limiting factor for *Hevea*, a temperature-sensitive plant. Typically, a rubber tree that faces one or more cold advection for more than 20 days in winter suffers cold damage characterized by leaf fall, dead branches, and bark blasting (Wang et al., 2012; Chen et al., 2013a). Subsequently, the tree will undergo a series of physiological and biochemical dysfunctions, such as changes in the cell structure, protoplasm colloidal properties, water status, cell osmotic pressure, photosynthesis, respiration, substance metabolism, and protective enzymes (Lin and Yang, 1994; Mai et al., 2009; Wang, 2010; Wang et al., 2012; Tian et al., 2016). Moreover, research has proven that temperatures below 15°C significantly delay seed germination and decrease the germination rate of rubber (Yan and Cao, 2009). At temperatures below 5°C, rubber tree displays branch and stem exsiccation, which affects plant growth (Mo et al., 2009). Thus, low temperatures reduce rubber yield by 8% to 40%, depending on clones (Roy et al., 2003).

Several abiotic factors, such as low/high temperatures, drought, and salinity, limit plant productivity. These abiotic stresses, especially chilling,affect model transduction, transcriptional processing, translation, and post-translational protein modification, resulting in different metabolites (Rodríguez et al., 2005; Zhang et al., 2022a). Researchers have elucidated the regulatory network of many species under cold stress (Sonna et al., 2002; Shinozaki et al., 2003). Currently, the CBF (C-repeat binding factor) genetic network, known to regulate cold acclimation of *Arabidopsis thaliana*, is one of the well-established pathways associated with cold stress response (Cook et al., 2004). CBFs, the cryogenic signaling transcription factors, interact with the phytochrome-interacting transcription factor PIF3, stabilize the red light and temperature receptor phyB and finally enhance freezing resistance (Jiang et al., 2020). Studies based on the transcriptome, metabolome, and proteome analyses have identified numerous cold-responsive genes from *Momordica charantia* (Niu et al., 2020), *Oryza sativa* (Chen et al., 2003; Maruyama et al., 2014; Pan et al., 2020), *Prunus mume* (Zhuo et al., 2018), and various other species (Rezaie et al., 2020; Bu et al., 2021; Li et al., 2022a; Wang et al., 2022). Moreover, research has found significant enrichment of genes involved in phosphorylation, membrane and protein kinase activity, photosynthesis, photoreception, photoreaction, and beta-trehalose-phosphate synthase in Lanzhou lily (*Lilium davidii*) under cold stress (Tian et al., 2020). Recent studies based on multi-omics revealed the cold resistance mechanism of *Triticum aestivum* (Lu et al., 2020), *Cinnamomum cassia* (Li et al., 2021), *Cryptomeria fortunei* (Zhang et al., 2022b), and various other species (Zhao et al., 2019; Xu et al., 2020), providing a deeper understanding of the genetic response of plants under low-temperature stress.

Most of these earlier studies on low-temperature stress response focused on species with robust tolerance. Only a few studies have been carried out on tropical plants, such as the rubber tree. Recently, a few cold-resistance genes, *HbCBF2* and *HbICEs*, were cloned from the rubber tree, and the expression levels of *HbICEs* were found to be significantly higher in cold-resistant rubber clones than in cold-sensitive ones (Cai et al., 2008; Quan et al., 2017; Li et al., 2022b). With the rapid development of sequencing technology and the completion of whole-genome sequencing, transcriptomics has been used to analyze the cold resistance mechanism of the rubber tree. Transcriptome analysis identified numerous regulators of the cold stress response in rubber tree, including M-type MADS, MYB (v-myb avian myeloblastosis viral oncogene homolog), MYB-related, and NAC (NAM, ATAF1/ATAF2, and CUC2) (Gong et al., 2018). Meanwhile, a comparative analysis of the transcriptome of two clones with different cold resistance capacities revealed that phytohormone signaling, heat shock modules, and reactive oxygen species (ROS) scavengers mediate cold tolerance in the rubber tree (Deng et al., 2018). Studies have also identified the early gene expression profile contributing to cold resistance (Cheng et al., 2018) and the differences in the response strategies among germplasms under low-temperature treatment (Mantello et al., 2019). These reports showed a rapid and intensive response at gene expression levels in the cold-resistance rubber clones than in the cold-sensitive ones. Moreover, coexpression network analysis indicated that the general reaction of rubber trees to short-term cold exposure involves downregulation of gibberellin (GA) signaling, complex regulation of jasmonic acid (JA) signaling, increase in programmed cell death (PCD), and upregulation of ethylene response factor genes (Da et al., 2021). However, most reports on rubber are based on transcriptome analysis, and studies on the metabolite differences are rare. Multi-omics approaches showed differences in gene expression and metabolite levels between tobacco cultivars with different tolerance levels (Jin et al., 2017) and proved the significance of ABA/JA signaling and proline biosynthesis in wheat’s cold tolerance (Zhao et al., 2019). Although the cold resistance genes of rubber trees have been cloned, and the cold resistance mechanism has been analyzed based on the transcriptome, the tolerance mechanism based on a multi-omics approach has not been reported.

Therefore, the present study analyzed the transcriptome and the metabolome of two *Hevea* clones with different cold resistance capacities to characterize the differential genes and metabolites associated with the cold stress response. This study, based on the integration and interpretation of transcriptome and metabolome data sets, will improve our knowledge of the metabolic changes caused by transcriptional regulation after cold stress and reveal the main regulatory genes. We believe the present study’s findings will propose novel candidates for cold resistance breeding in *Hevea*.

## Materials and methods

### Plant materials and low-temperature treatment

The bud-grafted plants of the rubber tree clones, namely Yunyan 77-4 (Triploid, cold-resistant clone) (Li, 2005; Li et al., 2009) and Reyan 8-79 (cold-sensitive clone), were used in this study. The seedlings were cultivated in the rubber tree breeding nursery of Yunnan Institute of Tropical Crops, Yunnan, China, and the one-year-old plants were subjected to low-temperature treatment in an artificial box precooled from room temperature to 28°C and 4°C at a cooling rate of 4°C/h. The plants were exposed to continuous low-temperature treatment (4°C) for 2, 6, and 20 h under a 12 h/12 h dark/light cycle, 10,000 lx light intensity, and 75% relative humidity; plants grown at room temperature were used as the control (0 h). We collected twenty-four treatment samples (leaves), including Y0H, Y2H, Y6H, and Y20H from Yunyan 77-4 and R0H, R2H, R6H, and R20H from Reyan 8-79 at 0, 2, 6, and 20 h after exposure to cold stress, maintaining three biological replicates per treatment; Y0H and R0H were used as the control samples (28°C, 0 h). Nine seedlings per clone were treated, of which three formed a replicate. At each treatment time point, the middle leaflet was collected from the second strata, the veins were removed, and the leaves were cut into 1 cm × 1 cm pieces; these pieces were mixed and divided into three parts for RNA-Seq, metabolite analysis, and quantitative real-time polymerase chain reaction (qRT-PCR). The leaf samples were frozen in liquid nitrogen immediately after collection and stored in an ultra-low temperature (-80°C) freezer.

### Transcriptome analysis

#### RNA extraction, quantification, and sequencing

Total RNA was extracted from the 24 samples using TRIzol reagent (Invitrogen, Carlsbad, USA) according to the manufacturer’s instructions, followed by poly(A) mRNA enrichment with the oligo (dT) magnetic beads and fragmentation with the fragmentation buffer. The first-strand cDNA was synthesized from this fragmented mRNA (template) using random hexamers, followed by second-strand synthesis using dNTPs, RNase H, and DNA polymerase I. The short, double-stranded cDNA was purified with AMPure XP beads and subjected to end repair; then, an A-tail was added, and a sequencing adaptor was connected, followed by fragment size selection with AMPure XP beads. Finally, the cDNA libraries were obtained by PCR enrichment. Before sequencing, the quality of the cDNA library was assessed using Agilent 2100 Bioanalyzer (Agilent Technologies, Inc. Waldbronn, Germany), and the concentration was measured using a Qubit 2.0 fluorometer (Life Technologies, Carlsbad, CA, USA). Transcriptome sequencing was performed on an Illumina NovaSeq6000 platform (Illumina, San Diego, CA, United States). The Beijing Biomarker Biotechnology Co., Ltd (Beijing, China) performed RNA sequencing and transcriptome assembly for the 24 libraries (eight leaf samples with three replicates each).

#### Data analysis

The adaptor sequences, ambiguous reads with unknown nucleotides > 5%, or low-quality sequences withQ20 < 20% (percentage of sequences with sequencing error rates < 1%) were removed from the RNA-seq raw data by a perl script. The high-quality reads were mapped to the rubber tree (*Hevea brasiliensis*) genome (the assembly number: ASM1045892v1, https://www.ncbi.nlm.nih.gov/data-hub/genome/GCA_010458925.1/) using Tophat2.0.8 software under the below parameter: “read-realign-edit-dist” was set to “0”, and the remaining parameters used the default values (Kim et al., 2013). Fragments per kilobase of transcript per million mapped fragments (FPKM) were used as an index to measure the gene expression levels based on the RNA-seq data. The differentially expressed genes (DEGs) between the two rubber tree clones at the three stages of cold treatment were analyzed using the DESeq2 (an online analysis tool in the OmicShare cloud platform, https://www.omicshare.com/tools/Home/Soft/diffanalysis); genes with a fold change ≥ 2 and a false discovery rate (FDR) < 0.01 were defined as differentially expressed. Here, the FDR was obtained by correcting the differential significance p-value based on the multiple hypothesis test and the Benjamini-Hochberg method. Further, gene clustering, KEGG (Kyoto Encyclopedia of Genes and Genomes) and KO (KEGG Orthology) (Kanehisa et al., 2016) functional annotation, and enrichment analysis were performed for these DEGs using the OmicShare cloud platform (https://www.omicshare.com/tools/).

#### Quantitative real-time polymerase chain reaction

Eight DEGs were selected for qRT-PCR validation of the RNA-seq data using three technical replicates per sample. The qRT-PCR was performed on a LightCycler480 fluorescence quantitative PCR instrument (Rotkreuz, Switzerland) using the rubber actin gene (Primer sequence5’-3’: Forward- CAAGGGTGAATACGATGAGTCTG, Reverse-GCCTCTCACTAGCAGCCATAAC) as the internal reference. The qRT-PCR primers were designed with the Primer Premier5.0 software (**Supplementary Table 1**), and the relative gene expression levels were calculated following the 2^−ΔΔCT^ method (Livak and Schmittgen, 2001).

### Metabolomic analysis

#### Sample preparation and metabolite extraction

The leaf samples were freeze-dried in a lyophilizer (Scientz-100, Ningbo Scientz Biotechnology Co. Ltd., Ningbo, Zhejiang, China) and crushed in a blender (MM 400, Retsch) at 30 Hz for 1.5 min. Approximately 100 mg of the powder was dissolved in 1.2 mL of 70% methanol, vortexed every 30 min for 30 s, six times, and stored overnight in a refrigerator (4°C). The following day, the extract was centrifuged at 12,000 rpm for 10 min, and the supernatant was filtered through a microporous filter membrane (0.22 μm) into a sample vial. The metabolites in each sample extract were then analyzed using ultra-performance liquid chromatography-tandem mass spectrometry (UPLC-MS/MS).

#### Metabolite detection and qualitative and quantitative analyses

Widely targeted metabolomic analysis based on UPLC-MS/MS was performed at the Metware Biotechnology Co., Ltd. (Wuhan, Hubei, China). An AB Sciex high-resolution according to their standard procedure as follow: the widely targeted metabolomics uses high-resolution mass spectrometry AB sciex TripleTOF660 for qualitative detection of mixed samples, and then uses AB sciex4500 QTRAP for quantification, the operation parameters were as follows: ion source, turbo spray; source temperature 550°C; ion spray voltage (IS) 5500 V (positive ion mode)/-4500 V (negative ion mode); ion source gas I (GSI), gas II (GSII), curtain gas (CUR) were set at 50, 60, and 25.0 psi, respectively; the collision-activated dissociation (CAD) was high. Using multiple reaction monitoring (MRM), high-resolution mass spectrometry for accurate qualitative, triple quadrupole mass spectrometry with high sensitivity, high specificity and excellent quantitation capabilities as a complementary tool. Identification was conducted by match of mass spectrum to reference library MetWare database (MWDB) (Chen et al., 2013b) based on the accurate mass, secondary (MS2) fragments, isotope distribution, and retention time (RT). Here, both of MS tolerance and MS2 tolerance were set to 20 ppm, and the RT offset to a maximum of 0.2 minutes. A quality control (QC) sample was analyzed after every tenth test sample to ensure the reproducibility of the analysis.

#### Metabolite data analysis

The orthogonal partial least squares discriminant analysis (OPLS-DA) and principal component analysis (PCA) were carried out on all the samples to identify the putative biomarkers after data normalization. Finally, the metabolites with variable importance in projection (VIP) ≥ 1 and fold change ≥ 2 or ≤ 0.5 were defined as the differentially accumulated metabolites (DAMs).

### Integrated analysis of metabolomic and transcriptomic data

The transcriptome and metabolome were normalized to investigate the relationship between the changes in the genes and the metabolites of rubber tree clones with different cold resistance capacities. Further, a PCA was performed on the transcriptome and metabolome to visualize the differences in the transcriptome and metabolome between the sample groups. Then, all differential genes and metabolites were simultaneously mapped to the KEGG database to identify the significant pathways associated with both DEGs and DAMs. Pearson correlation coefficients (PCC) were calculated for the DEGs and DAMs of each group, and the metabolites and genes with 0.8 ≤ PCC ≤ -0.8 were considered significantly correlated. Then, the PCC values were ranked to screen the strongly correlated genes and metabolites under different treatments and confirm the key regulatory genes and metabolites of the rubber tree clones responsible for low-temperature resistance. PCA and KEGG pathway enrichment were conducted on the Omicshare analysis cloud platform (https://www.omicshare.com/tools/). The phylogenetic analysis was performed following the maximum likelihood (ML) method in MEGA 7.0 (Felsenstein, 1978).

## Results

### Transcriptome analysis of rubber tree clones with different cold resistance capacities

**Figure 1:**
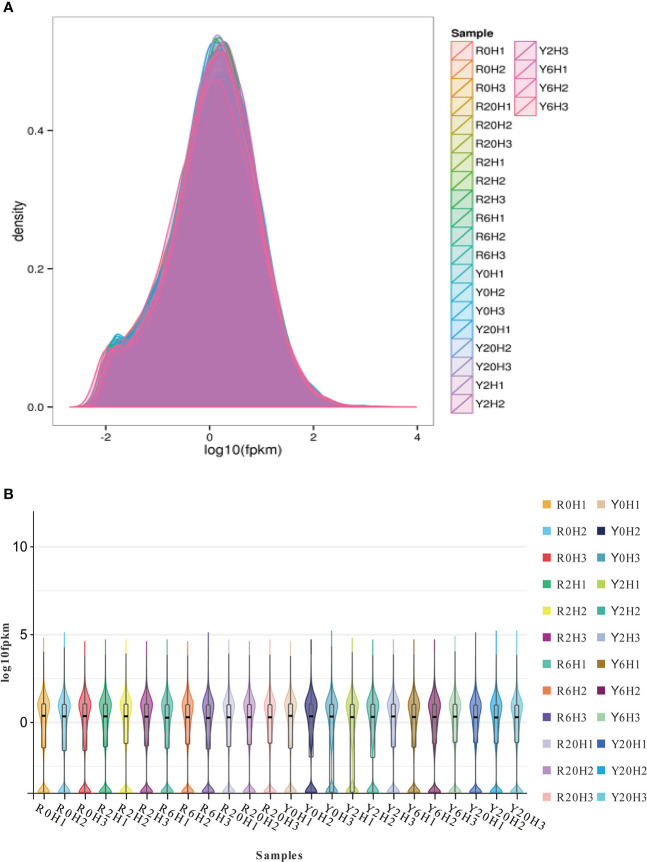
The gene expression density diagram and the violin plot for the transcripts of the rubber tree samples. **(A)** Gene expression density diagram. Comparison of FPKM density distribution. The curves in different colors represent different samples; the abscissa indicates the log value of the FPKM and the ordinate indicates the probability density. **(B)** Violin plot. The upper and lower ends of the lines represent the maximum and minimum of non-outliers; the upper and lower edges of the rectangles represent the third and first quartiles of the data; the center dot represents the median of the data.

Approximately 41.24−64.27 M 100 bp pair-end, high-quality sequences were obtained from the raw RNA-seq data after quality control (QC) (**Supplementary Table 2**). About 73.33% to 89.22% of the clean reads aligned to unique locations on the rubber tree reference genome and 5.31% to 13.2% to multiple locations (**Supplementary Table 2**). The gene expression density diagram for the rubber samples at different durations after cold exposure indicated similar trends in gene abundance and gene expression density. Moreover, the log FPKM values were concentrated in the [-2, 2] interval for all transcripts of the samples (**Figure 1**). The correlation heatmap (**Supplementary Figure 1A**) and PCA plot (**Supplementary Figure 1B**) showed similarities among replicates of the same sample and apparent differences among the different cold-resistance rubber tree clones, indicating the reliability of the data.

### DEGs identification and verification

**Figure 2:**
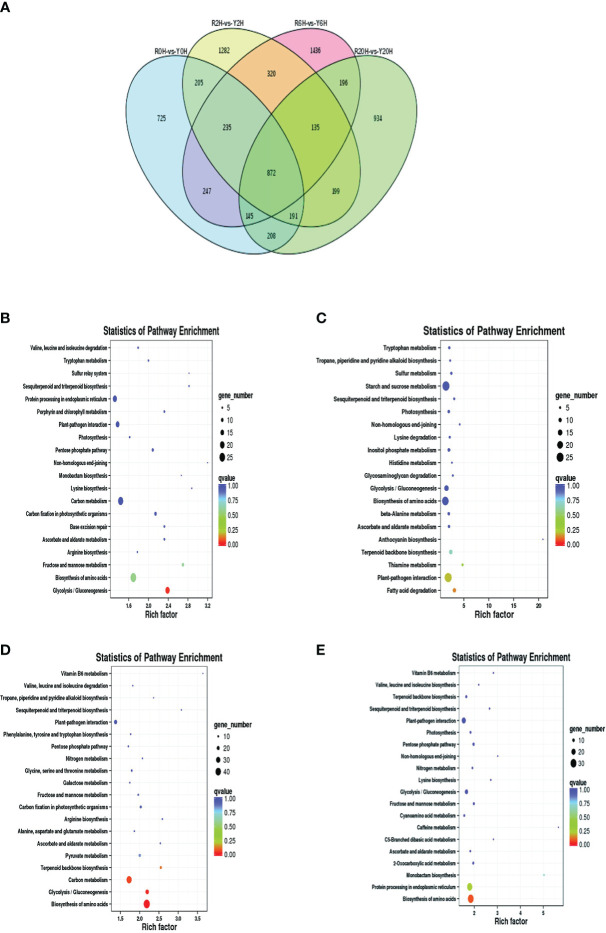
Statistics and KEGG enrichment analysis of DEGs. **(A)** Venn diagram of DEGs between Reyan 8-79 and Yunyan 77-4. KEGG enrichment of DEGs of **(B)** R0H_vs_Y0H, **(C)** R2H_vs_Y2H, **(D)** R6H_vs_Y6H, and **(E)** R20H_vs_Y20H comparisons. The horizontal coordinate indicates the Rich factor of each pathway (Rich factor was calculated as the ratio of the number of differentially expressed genes annotated in a pathway to the number of all genes annotated in this pathway), and the vertical coordinate is the pathway’s name. The dot color represents the *p*-value; the redder it is, the more significant the enrichment. The size of the dots represents the number of differential metabolites enriched in the pathway.

A total of 7330 DEGs were identified between Reyan 8-79 and Yunyan 77-4 after 2 h, 6 h, and 20 h of exposure to cold temperature (**Figure 2A**); 872 DEGs were common to both clones and three treatment durations. The numbers of DEGs of the different groups are listed in **Table 1**. Detailed examination of the data revealed that for the Reyan 8-79 vs. Yunyan 77-4 comparisons, the downregulated genes were more than the upregulated genes at 0 h and 2 h, while the upregulated genes were more at 6 h and 20 h.

**Table 1:** Summary of the differentially expressed genes and differentially accumulated metabolites.

Group	DEGs	Upregulated DEGs	Downregulated DEGs	DAMs	Upregulated DAMs	Downregulated DAMs
R0H_vs_Y0H	2828	1440	1388	180	155	25
R2H_vs_Y2H	3439	1490	1949	127	97	30
R6H_vs_Y6H	3586	2040	1546	166	113	53
R20H_vs_Y20H	2880	1627	1253	213	176	37

Subsequent gene ontology (GO) analysis indicated that the terms “catalytic activity” and “binding” were prominent in the “molecular function” ontology, “cell” and “cell part” in the “cellular component” ontology, and “metabolic process” and “cellular process” in the “biological process” ontology (**Supplementary Figures 2A–D**). The KEGG pathway enriched by the DEGs were “Plant-pathogen interaction”, “Starch and sucrose metabolism”, “Carbon metabolism”, “Plant hormone signal transduction”, and “Biosynthesis of amino acid” (**Supplementary Figures 2E–H**). The “Glycolysis/Gluconeogenesis” pathway was enriched at 0 h, whereas the “Biosynthesis of amino acids”, “Carbon metabolism”, “Glycolysis/Gluconeogenesis”, and “Terpenoid backbone biosynthesis” pathways were enriched at 6 h. Meanwhile, only the “ Biosynthesis of amino acids” pathway was significantly enriched at 20 h (**Figures 2B–E**).

### Metabolites of the rubber tree clones with different cold resistance capacities

**Figure 3:**
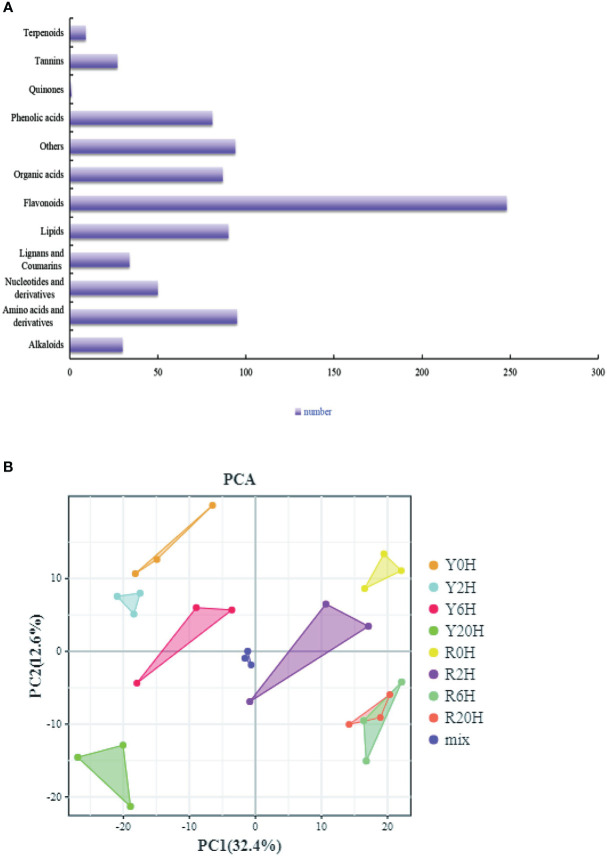
Overall analysis of the metabolites. **(A)** All identified metabolites. **(B)** PCA plot of all samples, including the QC sample.

Furthermore, we performed a UPLC-MS/MS-based widely targeted metabolome analysis to identify the DAMs of the rubber tree clones under low-temperature treatment. The metabolites were qualitatively and quantitatively analyzed using MWDB and MRM, respectively. The total ions current (TIC) of the QC sample and the multimodal detection map in MRM indicated similar RTs and peak intensities between samples, while the MS occurrence varied among the time points. Analysis of the TIC plots of the MS detection test and the various QC samples showed high overlap in the TIC metabolite detection curve (**Supplementary Figures 3A, B**). All the substances detected in the samples are shown on the multinodal map. The present study identified 846 metabolites from the samples, including a large proportion of “Flavonoids”, “Amino acids and derivatives”, “Others”, and “Lipids” (**Figure 3A**). PCA for all samples, including the QC sample, showed large differences between groups and less variability within groups (**Figure 3B**). Meanwhile, the OPLS-DA showed large differences between the two clones under the same treatment period. The validation of the OPLS-DA model showed that the p-value was less than 0.005 in all groups (R0H vs. Y0H, R2H vs. Y2H, R6H vs. Y6H, and R20H vs. Y20H), indicating the reliability of the OPLS-DA model is reliable (**Supplementary Figures 3C–F**).

### DAM identification and verification

Further, to assess the metabolic differences and group the samples, the OPLS-DA was performed. In the OPLS-DA, Q2 > 0.9 indicates an excellent predictive ability (Chung et al., 2019). The permutation tests for the OPLS-DA model yielded Q^2^ and R^2^X ranging from 0.818−0.936 and 0.659–0.691, respectively, with a p-value < 0.05 (**Supplementary Figures 3C–F**), indicating that the model fitted well and have good predictive ability, and the groups were separated well. The study identified 180 (155 upregulated and 25 downregulated), 127 (97 and 30), 166 (113 and 53), and 213 (176 and 37) DAMs from the R0H vs. Y0H, R2H vs.Y2H, R6H vs. Y6H, and R20H vs. Y20H pairwise comparisons, respectively (**Table 1**), based on the OPLS-DA results and the screening criteria (VIP ≥ 1; fold change ≥ 2 and ≤ 0.5) (**Supplementary Table 3**). Of these, 180 metabolites were common to all groups. The DAMs showed obvious differences between Yunyan 77-4 and Reyan 8-79. Interestingly, the DAMs associated with cold resistance in other species, such as glucose and putrescine, were not accumulated in the rubber tree; however, flavonoids, lipids, and amino acids exhibited huge differences between the rubber tree clones with different cold resistance capacities. Moreover, the content of flavonoids in the cold-resistant clone Yunyan 77-4 was higher than that in the cold-sensitive clone Reyan 8-79 at room temperature and 4°C; the content of amino acids in Yunyan 77-4 was also higher than that in Reyan 8-79 at room temperature and after 4°C exposure for 2 h. Meanwhile, after 6 h of exposure to 4°C, the content of amino acids in Yunyan 77-4 was lower than that in Reyan 8-79, and after 20 h, the amino acid content was almost the same in both the clones. The lipids were higher in Yunyan 77-4 than in Reyan 8-79 at room temperature, but higher in Reyan 8-79 than in Yunyan 77-4 at 4°C after 2 h and 6 h of exposure. However, the difference between these clones was not obvious at room temperature after 20 h. Heatmap analysis of the DAMs showed that the flavonoid terms “Dihydroflavonol” and “Biflavones” in Yunyan 77-4 vs. Reyan 8-79 groups increased with prolonged exposure to low temperature (**Supplementary Figure 4**, **Supplementary Table 4**). Further KEGG analysis showed that the DAMs enriched the “Metabolic pathway”, “Flavonoid biosynthesis”, “Biosynthesis of secondary metabolites”, “Arginine and proline metabolism”, and “Anthocyanin biosynthesis” classes (**Supplementary Figure 5A**). Among these, the DAMs in Yunyan 77-4 vs. Reyan 8-79 at all treatment duration enriched the “Anthocyanin biosynthesis” pathway; the DAMs of R0H vs. Y0H and R2H vs. Y2H enriched the “Flavone and flavonol biosynthesis”, and the DAMs of R20H vs. Y20H enriched the “Isoflavonoid biosynthesis”. Meanwhile, the “Arginine and proline metabolism” pathway was the most important pathway in all groups (**Supplementary Figure 5B**). We also performed the enrichment analysis for the upregulated and downregulated DAMs separately. The downregulated DAMs enriched the “Phenylpropanoid biosynthesis” at 0 h and 2 h of exposure to low temperature, the “Tropane, piperidine, and pyridine alkaloid biosynthesis”; “Biosynthesis of various plant secondary metabolites”; “Arginine biosynthesis”; “Phenylalanine, tyrosine, and tryptophan biosynthesis”; “Phenylpropanoid biosynthesis” at 6 h, and the “Purine metabolism” at 20 h. Meanwhile, the upregulated DAMs enriched the “Biosynthesis of flavonoid” term at all treatment durations (**Supplementary Figure 6**).

### Integrated analysis of the transcriptomic and metabolomic data reveals the cold resistance mechanism of rubber tree

We then integrated the transcriptome and metabolome data sets to understand the mechanisms underlying cold resistance in the rubber tree. The PCA of transcriptome and metabolome showed that Y20H accounted for the first principal component, and the two clones at room temperature (0 h) accounted for most of the second principal component. Then, the DEGs and DAMs were simultaneously mapped to the KEGG pathway map to assess the relationship between genes and metabolites better. The KEGG enrichment and pathway analysis showed that the DAMs and DEGs enriched “Flavonoid biosynthesis”, “Biosynthesis of amino acids”, and “Arginine and proline metabolism” pathways at 0 h; “Anthocyanin biosynthesis” and “Arginine and proline metabolism” were significantly enriched at 2 h. Interestingly, “Flavonoid biosynthesis” was significantly enriched in addition to “Amino acid biosynthesis” at 6 h and 20 h of exposure to 4°C (**Supplementary Figure 7A**). Further, the heatmap based on the PCC between DEGs and DAMs (0.8 ≤ PCC ≤ -0.8) showed that flavonoids were the highest at all time points, followed by phenolic acids, organic acids, tannins, and amino acids and derivatives (**Supplementary Figure 7B**). Thus, the integrated analysis identified flavonoids, arginine, and anthocyanins as the key metabolites regulating cold resistance in the rubber tree.

### Identification of the cold resistance genes associated with the biosynthesis of flavonoids, arginine, and anthocyanins

**Figure 4:**
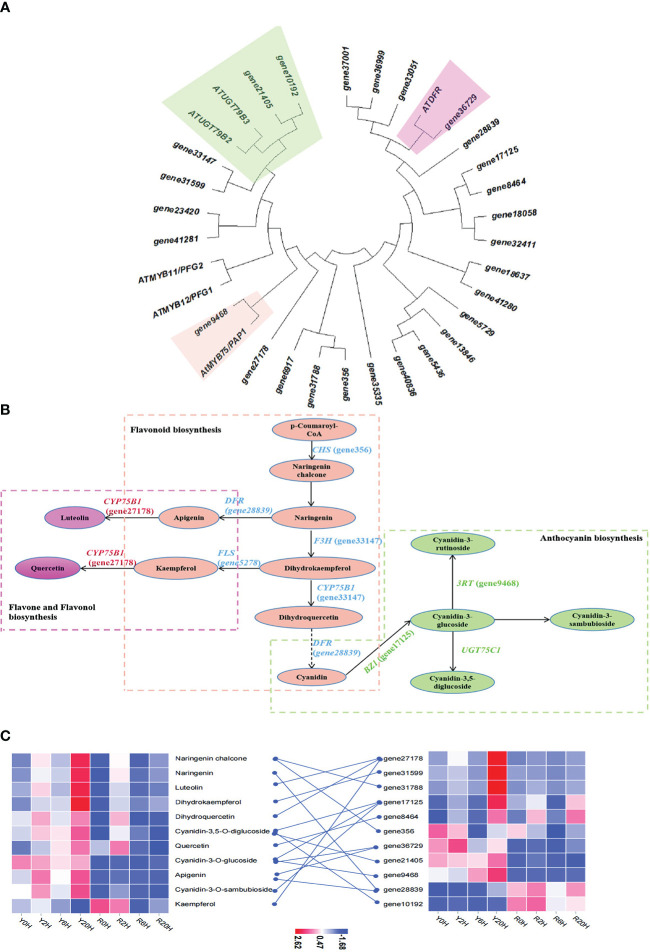
Metabolites and genes involved in flavonoid biosynthesis and anthocyanin biosynthesis. **(A)** Maximum likelihood phylogenetic tree of the key genes of the flavonoid and anthocyanin biosynthetic pathways. The colored blocks highlight the relationship between genes involved in flavonoid, anthocyanin, and flavone biosyntheses identified in this study and the genes of the same pathway in *Arabidopsis thaliana*. **(B)** Flavonoid biosynthetic mechanisms associated with cold resistance in the rubber tree. The green dashed box represents anthocyanin biosynthesis, the orange dashed box represents flavonoid biosynthesis, and the pink dashed box represents flavone and flavonol biosynthesis. Ellipses in the same color represent the same pathway, and the names in parentheses beginning with the term “gene” are the IDs of the genes annotated in this study. **(C)** The heatmap of metabolites and genes involved in flavonoid biosynthesis, anthocyanin biosynthesis, and flavone biosynthesis based on the FPKM of key genes and the content of metabolites.

The enrichment analysis integrating DEGs and DAMs revealed flavonoid biosynthesis, arginine biosynthesis, and anthocyanin biosynthesis as the predominant metabolic pathways related to cold resistance in rubber trees. Then, to identify the key genes regulating these pathways under low temperatures, a phylogenetic analysis was performed between the genes that demonstrated a good correlation with metabolites of the flavonoid and anthocyanin biosynthetic pathway in this study and similar to the cold resistance genes of the same pathway in *Arabidopsis thaliana* (Li et al., 2017). The ML phylogenetic tree showed the highest similarity for *gene10192*, *gene21405*, *gene9468*, and *gene36729* of the rubber tree with the cold-resistance genes *ATUGT79B3*, *ATUGT79B2*, *AtMYB75*/*PAP1*, and *ATDFR* of *A. thaliana* (**Figure 4A**). Further KEGG annotation and enrichment analysis confirmed that *gene27178*, *gene7125*, *gene28839*, *gene31599*, *gene356*, and *gene05278* participated in the biosynthesis and metabolism of rubber flavonoids. Integrated analysis of the DEGs and DAMs with 0.8 ≤ PCC ≤ -0.8 data and related to flavonoid biosynthesis and metabolism during cold resistance in the rubber tree (**Figure 4B**) revealed that flavonoids, anthocyanins, and flavones were biosynthesized from *p*-coumaroyl-CoA through the action of chalcone synthase (CHS) under cold stress in the resistant clone. Generally, under low temperatures, among the intermediates of the flavonoid pathway, apigenin and kaempferol are involved in flavone and flavonol biosynthesis, while cyanidin is involved in anthocyanin biosynthesis. The content of kaempferol and cyanidin was lower in Yunyan 77-4 than in Reyan 8-79, while that of apigenin was higher at room temperature or low temperature. Additionally, heatmap clustering of the genes (FPKM values) and metabolites (content) involved in the cryogenic regulation of flavonoid biosynthesis and anthocyanin biosynthesis showed differences in the expression of flavonoid biosynthetic genes and the content of flavonoids between the two rubber tree clones (**Figure 4C**). Specifically, Yunyan 77-4 exhibited the highest content of flavonoids and expression of flavonoid-related genes under prolonged low-temperature treatment; however, the trend in this relationship between the genes and metabolites was not found in Reyan 8-79 (**Figure 4C**).

**Figure 5:**
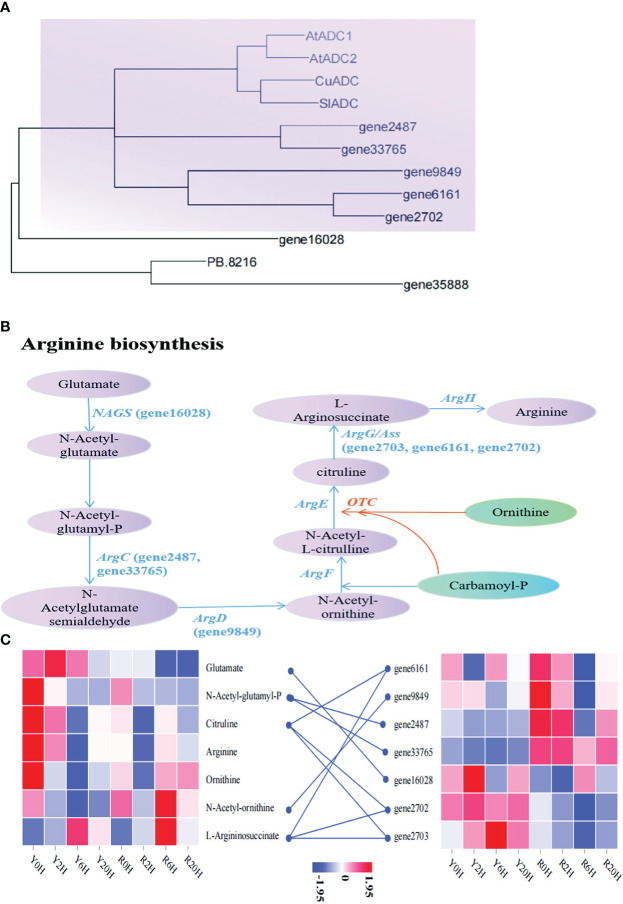
Metabolites and genes involved in arginine biosynthesis **(A)** Maximum likelihood phylogenetic tree of the key genes in arginine biosynthetic pathway. The colored blocks highlight the relationship between genes involved in arginine biosynthesis identified in this study and the genes of the same pathway in *Arabidopsis thaliana*, *Cucumis sativus*, and *Solanum lycopersicum*. **(B)** Arginine biosynthetic mechanisms associated with cold resistance in the rubber tree. The name in parentheses beginning with the term “gene” are the IDs of the genes annotated in this study. **(C)** The heatmap of metabolites and genes involved in arginine biosynthesis based on the FPKM of key genes and the content of metabolites.

Similarly, to search for key genes involved in arginine biosynthesis under low temperatures, we performed another phylogenetic analysis using the arginine biosynthesis related-genes annotated in this study and the arginine decarboxylase (ADC) genes reported in *A. thaliana* (*AtADC1*: *AT2G16500*; *AtADC2*: *AT4G34710*), *Cucumis sativus* (*CuADC*, Sequence ID: NM_001308900.2), and *Solanum lycopersicum* (SlADC, Sequence ID: NM_001247720.2). The ML tree showed that *gene2487, gene33765*, *gene9849*, *gene6161*, and *gene2702* were highly similar to the reported ADC genes of *A. thaliana*, *C. sativus*, and *S. lycopersicum* (**Figure 5A**). The KEGG annotation and enrichment analysis showed that these genes belonged to the members of the Arg gene family, confirming their role in arginine biosynthesis. The pathway in the rubber tree elucidated based on these observation is demonstrated in **Figure 5B**. In addition to the genes mentioned above, *gene16028*, *gene35888*, and *PB.8216* also participated in arginine biosynthesis in the rubber tree. Additionally, the heatmap showed a higher arginine content in Yunyan 77-4 than in Reyan 8-79 before cold exposure; however, almost an opposite trend was observed in *gene2487* and *gene33765*. These genes displayed a higher expression level in Reyan 8-79 but did not increase the content of the associated metabolites of arginine biosynthesis, such as N-acetyl-glutamyl-P (**Figure 5C**), resulting in a weak cold resistance of Reyan 8-79. Thus, our observation suggested that the differences in the expression patterns of genes involved in the biosynthesis of flavonoids and arginine led to differences in the content of flavonoids, arginine, and their intermediates, resulting in different cold resistance capacities between the clones.

### Quantitative real-time polymerase chain reaction

**Figure 6:**
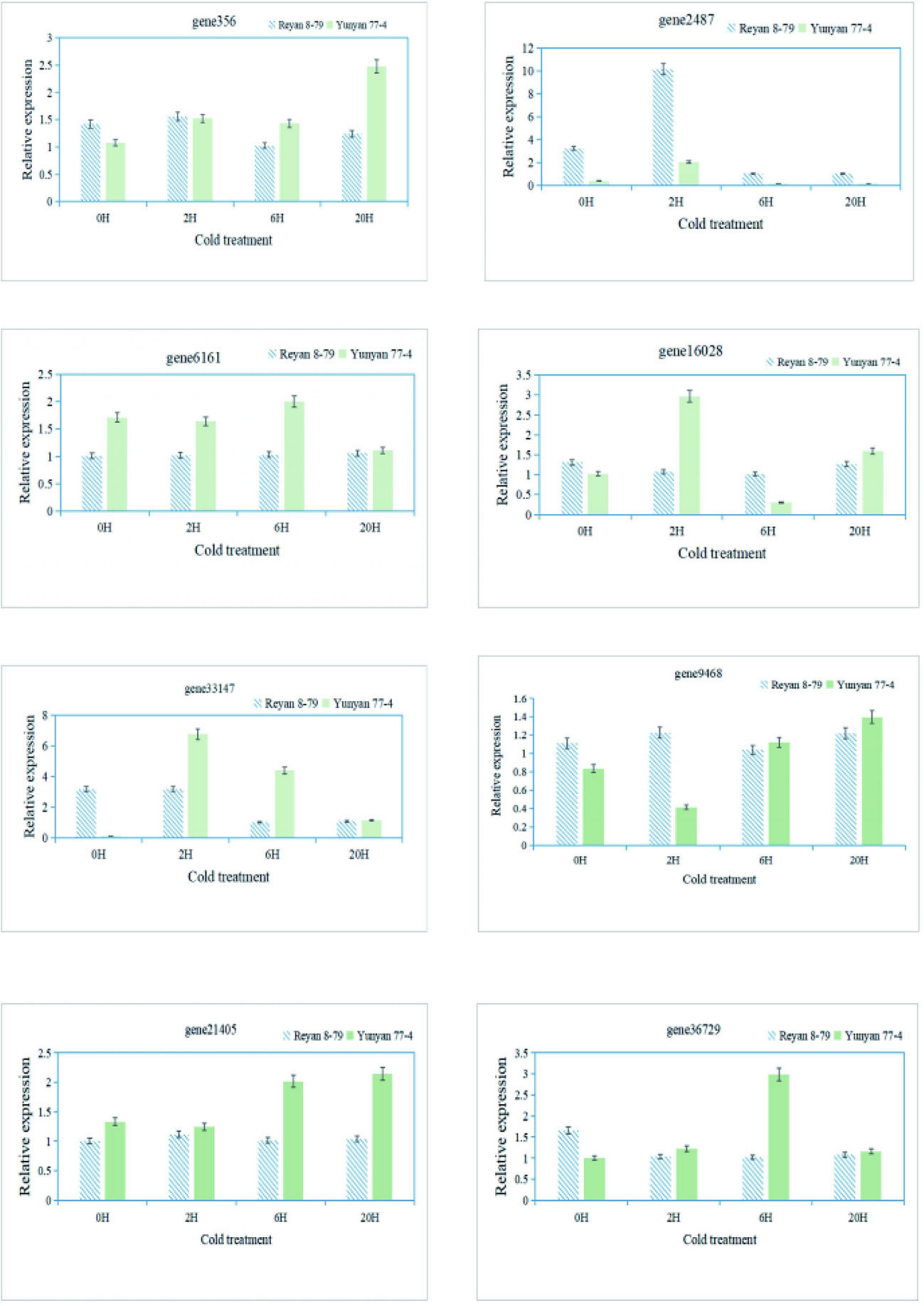
qRT-PCR validation of the transcript levels of DEGs of the arginine and flavonoid biosynthetic pathway.

Finally, qRT-PCR was performed to validate the RNA-Seq data and confirm the expression levels of eight genes associated with the arginine and flavonoid biosynthesis pathway in different samples (**Supplementary Table 4**). The expression patterns of these genes obtained by qRT-PCR showed good consistency with the RNA-Seq results, indicating the reliability of the transcriptome data (**Figure 6**).

## Discussion

In the present study, the cold-resistance clone Yunyan77-4 and the cold-sensitive clone Reyan 8-79 were exposed to 4°C for different durations, and the response was analyzed. The DEGs identified between the two rubber tree clones based on transcriptome sequencing mainly enriched the glycolysis/gluconeogenesis pathway at room temperature and the biosynthesis of amino acids at 6 h under low temperature. These observations indicated that Yunyan 77-4 expressed more genes under prolonged low-temperature treatment than Reyan 8-79. Meanwhile, KEGG analysis suggested that the rubber tree responds to cold stress by regulating enzyme activity, changing cell permeability, and synthesizing significant metabolites. Subsequent metabolome analysis indicated that under prolonged treatment, the DAMs increased, and Yunyan 77-4 had higher metabolite content than Reyan 8-79. Arginine biosynthesis and flavonoid and anthocyanin biosyntheses were significantly enriched at 0 h and 2 h in Yunyan 77-4; however, enrichment of arginine biosynthesis reduced at 6 h and 20 h, while the biosynthesis of flavonoids and anthocyanins remained significantly enriched. In addition, 97 (93 upregulated and 3 downregulated), 63 (57 and 5), 74 (68 and 5), and 123 (117 and 5) flavonoids were found differentially accumulated in Reyan 8-79 compared with Yunyan 77-4 at 0 h, 2 h, 6 h, and 20 h, respectively. The cold-resistant clone Yunyan 77-4 had a higher content of flavonoids than the cold-sensitive Reyan 8-79. As the treatment prolonged, the species and content of flavonoids in the cold-resistant clone increased and attained more elevated levels than in the temperature-sensitive clone. These observations indicate that the cold-resistant rubber tree shows more robust gene expression than the temperature-sensitive one for adjusting enzyme activity, changing cell permeability, and synthesizing metabolites.

Integrated transcriptome and metabolome analysis revealed significant enrichment of the arginine and proline metabolism pathway at 2 h; however, the enrichment was reduced with prolonged treatment (6 h and 20 h), while flavonoid biosynthesis was enriched. The primary reason for this change in Yunyan 77-4 is the upregulation in the genes encoding CHS, flavanone 3-hydroxylase (F3H), and FDR involved in flavonoid biosynthesis, and the high content of flavonoids, including naringenin, chalcone, and dihydrokaempferol. Moreover, Yunyan 77-4 exhibited higher expression levels of genes involved in flavonoid biosynthesis and accumulation of flavonoids and anthocyanins than Reyan 8-79 after low-temperature exposure for 6 h and 20 h. The arginine biosynthetic genes encoding N-acetylglutamate synthase (NAGS, *gene16028* in this study), N-acetyl-gamma-glutamyl-phosphate reductase (ArgC, *gene2487*, *gene33765*), and argininosuccinate synthase (Ass, *gene2703*, *gene6161*, *gene2702*), and the metabolites L-arginosuccinate, N-acetyl-ornithine, ornithine, and N-acetyl-glutamate were significantly different between the two rubber tree clones under low temperature. KEGG analysis indicated arginine biosynthesis was significantly enriched only at 6 h in Yunyan 77-4. The NAGS gene and N-acetyl-glutamate regulated by this gene were upregulated at 2 h, the ArgC gene and N-acetyl-ornithine content were downregulated at 6 h and 20 h, and the ASS gene was upregulated at all time points. Moreover, arginine content was consistently higher in Yunyan 77-4 than in Reyan 8-79. Arginine, the primary form in which organic nitrogen is stored and transported in plants, is a key precursor of polyamine biosynthesis and plays an important role in growth, development, and stress regulation (Winter et al., 2015). Overexpression of NAGS in *A. thaliana* increased salt and drought tolerance (Mary et al., 2009). Thus, the present study’s results indicate that the resistant clone synthesizes arginine and its precursors under the action of Arg genes, providing better cold tolerance. In addition, the two clones showed differences in glutamate and related genes and ornithine. Therefore, we speculated that the treatment duration obviously influenced arginine biosynthesis, and NAGS, ArgC, and Ass genes were necessary for low-temperate response in the rubber tree.

In plants, flavonoids participate in many processes associated with biotic and abiotic stresses (Samanta et al., 2011). Flavonoid content and type vary based on the development phase and tissues (Makoi and Ndakidemi, 2011; Choi et al., 2012). In this study, the continuous low-temperature treatment upregulated the expression of flavonoid biosynthetic genes and the accumulation of flavonoids and anthocyanins in the rubber tree, consistent with the reports in other species, such as grape and *A. thaliana* (Azuma et al., 2012; Nakabayashi et al., 2014). After cold treatment for 6 h, the CHS and F3H genes were upregulated in Yunyan 77-4. These two genes probably promoted the accumulation of flavonoids, such as apigenin and dihydroquercetin (Dao et al., 2011), the key substrates for anthocyanin, flavone, and flavonol biosynthesis. The correlation observed between the increased content and CHS and F3H upregulation confirmed this hypothesis. Similarly, low temperatures upregulated the CHS gene in barley (*Hordeum vulgare*) and provided stress tolerance (Lee et al., 2019). Moreover, other genes of this pathway (CYP75B1: *gene27178*; FDR: *gene28839*; 3RT: *gene9468*) (**Figure 4C**) were upregulated in the cold-resistant clone, and the content of the related metabolites increased accordingly. Thus, the observations indicate that the accumulation of flavonoids in the cold-resistant rubber tree also contributed to their enhanced cold resistance.

## Conclusion

The study found that the variations in the level of flavonoids, glutamate, and ornithine led to the difference in the cold resistance capacity between Yunyan 77-4 and Reyan 8-79. The content of flavonoids, especially the intermediates of anthocyanin biosynthetic pathway, was higher in Yunyan 77-4 than in Reyan 8-79 at room temperature and low temperature. During the early stage of cold stress, more glutamates were synthesized in the cold-resistant rubber tree clone. With prolonged exposure, compounds of the arginine biosynthetic pathway, such as L-arginosuccinate, N-acetyl-ornithine, N-acetyl-glutamate, and ornithine, were accumulated more in Yunyan 77-4 than in Reyan 8-79. The study also found differences in the content of naringenin chalcone, apigenin, dihydroquercetin, and cyanidin-3-glucoside of flavonoid biosynthetic pathway between the two rubber tree clones, with variations in the expression of the genes regulating their biosyntheses. Thus, the study suggests that flavonoid biosynthetic genes, such as CHS (*gene356*) and F3H (*gene33147*), and the arginine biosynthetic genes, including NAGS (*gene16028*), ArgC (*gene2487*, *gene33765*), and Ass (*gene6161*), play key roles in regulating cold resistance of the rubber tree. However, the role of transcription factors, such as CBF1 and MYB, in regulating these genes remains unclear. Thus, the study provides an empirical basis for future investigations on the molecular and biochemical mechanisms underlying cold resistance in the rubber tree and the genes identified in present study may be used as candidate to develop improved new cold-resistant varieties.

## Data availability statement

The original contributions presented in the study are publicly available. This data can be found here: https://dataview.ncbi.nlm.nih.gov/object/PRJNA855273?reviewer=fam1jekvgc6ts689hp1ihtrt1c, PRJNA855273.

## Author contributions

CM: writing - original draft, proofreading, metabolomic test, data curation, analysis, and visualization. LL: material preparation and transcriptomic data analysis. TY and XL: metabolome data analysis. MG: experimental seedling preparation. FZ and QZ: manuscript proofreading. YW: supervision, project administration, and funding acquisition. All authors contributed to the article and approved the submitted version.
