# Supplementary material for: Transcriptomics integrated with widely targeted metabolomics reveals the cold resistance mechanism in Hevea brasiliensis
Source: Front Plant Sci. 2023 Jan 10;13:1092411. doi: 10.3389/fpls.2022.1092411 (PMC9871781; doi:10.3389/fpls.2022.1092411)
Supplement: Supplementary file 1 [file DataSheet_1.pdf]

Supplementary table 1 Gene primer sequences in qRT-PCR

gene ID	Forward /Reverse primer	Primer sequence (5′to 3′)
gene356	Forward primer	AAGTTAGGAAAGGAAGCAGCAA
gene356	Reverse primer	GATGGACGGAGACCAAGGAG
gene31788	Forward primer	CCCCAGGTAGAGAAGCCATT
gene31788	Reverse primer	TCCCTAAGCAGGTGAAATGTAAG
gene33147	Forward primer	TTGCCGTCGGAGGAGAAG
gene33147	Reverse primer	CTTGACCCTTTTCGCTGACTAA
gene13565	Forward primer	TGTCTGCTGCTCATTTACATAGTTT
gene13565	Reverse primer	GCGAGCCATTATTGTTCTGC
gene16028	Forward primer	TTCATCACATAATGGTAAGCCTCT
gene16028	Reverse primer	AGCCAATTCTGAAAGGTACCC
gene2487	Forward primer	TTGGAGGTTGAGTTTGAGGG
gene2487	Reverse primer	AATATACAGCTCAGACGCACAGAT
gene9849	Forward primer	TGCTGCCCTTACTGTTCTGG
gene9849	Reverse primer	CAACATTTCCCTTACCAGCAGT
gene6161	Forward primer	GAAAACCGTCTTGTTGGTATGA
gene6161	Reverse primer	GTTTCTCGGTCAAGAGTTAAGGA
gene6396	Forward primer	TAAGGAGCCACAGGCAACA
gene6396	Reverse primer	CATCCTGTAGCCCCATACTTCT
gene20046	Forward primer	AAGCAGTGTTTGACTCTTGGGA
gene20046	Reverse primer	ATGTTTACCGCAGTTCCCTTTA
gene34286	Forward primer	ACATTTCTGGTGCCATCCTATT
gene34286	Reverse primer	CAGGGACAATGTTTTGCTCG
gene8143	Forward primer	GAACCCAATGGCAAAGGC
gene8143	Reverse primer	ATGACCACCTACAACACAGAGATTA
gene5361	Forward primer	GTACTGCAGGCATGGAGGC
gene5361	Reverse primer	TAGCCCCATCCAGAGTTCCT
gene15937	Forward primer	TACTCCTCACTCCTTTCCCATT
gene15937	Reverse primer	ATGGTAAAGAGATAAAGCGACAGA
gene17120	Forward primer	GTGAAGAAGGGCTTGTTATTGAG
gene17120	Reverse primer	GGACAGTCTCTCCACCATTTATCT
gene3183	Forward primer	GCTCACATTAGACTCAGGACCC
gene3183	Reverse primer	CCCCAAGAACATCACCAAGT
actin	Forward primer	CAAGGGTGAATACGATGAGTCTG
actin	Reverse primer	GCCTCTCACTAGCAGCCATAAC

Supplementary Table 2 Statistical results between the Clean reads and the reference genome

Sample	Reads Number	Mapped reads	Uniquely	Multiple mapped
Y0H1	49,308,454	93.83%	86.95%	6.88%
Y0H2	41,482,450	94.89%	87.74%	7.15%

Sample	Reads Number	Mapped reads	Uniquely	Multiple mapped
Y0H3	42,802,228	94.94%	82.30%	12.64%
Y2H1	56,373,462	95.19%	87.38%	7.81%
Y2H2	53,671,820	95.03%	88.74%	6.29%
Y2H3	51,349,860	95.17%	89.09%	6.08%
Y6H1	41,244,310	94.96%	87.70%	7.26%
Y6H2	64,264,466	95.28%	89.28%	6.01%
Y6H3	47,818,678	94.88%	85.98%	8.90%
Y20H1	43,687,722	94.09%	81.02%	13.07%
Y20H2	48,282,070	95.00%	81.81%	13.20%
Y20H3	43,816,428	85.43%	73.33%	12.10%
R0H1	56,306,220	94.44%	85.19%	9.26%
R0H2	50,484,566	94.60%	81.54%	13.06%
R0H3	48,269,086	94.61%	87.80%	6.81%
R2H1	42,255,064	94.88%	88.68%	6.20%
R2H2	44,814,618	94.80%	88.09%	6.71%
R2H3	42,392,590	94.53%	89.22%	5.31%
R6H1	44,966,122	94.82%	87.67%	7.15%
R6H2	42,764,428	94.75%	87.12%	7.62%
R6H3	42,557,914	94.31%	81.65%	12.66%
R20H1	44,696,864	94.57%	86.15%	8.43%
R20H2	49,722,064	94.86%	87.23%	7.63%
R20H3	48,736,668	94.53%	86.83%	7.70%

Supplementary Table 3 the common metabolites in Reyan8-79_vs_Yunyan77-4 groups

Index	Formula	Compounds	Class I	Class II	CAS	Level
pme3011	C4H9NO2	γ-Aminobutyric acid	Organic acids	Organic acids	-	B
pme0066	C3H7N3O2	Guanidinoacetate	Amino acids and derivatives	Amino acids and derivatives	352-97-6	B
mws0147	C5H10O3	β-Hydroxyisovaleric acid	Organic acids	Organic acids	625-08-1	B
pme1216	C6H5NO2	2-Picolinic acid	Organic acids	Organic acids	98-98-6	B
mws0263	C5H7NO3	5-Oxo-L-Proline	Amino acids and derivatives	Amino acids and derivatives	98-79-3	B

pmc0682	C8H8N2	4-Aminoindole	Alkaloids	Plumerane	5192-23-4	B
mws0972	C6H12O3	6-Hydroxyhexanoic acid	Organic acids	Organic acids	1191-25-9	B
Lmbn002862	C7H6O3	3-Hydroxybenzoic Acid	Organic acids	Organic acids	-	B
pme0295	C6H11NO3	4-Acetamidobutyric acid	Organic acids	Organic acids	3025-96-5	B
mws0847	C6H7N5	1-Methyladenine	Nucleotides and derivatives	Nucleotides and derivatives	5142-22-3	A
pme1266	C6H6N4O2	3-Methylxanthine	Nucleotides and derivatives	Nucleotides and derivatives	1076-22-8	B
mws0028	C8H8O4	Vanillic acid	Phenolic acids	Phenolic acids	121-34-6	A
mws0024	C7H6O5	Gallic acid	Tannins	Tannin	149-91-7	A
Lmgn002555	C8H14O4	2-Propylglutaric acid	Organic acids	Organic acids	32806-62-5	B
Lmbn001754	C7H12O5	3-Isopropylmalic Acid	Organic acids	Organic acids	921-28-8	B
pmb3101	C7H12O5	2-Isopropylmalic Acid	Organic acids	Organic acids	49601-06-1	B
Zmgn001448	C7H12O5	2-Propylmalic Acid	Organic acids	Organic acids	-	B
pmb0786	C6H13NO5	D-Glucosamine	Others	Saccharides and Alcohols	3416-24-8	B
pme0075	C7H11NO5	N-Acetyl-L-glutamic acid	Amino acids and derivatives	Amino acids and derivatives	1188-37-0	B
pme2993	C10H8O4	Scopoletin (7-Hydroxy-5-methoxycoumarin)	Lignans and Coumarins	Coumarins	92-61-5	A
pme1654	C12H18O3	Jasmonic acid	Organic acids	Organic acids	77026-92-7	B
Lmrj001698	C9H18N2O4	L-Seryl-L-Isoleucine	Amino acids and derivatives	Amino acids and derivatives	91086-51-0	B
mws4174	C8H15NO6	N-Acetyl-D-mannosamine	Others	Saccharides and Alcohols	7772-94-3	B
mws1639	C11H10O5	Isofraxidin	Lignans and Coumarins	Coumarins	486-21-5	A
mws4085	C11H12O5	Sinapic acid	Phenolic acids	Phenolic acids	530-59-6	A
Lmhp001430	C10H14N2O4	Cyclo(Pro-Glu)	Amino acids and derivatives	Amino acids and derivatives	-	B
pmb0490	C13H18N2O2	p-Coumaroylputrescine	Alkaloids	Phenolamine	34136-53-3	A
pmb2591	C13H14N2O3	N-Acetyl-L-Tryptophan	Amino acids and derivatives	Amino acids and derivatives	1218-34-4	A
pme2563	C8H14N2O5S	γ-Glu-Cys	Amino acids and derivatives	Amino acids and derivatives	636-58-8	B
Lmlp0031	C14H20N2	N-Feruloylputrescine	Alkaloids	Phenolamine	501-13-3	B

61	O3					
mws1060	C10H12N4O5	9-(Arabinosyl)hypoxanthine	Nucleotides and derivatives	Nucleotides and derivatives	7013-16-3	A
pmp000571	C15H10O5	Apigenin	Flavonoids	Flavonoid	520-36-5	A
Lmyn006227	C15H10O5	Galangin (3,5,7-Trihydroxyflavone)	Flavonoids	Flavonoid	548-83-4	B
Hmbp002730	C15H10O6	Isoscutellarein	Flavonoids	Flavonoid	41440-05-5	A
pme0088	C15H10O6	Luteolin (5,7,3',4'-Tetrahydroxyflavone)	Flavonoids	Flavonoid	491-70-3	A
mws0054	C15H14O6	Catechin	Flavonoids	Flavanols	154-23-4	A
pme0460	C15H14O6	Epicatechin	Flavonoids	Flavanols	490-46-0	B
mws2120	C13H8O8	Brevifolin carboxylic acid	Phenolic acids	Phenolic acids	18490-95-4	A
Lmqp000329	C8H14N3O7P	5-Aminoimidazole ribonucleotide	Nucleotides and derivatives	Nucleotides and derivatives	25635-88-5	A
Zmhn003257	C16H12O6	5,7,2'-Trhiyroxy-8-methoxyflavone	Flavonoids	Flavonoid	-	B
pmp000001	C16H12O6	Hispidulin (5,7,4'-Trihydroxy-6-methoxyflavone)	Flavonoids	Flavonoid	1447-88-7	A
Zmhp003514	C16H12O6	6,7,8-Tetrahydroxy-5-methoxyflavone	Flavonoids	Flavonoid	-	B
mws0058	C16H12O6	Diosmetin (5,7,3'-Trihydroxy-4'-methoxyflavone)	Flavonoids	Flavonoid	520-34-3	B
Zmhn000892	C13H18O8	4-O-Glucosyl-3,4-dihydroxybenzyl alcohol	Phenolic acids	Phenolic acids	-	B
mws0044	C15H12O7	Dihydroquercetin(Taxifolin)	Flavonoids	Dihydroflavonol	480-18-2	A
Hmqn003268	C15H12O7	5,7,3',4',5'-Pentahydroxydihydroflavone	Flavonoids	Dihydroflavone	-	B
pme2074	C18H29NO4	(-)-Jasmonoyl-L-Isoleucine	Organic acids	Organic acids	120330-93-0	B
Lmmn003663	C14H18O9	5-Glucosyloxy-2-Hydroxybenzoic acid methyl ester	Phenolic acids	Phenolic acids	-	A
Lmtn002565	C14H18O9	1-O-Vanilloyl-D-Glucose	Phenolic acids	Phenolic acids	-	A
Cmrn002205	C15H22O8	Bartsioside	Others	Others	62133-72-6	B
pmn001518	C13H16O10	1-O-Galloyl-D-glucose	Tannins	Tannin	58511-73-2	A

Lmtn0009 40	C14H18O1 0	1-O-(3,4-Dihydroxy-5-met hoxy-benzoyl)-glucoside	Phenolic acids	Phenolic acids	-	A
mws2108	C16H18O9	Cryptochlorogenic acid (4-O-Caffeoylquinic acid)	Phenolic acids	Phenolic acids	905-99-7	A
pme1816	C16H18O9	Neochlorogenic acid (5-O-Caffeoylquinic acid)	Phenolic acids	Phenolic acids	906-33-2	A
mws0178	C16H18O9	Chlorogenic acid (3-O-Caffeoylquinic acid)	Phenolic acids	Phenolic acids	327-97-9	A
Hmmn003 964	C16H24O9	7-Deoxyloganic acid	Terpenoids	Monoterpenoids	22487-36-1	B
Lmbp0022 55	C16H18O1 0	Fraxetin-8-O-glucoside (Fraxin)	Lignans and Coumarins	Coumarins	524-30-1	B
Lmbp0032 08	C17H20O1 0	Fraxidin-8-O-glucoside	Lignans and Coumarins	Coumarins	-	B
Zmhn0022 27	C17H22O1 0	4-O-Glucosyl-sinapate	Phenolic acids	Phenolic acids	-	B
pmn00142 3	C19H30O8	Roseoside	Others	Others	54835-70-0	B
Lmmn003 875	C21H24O7	Medioresinol	Lignans and Coumarins	Lignans	40957-99-1	B
pmb0580	C21H20O9	Chrysin-5-O-glucoside (Toringin)	Flavonoids	Flavonoid	1329-10-8	B
Lmbp0036 68	C20H18O1 0	Kaempferol-3-O-arabinosi de	Flavonoids	Flavonoid	-	A
pmn00163 7	C20H18O1 0	Kaempferol-3-O-arabinosi de (Juglanin)	Flavonoids	Flavonols	5041-67-8	A
HJN089	C21H20O1 0	Sophoricoside	Flavonoids	Isoflavones	152-95-4	B
pmp00041 3	C21H20O1 0	Genistein-8-C-glucoside	Flavonoids	Isoflavones	66026-80-0	A
Lmgp0047 31	C21H20O1 0	Genistein-7-O-galactoside	Flavonoids	Isoflavones	-	A
Lmlp0055 72	C21H20O1 0	Galangin-7-O-glucoside	Flavonoids	Flavonoid	-	A
pme0321	C21H20O1 0	Kaempferol-7-O-rhamnosi de	Flavonoids	Flavonols	20196-89-8	B
mws0919	C21H20O1 0	Kaempferol-3-O-rhamnosi de (Afzelin)(Kaempferin)	Flavonoids	Flavonols	482-39-3	A
Lmsn0024 94	C19H14O1 2	Ellagic acid-4-O-Xyloside	Tannins	Tannin	139163-18-1	B
mws4183	C20H18O1 1	Quercetin-3-O-arabinoside (Guaijaverin)	Flavonoids	Flavonols	22255-13-6	A
Lmfp0040 55	C20H18O1 1	Morin-3-O-xyloside	Flavonoids	Flavonols	-	B

Lmdp003509	C20H18O11	Quercetin-3-O-xyloside (Reynoutrin)	Flavonoids	Flavonols	549-32-6	A
mws2186	C20H18O11	Avicularin(Quercetin-3-O-α-L-arabinofuranoside)	Flavonoids	Flavonols	572-30-5	A
Lmfn004065	C20H18O11	Morin-3-O-arabinoside	Flavonoids	Flavonols	-	A
HJN090	C21H22O10	Butin-7-O-glucoside	Flavonoids	Flavonoid	-	A
Cmzn005251	C21H22O10	6-O-Caffeoylarbutin	Phenolic acids	Phenolic acids	136172-60-6	B
HJN087	C21H22O10	Naringenin-4'-O-glucoside	Flavonoids	Flavonoid	-	A
Lmlp006175	C21H22O10	Isosalipurposide (Phlorizin Chalcone)	Flavonoids	Chalcones	4547-85-7	B
mws1179	C21H22O10	Naringenin-7-O-glucoside (Prunin)	Flavonoids	Dihydroflavone	529-55-5	B
Smlp001915	C20H19O11+	Delphinidin-3-O-arabinoside	Flavonoids	Anthocyanins	-	B
pmn001477	C20H20O11	4-C-Glucose-1,3,6-trihydroxy-7-methoxyxanthone	Others	Xanthone	-	B
Lmfn004093	C21H24O10	Phloretin-4'-O-glucoside (Trilobatin)	Flavonoids	Chalcones	4192-90-9	A
pmp000575	C22H22O10	Acacetin-7-O-glucoside (Tilianin)	Flavonoids	Flavonoid	4291-60-5	B
pmp000573	C22H22O10	Acacetin-7-O-galactoside	Flavonoids	Flavonoid	-	B
Hmcp002316	C21H20O11	Isorhamnetin-3-O-arabinoside	Flavonoids	Flavonols	-	A
Lmlp003531	C21H20O11	Luteolin-3'-O-glucoside	Flavonoids	Flavonoid	5154-41-6	A
mws1299	C21H20O11	Luteolin-8-C-glucoside (Orientin)	Flavonoids	Flavonoid carbonoside	28608-75-5	B
mws1608	C21H20O11	Luteolin-6-C-glucoside (Isoorientin)	Flavonoids	Flavonoid carbonoside	4261-42-1	B
mws0913	C21H20O11	Kaempferol-3-O-galactoside (Trifolin)	Flavonoids	Flavonols	23627-87-4	A
mws0045	C21H20O11	Quercetin-3-O-rhamnoside (Quercitrin)	Flavonoids	Flavonols	522-12-3	B
pmb0550	C21H21O11+	Cyanidin-3-O-glucoside (Kuromanin)	Flavonoids	Anthocyanins	47705-70-4	B
pmf0027	C21H21O11+	Cyanidin-3-O-galactoside	Flavonoids	Anthocyanins	142506-26-1	B
Lmtn002796	C21H22O11	Aromadendrin-7-O-glucoside	Flavonoids	Flavonoid	28189-90-4	B

HJN041	C21H24O11	Epicatechin glucoside	Flavonoids	Flavanols	-	B
mws0351	C22H18O11	Gallate catechin gallate	Flavonoids	Flavanols	4233-96-9	B
mws0034	C22H18O11	Epigallocatechin-3-gallate	Flavonoids	Flavanols	989-51-5	B
pmp000579	C22H22O11	Diosmetin-7-O-galactoside	Flavonoids	Flavonoid	-	A
Lmpn006208	C22H22O11	8-Methoxykaempferol-7-O-rhamnoside	Flavonoids	Flavonols	-	B
pmb3012	C22H22O11	Chrysoeriol-7-O-glucoside	Flavonoids	Flavonoid	19993-32-9	A
Lmjp003655	C22H22O11	6-C-MethylKaempferol-3-glucoside	Flavonoids	Flavonoid	-	A
Hmgp002189	C22H22O11	Hispidulin-7-O-Glucoside	Flavonoids	Flavonoid	17680-84-1	A
Hmdn001667	C20H16O13	Ellagic acid-4-O-glucoside	Tannins	Tannin	-	B
Lmzp002365	C22H24O11	Hesperetin-7-O-glucoside	Flavonoids	Flavonoid	31712-49-9	B
Lmhp010162	C22H46NO7P	LysoPE 17:0	Lipids	LPE	-	B
HJAP056	C23H24O11	Dihydroxy-dimethoxyflavone-7-O-glucoside	Flavonoids	Flavonoid	-	B
pme3391	C22H23O12+	Petunidin-3-O-glucoside	Flavonoids	Anthocyanins	6988-81-4	B
Smpp001442	C23H27O11+	Delphinidin-3-O-glucuronide	Flavonoids	Anthocyanins	-	B
Lmpp003465	C21H20O13	Myricetin-3-O-glucoside	Flavonoids	Flavonols	19833-12-6	B
Lmmn000214	C18H32O15	Solatriose	Others	Saccharides and Alcohols	528-40-5	B
Lmmn003398	C23H22O12	Kaempferol-3-O-(6"-acetyl)glucoside	Flavonoids	Flavonols	-	A
Hmln002199	C23H22O13	Quercetin-3-O-(6"-acetyl)galactoside	Flavonoids	Flavonols	-	B
HJAP006	C23H24O13	Syringetin-7-O-glucoside	Flavonoids	Flavonoid	-	B
Lmhp010573	C27H48O9	1-Linoleoylglycerol-3-O-glucoside	Lipids	Glycerol ester	-	B
pmn001378	C26H32O11	Pinoresinol-4-O-glucoside	Lignans and Coumarins	Lignans	41607-20-9	A
Cmsp005051	C26H34O11	(+)-Isolariciresinol-9-O-glucoside	Lignans and Coumarins	Lignans	-	B

Lmmn002274	C26H34O11	Isolariciresinol-9'-O-glucoside	Lignans and Coumarins	Lignans	63358-12-3	B
HJN083	C26H34O11	Lariciresinol-4'-O-glucoside	Lignans and Coumarins	Lignans	143663-00-7	B
Lmtn003096	C26H36O11	(-)-Secoisolariciresinol 4-O-glucoside	Lignans and Coumarins	Lignans	-	B
Lmhp007598	C27H52NO7P	LysoPC 19:2(2n isomer)	Lipids	LPC	-	B
Lmmp003817	C24H22O14	Kaempferol-3-O-(6"-malonyl)glucoside	Flavonoids	Flavonols	-	A
Lmdp004892	C24H22O14	Kaempferol-3-O-(6"-malonyl)galactoside	Flavonoids	Flavonols	-	A
Cmcp006260	C30H18O10	Robustaflavone	Flavonoids	Biflavones	49620-13-5	A
pmp000589	C24H22O15	Quercetin-7-O-(6"-malonyl)glucoside	Flavonoids	Flavonols	-	B
Hmln002189	C24H22O15	Quercetin-3-O-(6"-malonyl)galactoside	Flavonoids	Flavonols	-	B
Lmmn002260	C27H36O12	5'-Methoxyisolariciresinol-9'-O-glucoside	Lignans and Coumarins	Lignans	-	B
HJN056	C30H38O10	Sesquimarocanol B	Others	Others	-	B
Lmhn003802	C26H26O14	Sinapoylsinapoyltartaric acid	Phenolic acids	Phenolic acids	-	B
pme0436	C30H26O12	Procyanidin B3	Tannins	Proanthocyanidins	23567-23-9	A
mws0836	C30H26O12	Procyanidin B1	Tannins	Proanthocyanidins	20315-25-7	A
pmn001667	C30H26O12	Procyanidin B4	Tannins	Proanthocyanidins	29106-51-2	B
pme0434	C30H26O12	Procyanidin B2	Tannins	Proanthocyanidins	29106-49-8	A
Lmgp004474	C27H30O14	Genistein-7-O-galactoside-rhamnose	Flavonoids	Isoflavones	-	B
pme2493	C27H30O14	Kaempferol-3,7-O-dirhamnoside (Kaempferitrin)	Flavonoids	Flavonols	482-38-2	A
pme0368	C27H30O14	Apigenin-7-O-rutinoside (Isorhoifolin)	Flavonoids	Flavonoid	552-57-8	B
Smsp002643	C27H31O14+	Pelargonidin-3-O-rutinoside	Flavonoids	Anthocyanins	-	B
Lmcn003163	C28H20O14	Epitheaflavic acid-3-O-Gallate	Tannins	Tannin	34218-97-8	B
Hmcp001769	C26H28O15	Quercetin-3-O-rhamnosyl(1→2)arabinoside	Flavonoids	Flavonols	-	B

pmb0631	C26H28O15	Luteolin-8-C-glucoside-6-C-arabinoside	Flavonoids	Flavonoid carbonoside	-	B
Lmjp001877	C26H29O15+	Cyanidin-3-O-sambubioside [Cyanidin-3-O-(2''-O-xylosyl)glucoside]	Flavonoids	Anthocyanins	63535-17-1	B
pmb2936	C28H32O14	Disinapoyl glucoside	Phenolic acids	Phenolic acids	-	B
Hmjn004446	C30H26O13	Luteolin-7-O-(6''-caffeoyl)rhamnoside	Flavonoids	Flavonoid	-	A
mws1290	C30H26O13	Kaempferol-3-O-(6''-p-Coumaroyl)glucoside (Tiliroside)	Flavonoids	Flavonols	20316-62-5	A
Lmyp004318	C30H26O13	Kaempferol-3-O-(6''-p-Coumaroyl)galactoside	Flavonoids	Flavonoid	-	A
Lmyp004407	C30H26O13	Kaempferol-3-O-(2''-p-Coumaroyl)galactoside	Flavonoids	Flavonoid	-	B
mws0791	C28H34O14	Poncirin (Isosakuranetin-7-O-neohesperidoside)	Flavonoids	Dihydroflavone	14941-08-3	B
pme1793	C27H31O15+	Pelargonidin-3,5-O-diglucoside	Flavonoids	Anthocyanins	17334-58-6	B
Lmyp003500	C28H24O15	Kaempferol-3-O-(2''-galloyl)galactoside	Flavonoids	Flavonoid	-	A
Lmyp003348	C28H24O15	Kaempferol-3-O-(6''-galloyl)galactoside	Flavonoids	Flavonoid	-	B
Lmyp003599	C28H24O15	Kaempferol-3-O-(6''-galloyl)glucoside	Flavonoids	Flavonoid	56317-05-6	A
Lmyp004052	C30H26O14	Quercetin-3-O-(6''-p-Coumaroyl)galactoside	Flavonoids	Flavonols	-	A
Lmdp004267	C30H26O14	Quercetin-3-O-(6''-p-Coumaroyl)glucoside	Flavonoids	Flavonols	-	A
pmp000126	C27H30O16	Luteolin-6,8-di-C-glucoside	Flavonoids	Flavonoid carbonoside	29428-58-8	B
HJAP024	C27H30O16	Kaempferol-6,8-di-C-glucoside	Flavonoids	Flavonoid	-	B
Lmyp003349	C28H24O16	Quercetin-3-O-(6''-galloyl)glucoside	Flavonoids	Flavonols	56316-75-7	A
Zmdp003457	C27H30O17	Myricetin-3-O-galactoside-3'-O-rhamnoside	Flavonoids	Flavonoid	-	A
Lmtp003677	C27H30O17	Quercetin-3-O-sophoroside (Baimaside)	Flavonoids	Flavonols	18609-17-1	B
Hmlp003068	C27H30O17	Meratin	Flavonoids	Flavonoid	27215-04-9	A

Lmsp003729	C27H30O17	Myricetin-3-O-rutinoside	Flavonoids	Flavonols	-	A
pmb0618	C28H34O16	Hesperetin-8-C-glucoside-3'-O-glucoside	Flavonoids	Flavonoid carbonoside	-	B
pmb0645	C28H34O16	Hesperetin-6-C-glucoside-7-O-glucoside	Flavonoids	Flavonoid carbonoside	-	B
Lmcp005542	C30H27O15+	Delphinidin-3-O-(6''-O-caffeoyl)glucoside	Flavonoids	Anthocyanins	-	B
Lmsn002110	C27H22O18	Corilagin	Tannins	Tannin	23094-69-1	B
pmn001622	C27H22O18	1-O-Galloyl-4,6-(-)-hexahydroxydiphenoyl-D-glucose	Phenolic acids	Phenolic acids	126721-55-9	A
Zmhn003541	C27H22O18	Gemin D	Tannins	Tannin	84744-46-7	A
pmb2653	C24H42O20	D(+)-Melezitose O-rhamnoside	Others	Saccharides and Alcohols	-	B
pmp001271	C33H58O14	1-Linoleoyl-sn-glycerol-diglucoside	Lipids	Free fatty acids	-	B
Lmhp009384	C33H58O14	1-Linoleoylglycerol-2,3-di-O-glucoside	Lipids	Glycerol ester	-	B
HJN094	C37H30O16	Galloylprocyanidin B4	Tannins	Proanthocyanidins	-	B
HJAP061	C32H38O20	Quercetin-3-O-(2''-O-arabinosyl)rutinoside	Flavonoids	Flavonols	-	B
pmp001314	C33H40O21	6-Hydroxykaempferol-3-O-rutin-6-O-glucoside	Flavonoids	Flavonols	-	A
Zmhn003098	C38H42O18	4-O-(6'-O-Glucosylcaffeoylglucosylferuloyl)-4-hydroxybenzyl alcohol	Phenolic acids	Phenolic acids	-	B
pmn001534	C34H28O22	1,2,3,6-Tetra-O-Galloyl-D-Glucose	Phenolic acids	Phenolic acids	79886-50-3	A
pmp000100	C45H38O18	Arecatannin B1	Tannins	Tannin	79763-28-3	A
pmb2947	C45H38O18	Catechin-catechin-catechin	Flavonoids	Flavanols	-	A
pmn001647	C45H38O18	Procyanidin C2	Tannins	Proanthocyanidins	37064-31-6	A
Lmmp002068	C45H38O19	Gallocatechin-catechin-catechin	Tannins	Proanthocyanidins	-	B
Lmmp001653	C45H38O20	Gallocatechin-gallocatechin-catechin	Tannins	Proanthocyanidins	-	B
Lmqp002161	C39H51O25+	Cyanidin-3-O-rutinoside-5,3'-di-O-glucoside	Flavonoids	Anthocyanins	-	B

Supplementary Table4 the different types and contents of flavonoids in different treatment point

(A) at R0H_vs_Y0H

Compounds	Class I	Class II	Y0H1	Y0H2	Y0H3	R0H1	R0H2	R0H3	VIP	Fold_Change	Log2FC	Type
Apigenin	Flavonoids	Flavonoid	2.48E+04	2.28E+04	1.22E+04	1.11E+04	7.53E+03	5.88E+03	1.20E+00	4.10E-01	-1.29E+00	up
Galangin (3,5,7-Trihydroxyflavone)	Flavonoids	Flavonoid	5.46E+04	4.62E+04	4.32E+04	1.89E+04	2.29E+04	1.39E+04	1.34E+00	3.87E-01	-1.37E+00	up
Isoscutellarein	Flavonoids	Flavonoid	1.12E+04	6.93E+03	4.02E+03	3.69E+03	4.08E+03	3.20E+03	1.02E+00	4.95E-01	-1.01E+00	up
Luteolin (5,7,3',4'-Tetrahydroxyflavone)	Flavonoids	Flavonoid	4.27E+04	2.82E+04	1.37E+04	8.52E+03	1.69E+04	8.90E+03	1.02E+00	4.06E-01	-1.30E+00	up
Catechin	Flavonoids	Flavanols	2.35E+06	1.66E+06	8.90E+05	2.52E+05	8.30E+05	3.33E+05	1.15E+00	2.89E-01	-1.79E+00	up
Epicatechin	Flavonoids	Flavanols	3.33E+06	2.28E+06	1.34E+06	3.88E+05	8.35E+05	5.35E+05	1.25E+00	2.53E-01	-1.98E+00	up
5,7,2'-Trhiyroxy-8-methoxyflavone	Flavonoids	Flavonoid	2.53E+05	2.10E+05	1.53E+05	9.45E+04	1.34E+05	7.93E+04	1.21E+00	5.00E-01	-1.00E+00	up
Hispidulin (5,7,4'-Trihydroxy-6-methoxyflavone)	Flavonoids	Flavonoid	2.08E+05	1.73E+05	1.11E+05	6.58E+04	1.08E+05	5.43E+04	1.15E+00	4.63E-01	-1.11E+00	up
6,7,8-Tetrahydroxy-5-methoxyflavone	Flavonoids	Flavonoid	2.15E+05	1.78E+05	1.25E+05	6.03E+04	1.10E+05	5.99E+04	1.19E+00	4.45E-01	-1.17E+00	up
Diosmetin (5,7,3'-Trihydroxy-4'-methoxyflavone)	Flavonoids	Flavonoid	2.83E+05	2.30E+05	1.71E+05	1.05E+05	1.49E+05	8.66E+04	1.20E+00	4.99E-01	-1.00E+00	up
Dihydroquercetin(Taxifolin)	Flavonoids	Dihydroflavonol	5.68E+05	1.94E+05	1.24E+05	2.53E+04	2.15E+04	1.32E+04	1.32E+00	6.77E-02	-3.89E+00	up
5,7,3',4',5'-Pentahydroxydihydroflavone	Flavonoids	Dihydroflavone	2.52E+05	5.88E+04	7.77E+04	6.71E+03	4.09E+04	1.43E+04	1.13E+00	1.59E-01	-2.65E+00	up
Chrysin-5-O-glucoside (Toringin)	Flavonoids	Flavonoid	2.96E+05	2.52E+05	1.38E+05	5.42E+04	1.33E+05	5.15E+04	1.16E+00	3.48E-01	-1.52E+00	up
Kaempferol-3-O-arabinoside	Flavonoids	Flavonoid	8.51E+05	7.95E+05	7.85E+05	3.48E+05	4.05E+05	3.27E+05	1.39E+00	4.44E-01	-1.17E+00	up
Kaempferol-3-O-arabinoside (Juglanin)	Flavonoids	Flavonols	9.82E+04	9.36E+04	1.01E+05	4.84E+04	4.52E+04	3.59E+04	1.38E+00	4.43E-01	-1.17E+00	up
Sophoricoside	Flavonoids	Isoflavones	1.20E+06	1.44E+06	1.12E+06	4.18E+05	5.26E+05	4.17E+05	1.37E+00	3.62E-01	-1.47E+00	up
Genistein-8-C-glucoside	Flavonoids	Isoflavones	1.01E+07	8.44E+06	9.16E+06	3.79E+06	5.22E+06	4.13E+06	1.35E+00	4.75E-01	-1.08E+00	up

Genistein-7-O-galactoside	Flavonoids	Isoflavones	1.49E+07	1.61E+07	1.11E+07	4.32E+06	5.08E+06	4.32E+06	1.37E+00	3.25E-01	-1.62E+00	up
Galangin-7-O-glucoside	Flavonoids	Flavonoid	1.52E+06	1.35E+06	1.53E+06	4.73E+05	6.61E+05	5.76E+05	1.37E+00	3.89E-01	-1.36E+00	up
Kaempferol-7-O-rhamnoside	Flavonoids	Flavonols	2.28E+05	2.41E+05	1.89E+05	4.65E+04	1.62E+04	8.27E+03	1.32E+00	1.08E-01	-3.21E+00	up
Kaempferol-3-O-rhamnoside (Afzelin)(Kaempferin)	Flavonoids	Flavonols	2.98E+05	3.02E+05	2.44E+05	5.73E+04	2.49E+04	1.32E+04	1.34E+00	1.13E-01	-3.15E+00	up
Quercetin-3-O-arabinoside (Guaijaverin)	Flavonoids	Flavonols	2.18E+06	1.97E+06	2.32E+06	3.09E+05	4.12E+05	4.68E+05	1.38E+00	1.84E-01	-2.44E+00	up
Morin-3-O-xyloside	Flavonoids	Flavonols	5.47E+06	5.51E+06	5.73E+06	1.14E+06	1.29E+06	1.31E+06	1.40E+00	2.24E-01	-2.16E+00	up
Quercetin-3-O-xyloside (Reynoutrin)	Flavonoids	Flavonols	4.70E+06	4.71E+06	4.66E+06	1.07E+06	1.26E+06	1.18E+06	1.40E+00	2.49E-01	-2.00E+00	up
Avicularin(Quercetin-3-O-α-L-arabino furanoside)	Flavonoids	Flavonols	4.69E+06	4.89E+06	4.74E+06	1.06E+06	1.11E+06	1.19E+06	1.40E+00	2.35E-01	-2.09E+00	up
Morin-3-O-arabinoside	Flavonoids	Flavonols	3.44E+06	3.65E+06	3.97E+06	8.34E+05	8.82E+05	8.72E+05	1.40E+00	2.34E-01	-2.10E+00	up
Butin-7-O-glucoside	Flavonoids	Flavonoid	1.26E+06	6.83E+05	5.27E+05	1.32E+05	1.48E+05	1.33E+05	1.35E+00	1.67E-01	-2.58E+00	up
Naringenin-4'-O-glucoside	Flavonoids	Flavonoid	1.25E+06	6.61E+05	5.38E+05	1.54E+05	1.61E+05	1.37E+05	1.35E+00	1.85E-01	-2.44E+00	up
Isosalipurposide (Phlorizin Chalcone)	Flavonoids	Chalcones	8.99E+05	4.36E+05	3.62E+05	1.01E+05	9.12E+04	8.46E+04	1.34E+00	1.63E-01	-2.62E+00	up
Naringenin-7-O-glucoside (Prunin)	Flavonoids	Dihydroflavo ne	8.32E+05	4.50E+05	2.91E+05	1.58E+05	3.10E+05	1.63E+05	1.07E+00	4.01E-01	-1.32E+00	up
Delphinidin-3-O-arabinoside	Flavonoids	Anthocyanins	2.95E+05	2.30E+05	1.92E+05	3.70E+04	1.57E+05	3.69E+04	1.14E+00	3.22E-01	-1.64E+00	up
Phloretin-4'-O-glucoside (Trilobatin)	Flavonoids	Chalcones	5.21E+06	5.15E+06	1.76E+06	3.61E+05	1.44E+06	4.72E+05	1.18E+00	1.88E-01	-2.41E+00	up
Acacetin-7-O-glucoside (Tilianin)	Flavonoids	Flavonoid	9.19E+04	9.31E+04	4.03E+04	1.10E+04	1.78E+04	1.38E+04	1.31E+00	1.89E-01	-2.40E+00	up
Acacetin-7-O-galactoside	Flavonoids	Flavonoid	1.12E+05	1.25E+05	5.86E+04	1.42E+04	3.28E+04	1.54E+04	1.28E+00	2.11E-01	-2.25E+00	up
Isorhamnetin-3-O-arabinoside	Flavonoids	Flavonols	6.37E+04	7.08E+04	4.27E+04	2.22E+04	2.71E+04	2.17E+04	1.32E+00	4.00E-01	-1.32E+00	up
Luteolin-3'-O-glucoside	Flavonoids	Flavonoid	4.54E+06	4.79E+06	2.91E+06	8.91E+06	1.07E+07	8.90E+06	1.31E+00	2.33E+00	1.22E+00	down
Luteolin-8-C-glucoside (Orientin)	Flavonoids	Flavonoid carbonoside	3.93E+05	4.24E+05	3.74E+05	8.45E+04	1.62E+05	1.64E+05	1.29E+00	3.44E-01	-1.54E+00	up
Luteolin-6-C-glucoside (Isoorientin)	Flavonoids	Flavonoid	1.68E+05	1.76E+05	1.25E+05	2.30E+04	6.11E+04	3.31E+04	1.29E+00	2.50E-01	-2.00E+00	up

		carbonoside									
Kaempferol-3-O-galactoside (Trifolin)	Flavonoids	Flavonols	4.53E+05	3.73E+05	5.07E+05	1.13E+05	9.36E+04	1.02E+05	1.40E+00	2.31E-01	-2.11E+00	up
Quercetin-3-O-rhamnoside(Quercitrin)	Flavonoids	Flavonols	2.85E+06	2.62E+06	2.67E+06	3.22E+04	2.08E+04	3.25E+04	1.40E+00	1.05E-02	-6.57E+00	up
Cyanidin-3-O-glucoside (Kuromanin)	Flavonoids	Anthocyanins	3.75E+06	2.84E+06	7.85E+06	1.21E+06	9.92E+05	8.30E+05	1.30E+00	2.10E-01	-2.25E+00	up
Cyanidin-3-O-galactoside	Flavonoids	Anthocyanins	3.81E+06	3.07E+06	8.39E+06	1.35E+06	1.33E+06	6.99E+05	1.26E+00	2.22E-01	-2.17E+00	up
Aromadendrin-7-O-glucoside	Flavonoids	Flavonoid	5.12E+06	6.51E+06	7.89E+06	2.71E+06	2.75E+06	1.85E+06	1.33E+00	3.74E-01	-1.42E+00	up
Epicatechin glucoside	Flavonoids	Flavanols	7.19E+06	5.52E+06	3.88E+06	8.64E+05	1.71E+06	1.10E+06	1.32E+00	2.22E-01	-2.17E+00	up
Gallate catechin gallate	Flavonoids	Flavanols	1.38E+06	6.05E+05	2.69E+05	2.33E+04	2.34E+04	1.38E+04	1.36E+00	2.69E-02	-5.22E+00	up
Epigallocatechin-3-gallate	Flavonoids	Flavanols	1.22E+06	7.17E+05	3.10E+05	1.60E+04	1.85E+04	1.88E+04	1.37E+00	2.37E-02	-5.40E+00	up
Diosmetin-7-O-galactoside	Flavonoids	Flavonoid	1.98E+07	1.68E+07	1.00E+07	4.32E+06	8.18E+06	4.87E+06	1.22E+00	3.72E-01	-1.43E+00	up
8-Methoxykaempferol-7-O-rhamnoside	Flavonoids	Flavonols	1.58E+04	1.83E+04	1.12E+04	9.00E+00	8.03E+03	9.00E+00	1.05E+00	1.78E-01	-2.49E+00	up
Chrysoeriol-7-O-glucoside	Flavonoids	Flavonoid	3.85E+06	3.38E+06	2.11E+06	6.70E+05	1.38E+06	7.49E+05	1.27E+00	3.00E-01	-1.74E+00	up
6-C-MethylKaempferol-3-glucoside	Flavonoids	Flavonoid	2.03E+07	1.70E+07	1.01E+07	4.14E+06	7.99E+06	4.68E+06	1.22E+00	3.55E-01	-1.49E+00	up
Hispidulin-7-O-Glucoside	Flavonoids	Flavonoid	1.86E+07	1.62E+07	9.70E+06	4.15E+06	7.61E+06	4.81E+06	1.23E+00	3.72E-01	-1.43E+00	up
Hesperetin-7-O-glucoside	Flavonoids	Flavonoid	4.61E+06	4.05E+06	4.43E+06	1.73E+06	1.45E+06	1.62E+06	1.40E+00	3.67E-01	-1.45E+00	up
Dihydroxy-dimethoxyflavone-7-O-glucoside	Flavonoids	Flavonoid	2.49E+04	3.11E+04	2.00E+04	9.62E+03	1.41E+04	1.03E+04	1.28E+00	4.47E-01	-1.16E+00	up
Petunidin-3-O-glucoside	Flavonoids	Anthocyanins	6.10E+04	8.28E+04	8.64E+04	9.00E+00	9.00E+00	9.00E+00	1.41E+00	1.17E-04	-1.31E+01	up
Delphinidin-3-O-glucuronide	Flavonoids	Anthocyanins	1.07E+05	8.59E+04	5.71E+04	3.05E+04	4.09E+04	3.12E+04	1.27E+00	4.10E-01	-1.28E+00	up
Myricetin-3-O-glucoside	Flavonoids	Flavonols	2.44E+05	1.81E+05	2.67E+05	6.94E+04	5.10E+04	5.02E+04	1.38E+00	2.46E-01	-2.02E+00	up
Kaempferol-3-O-(6"-acetyl)glucoside	Flavonoids	Flavonols	4.25E+05	3.10E+05	4.59E+05	8.04E+04	9.23E+04	7.21E+04	1.39E+00	2.05E-01	-2.29E+00	up
Quercetin-3-O-(6"-acetyl)galactoside	Flavonoids	Flavonols	3.18E+04	2.37E+04	3.19E+04	6.10E+03	6.30E+03	7.12E+03	1.39E+00	2.23E-01	-2.16E+00	up
Syringetin-7-O-glucoside	Flavonoids	Flavonoid	2.63E+04	2.11E+04	1.88E+04	4.96E+03	1.73E+04	1.08E+04	1.00E+00	4.99E-01	-1.00E+00	up
Kaempferol-3-O-(6"-malonyl)glucoside	Flavonoids	Flavonols	8.34E+05	7.89E+05	1.01E+06	2.15E+05	1.63E+05	1.74E+05	1.40E+00	2.10E-01	-2.25E+00	up

Kaempferol-3-O-(6"-malonyl)galactoside	Flavonoids	Flavonols	8.06E+05	8.56E+05	9.62E+05	2.45E+05	1.79E+05	1.67E+05	1.39E+00	2.25E-01	-2.15E+00	up
Robustaflavone	Flavonoids	Biflavones	9.03E+03	6.35E+03	5.41E+03	3.49E+03	4.50E+03	2.15E+03	1.16E+00	4.88E-01	-1.04E+00	up
Quercetin-7-O-(6"-malonyl)glucoside	Flavonoids	Flavonols	1.61E+05	1.58E+05	1.61E+05	3.50E+04	3.55E+04	3.99E+04	1.40E+00	2.30E-01	-2.12E+00	up
Quercetin-3-O-(6"-malonyl)galactoside	Flavonoids	Flavonols	7.38E+04	7.23E+04	8.19E+04	1.41E+04	1.17E+04	1.50E+04	1.40E+00	1.79E-01	-2.48E+00	up
Genistein-7-O-galactoside-rhamnose	Flavonoids	Isoflavones	8.70E+04	1.13E+05	1.21E+05	2.02E+04	2.41E+04	1.91E+04	1.39E+00	1.97E-01	-2.34E+00	up
Kaempferol-3,7-O-dirhamnoside (Kaempferitrin)	Flavonoids	Flavonols	2.68E+06	1.31E+06	4.57E+05	6.02E+04	2.57E+05	8.99E+04	1.22E+00	9.17E-02	-3.45E+00	up
Apigenin-7-O-rutinoside (Isorhoifolin)	Flavonoids	Flavonoid	1.13E+05	1.41E+05	1.30E+05	1.46E+04	1.73E+04	1.32E+04	1.40E+00	1.18E-01	-3.09E+00	up
Pelargonidin-3-O-rutinoside	Flavonoids	Anthocyanins	4.84E+06	3.77E+06	1.53E+06	3.40E+05	1.16E+06	5.06E+05	1.19E+00	1.98E-01	-2.34E+00	up
Quercetin-3-O-rhamnosyl(1→2)arabinoside	Flavonoids	Flavonols	5.09E+04	2.24E+04	1.17E+04	6.30E+03	1.37E+04	5.69E+03	1.04E+00	3.02E-01	-1.73E+00	up
Luteolin-8-C-glucoside-6-C-arabinoside	Flavonoids	Flavonoid carbonoside	8.97E+03	5.27E+03	6.47E+03	9.00E+00	2.86E+03	9.00E+00	1.09E+00	1.39E-01	-2.85E+00	up
Cyanidin-3-O-sambubioside [Cyanidin-3-O-(2"-O-xylosyl)glucoside]	Flavonoids	Anthocyanins	1.13E+05	7.28E+04	3.78E+04	7.05E+03	3.06E+04	1.36E+04	1.15E+00	2.30E-01	-2.12E+00	up
Luteolin-7-O-(6"-caffeoyl)rhamnoside	Flavonoids	Flavonoid	1.05E+05	8.87E+04	7.93E+04	8.89E+03	1.19E+04	1.45E+04	1.38E+00	1.29E-01	-2.96E+00	up
Kaempferol-3-O-(6"-p-Coumaroyl)glucoside (Tiliroside)	Flavonoids	Flavonols	2.52E+05	2.17E+05	1.92E+05	3.79E+04	3.67E+04	2.35E+04	1.39E+00	1.48E-01	-2.75E+00	up
Kaempferol-3-O-(6"-p-Coumaroyl)galactoside	Flavonoids	Flavonoid	5.27E+04	6.22E+04	7.66E+04	9.00E+00	9.00E+00	9.00E+00	1.41E+00	1.41E-04	-1.28E+01	up
Kaempferol-3-O-(2"-p-Coumaroyl)galactoside	Flavonoids	Flavonoid	5.32E+04	6.76E+04	6.72E+04	9.00E+00	9.00E+00	9.00E+00	1.41E+00	1.44E-04	-1.28E+01	up
Poncirin	Flavonoids	Dihydroflavo	2.98E+05	3.67E+05	2.19E+05	3.95E+04	3.68E+04	3.45E+04	1.39E+00	1.25E-01	-2.99E+00	up

(Isosakuranetin-7-O-neohesperidoside)		ne										
Pelargonidin-3,5-O-diglucoside	Flavonoids	Anthocyanins	1.09E+05	9.00E+04	6.58E+04	2.01E+04	3.16E+04	2.74E+04	1.33E+00	2.99E-01	-1.74E+00	up
Kaempferol-3-O-(2''-galloyl)galactoside	Flavonoids	Flavonoid	2.11E+04	1.43E+04	7.91E+03	9.00E+00	9.00E+00	9.00E+00	1.40E+00	6.23E-04	-1.06E+01	up
Kaempferol-3-O-(6''-galloyl)galactoside	Flavonoids	Flavonoid	1.78E+04	2.62E+04	7.70E+03	4.89E+03	9.00E+00	9.00E+00	1.13E+00	9.49E-02	-3.40E+00	up
Kaempferol-3-O-(6''-galloyl)glucoside	Flavonoids	Flavonoid	1.80E+04	2.80E+04	1.17E+04	7.01E+03	5.81E+03	1.61E+03	1.17E+00	2.50E-01	-2.00E+00	up
Quercetin-3-O-(6''-p-Coumaroyl)galactoside	Flavonoids	Flavonols	9.21E+04	1.02E+05	7.46E+04	1.61E+04	1.77E+04	1.08E+04	1.39E+00	1.66E-01	-2.59E+00	up
Quercetin-3-O-(6''-p-Coumaroyl)glucoside	Flavonoids	Flavonols	8.96E+04	9.38E+04	6.36E+04	1.73E+04	1.71E+04	1.16E+04	1.38E+00	1.86E-01	-2.43E+00	up
Luteolin-6,8-di-C-glucoside	Flavonoids	Flavonoid carbonoside	1.63E+05	1.77E+05	9.43E+04	5.34E+04	7.03E+04	6.08E+04	1.25E+00	4.25E-01	-1.23E+00	up
Kaempferol-6,8-di-C-glucoside	Flavonoids	Flavonoid	8.54E+04	9.71E+04	9.21E+04	3.11E+04	2.99E+04	3.65E+04	1.39E+00	3.55E-01	-1.49E+00	up
Quercetin-3-O-(6''-galloyl)glucoside	Flavonoids	Flavonols	8.70E+03	7.86E+03	5.02E+03	9.00E+00	9.00E+00	9.00E+00	1.40E+00	1.25E-03	-9.64E+00	up
Myricetin-3-O-galactoside-3'-O-rhamnoside	Flavonoids	Flavonoid	5.06E+06	5.28E+06	4.14E+06	1.99E+06	2.26E+06	2.57E+06	1.34E+00	4.72E-01	-1.08E+00	up
Quercetin-3-O-sophoroside (Baimaside)	Flavonoids	Flavonols	9.97E+05	1.13E+06	1.14E+06	4.34E+05	4.51E+05	4.87E+05	1.39E+00	4.19E-01	-1.25E+00	up
Meratin	Flavonoids	Flavonoid	1.65E+06	1.26E+06	1.50E+06	7.03E+05	6.91E+05	7.50E+05	1.37E+00	4.86E-01	-1.04E+00	up
Myricetin-3-O-rutinoside	Flavonoids	Flavonols	4.44E+06	5.28E+06	4.07E+06	2.08E+06	2.35E+06	2.46E+06	1.35E+00	4.99E-01	-1.00E+00	up
Hesperetin-8-C-glucoside-3'-O-glucoside	Flavonoids	Flavonoid carbonoside	7.55E+05	7.57E+05	8.86E+05	3.93E+05	3.51E+05	3.76E+05	1.39E+00	4.67E-01	-1.10E+00	up
Hesperetin-6-C-glucoside-7-O-glucoside	Flavonoids	Flavonoid carbonoside	8.90E+05	9.22E+05	9.68E+05	4.15E+05	4.14E+05	4.25E+05	1.40E+00	4.51E-01	-1.15E+00	up
Delphinidin-3-O-(6''-O-caffeoyl)gluco	Flavonoids	Anthocyanins	2.98E+04	2.91E+04	2.82E+04	5.77E+03	1.39E+04	1.41E+04	1.19E+00	3.87E-01	-1.37E+00	up

side												
Quercetin-3-O-(2''-O-arabinosyl)rutinoside	Flavonoids	Flavonols	6.12E+03	8.67E+03	6.10E+03	1.95E+04	2.30E+04	2.14E+04	1.38E+00	3.06E+00	1.61E+00	down
6-Hydroxykaempferol-3-O-rutin-6-O-glucoside	Flavonoids	Flavonols	1.91E+05	2.09E+05	2.20E+05	1.03E+05	8.34E+04	9.38E+04	1.39E+00	4.52E-01	-1.15E+00	up
Catechin-catechin-catechin	Flavonoids	Flavanols	1.01E+06	4.68E+05	1.55E+05	2.17E+04	9.78E+04	2.34E+04	1.21E+00	8.75E-02	-3.51E+00	up
Cyanidin-3-O-rutinoside-5,3'-di-O-glucoside	Flavonoids	Anthocyanins	2.83E+04	2.94E+04	4.58E+04	7.29E+04	6.97E+04	6.73E+04	1.29E+00	2.03E+00	1.02E+00	down

(B)at R2H_vs_Y2H

Compounds	Class I	Class II	Y2H1	Y2H2	Y2H3	R2H1	R2H2	R2H3	VIP	Fold_Change	Log2FC	Type
Apigenin	Flavonoids	Flavonoid	3.31E+04	3.47E+04	3.33E+04	1.11E+04	1.11E+04	8.82E+03	1.49E+00	3.07E-01	-1.70E+00	up
Galangin (3,5,7-Trihydroxyflavone)	Flavonoids	Flavonoid	5.79E+04	7.71E+04	6.36E+04	3.06E+04	2.29E+04	2.65E+04	1.44E+00	4.03E-01	-1.31E+00	up
Afzelechin (3,5,7,4'-Tetrahydroxyflavan)	Flavonoids	Flavanols	2.34E+04	1.27E+04	1.48E+04	6.38E+04	3.33E+04	2.46E+04	1.15E+00	2.39E+00	1.25E+00	down
Dihydrokaempferide	Flavonoids	Flavonols	2.89E+05	1.18E+05	1.77E+05	8.88E+04	1.01E+05	5.21E+04	1.20E+00	4.14E-01	-1.27E+00	up
Chrysin-5-O-glucoside (Toringin)	Flavonoids	Flavonoid	3.60E+05	3.97E+05	2.83E+05	2.53E+05	8.08E+04	7.29E+04	1.20E+00	3.91E-01	-1.36E+00	up
Sophoricoside	Flavonoids	Isoflavones	1.42E+06	1.52E+06	1.44E+06	5.05E+05	5.29E+05	5.37E+05	1.49E+00	3.58E-01	-1.48E+00	up
Genistein-7-O-galactoside	Flavonoids	Isoflavones	1.54E+07	1.77E+07	1.50E+07	5.47E+06	5.32E+06	5.79E+06	1.48E+00	3.45E-01	-1.54E+00	up
Galangin-7-O-glucoside	Flavonoids	Flavonoid	2.03E+06	4.02E+06	1.40E+06	1.02E+06	5.27E+05	5.93E+05	1.26E+00	2.87E-01	-1.80E+00	up
Kaempferol-7-O-rhamnoside	Flavonoids	Flavonols	2.29E+05	2.29E+05	2.28E+05	1.56E+04	1.74E+04	1.20E+04	1.49E+00	6.56E-02	-3.93E+00	up
Kaempferol-3-O-rhamnoside (Afzelin)(Kaempferin)	Flavonoids	Flavonols	2.85E+05	3.02E+05	3.01E+05	1.86E+04	2.17E+04	1.74E+04	1.50E+00	6.49E-02	-3.95E+00	up
Quercetin-3-O-arabinoside (Guaijaverin)	Flavonoids	Flavonols	2.64E+06	2.28E+06	2.62E+06	6.01E+05	1.06E+06	4.69E+05	1.42E+00	2.82E-01	-1.83E+00	up
Morin-3-O-xyloside	Flavonoids	Flavonols	5.98E+06	6.15E+06	6.70E+06	1.97E+06	2.57E+06	1.54E+06	1.46E+00	3.23E-01	-1.63E+00	up
Quercetin-3-O-xyloside (Reynoutrin)	Flavonoids	Flavonols	5.63E+06	5.06E+06	5.67E+06	1.93E+06	2.05E+06	1.42E+06	1.47E+00	3.30E-01	-1.60E+00	up

Avicularin(Quercetin-3-O-α-L-arabinofuranoside)	Flavonoids	Flavonols	5.63E+06	4.63E+06	6.08E+06	1.71E+06	2.15E+06	1.27E+06	1.45E+00	3.14E-01	-1.67E+00	up
Morin-3-O-arabinoside	Flavonoids	Flavonols	4.38E+06	3.75E+06	4.16E+06	1.45E+06	1.71E+06	1.02E+06	1.45E+00	3.40E-01	-1.56E+00	up
Delphinidin-3-O-arabinoside	Flavonoids	Anthocyanins	6.63E+04	7.90E+04	1.10E+05	4.71E+04	1.49E+04	1.57E+04	1.28E+00	3.04E-01	-1.72E+00	up
Dihydrocharcone-4'-O-glucoside	Flavonoids	Chalcones	9.49E+05	9.93E+05	6.54E+05	6.33E+05	2.71E+05	2.22E+05	1.19E+00	4.34E-01	-1.20E+00	up
Acacetin-7-O-glucoside (Tilianin)	Flavonoids	Flavonoid	9.31E+04	1.19E+05	8.02E+04	6.41E+04	1.76E+04	1.87E+04	1.22E+00	3.44E-01	-1.54E+00	up
Acacetin-7-O-galactoside	Flavonoids	Flavonoid	1.23E+05	1.54E+05	1.07E+05	8.04E+04	2.14E+04	2.89E+04	1.23E+00	3.40E-01	-1.56E+00	up
Luteolin-3'-O-glucoside	Flavonoids	Flavonoid	5.19E+06	5.75E+06	4.44E+06	1.66E+07	1.25E+07	1.06E+07	1.43E+00	2.58E+00	1.37E+00	down
Luteolin-8-C-glucoside (Orientin)	Flavonoids	Flavonoid carbonoside	4.66E+05	7.05E+05	4.26E+05	2.15E+05	1.99E+05	1.50E+05	1.40E+00	3.53E-01	-1.50E+00	up
Luteolin-6-C-glucoside (Isoorientin)	Flavonoids	Flavonoid carbonoside	1.77E+05	2.62E+05	1.56E+05	8.12E+04	5.76E+04	4.74E+04	1.39E+00	3.13E-01	-1.68E+00	up
Kaempferol-3-O-galactoside (Trifolin)	Flavonoids	Flavonols	4.41E+05	3.92E+05	5.51E+05	1.55E+05	2.25E+05	1.02E+05	1.38E+00	3.49E-01	-1.52E+00	up
Quercetin-3-O-rhamnoside(Quercitrin)	Flavonoids	Flavonols	3.06E+06	3.01E+06	3.41E+06	3.36E+04	4.06E+04	3.76E+04	1.50E+00	1.18E-02	-6.40E+00	up
Cyanidin-3-O-glucoside (Kuromanin)	Flavonoids	Anthocyanins	4.80E+06	2.92E+06	5.61E+06	9.38E+05	2.07E+06	1.03E+06	1.35E+00	3.03E-01	-1.72E+00	up
Cyanidin-3-O-galactoside	Flavonoids	Anthocyanins	5.35E+06	2.94E+06	5.96E+06	7.42E+05	1.71E+06	7.94E+05	1.38E+00	2.28E-01	-2.14E+00	up
Epicatechin glucoside	Flavonoids	Flavanols	8.04E+06	6.82E+06	5.42E+06	3.99E+06	1.64E+06	1.74E+06	1.30E+00	3.64E-01	-1.46E+00	up
Chrysoeriol-7-O-glucoside	Flavonoids	Flavonoid	4.73E+06	4.66E+06	3.20E+06	3.27E+06	1.13E+06	7.72E+05	1.14E+00	4.11E-01	-1.28E+00	up
5,2'-Dihydroxy-7,8-dimethoxyflavone glycosides	Flavonoids	Flavonoid	1.88E+04	2.99E+04	1.98E+04	2.10E+04	9.00E+00	9.00E+00	1.06E+00	3.07E-01	-1.70E+00	up
Petunidin-3-O-glucoside	Flavonoids	Anthocyanins	6.11E+04	8.74E+04	6.19E+04	9.00E+00	9.00E+00	9.00E+00	1.49E+00	1.28E-04	-1.29E+01	up
Kaempferol-3-O-(2"-acetyl)glucoside	Flavonoids	Flavonols	1.69E+04	1.37E+04	1.92E+04	5.24E+03	8.59E+03	6.18E+03	1.41E+00	4.02E-01	-1.31E+00	up
Kaempferol-3-O-(6"-acetyl)glucoside	Flavonoids	Flavonols	3.71E+05	3.28E+05	5.11E+05	7.49E+04	9.47E+04	5.50E+04	1.46E+00	1.86E-01	-2.43E+00	up
Quercetin-3-O-(6"-acetyl)galactoside	Flavonoids	Flavonols	3.70E+04	2.68E+04	3.19E+04	5.27E+03	4.52E+03	6.95E+03	1.47E+00	1.75E-01	-2.52E+00	up
Kaempferol-3-O-(6"-malonyl)glucoside	Flavonoids	Flavonols	1.05E+06	8.72E+05	1.16E+06	1.73E+05	2.20E+05	1.61E+05	1.49E+00	1.80E-01	-2.47E+00	up
Kaempferol-3-O-(6"-malonyl)galactosi	Flavonoids	Flavonols	1.02E+06	9.09E+05	9.93E+05	1.86E+05	2.14E+05	1.92E+05	1.49E+00	2.03E-01	-2.30E+00	up

de												
Quercetin-7-O-(6"-malonyl)glucoside	Flavonoids	Flavonols	2.26E+05	1.84E+05	2.14E+05	3.77E+04	5.21E+04	5.87E+04	1.47E+00	2.38E-01	-2.07E+00	up
Quercetin-3-O-(6"-malonyl)galactoside	Flavonoids	Flavonols	8.24E+04	6.56E+04	9.00E+04	7.43E+03	1.72E+04	1.57E+04	1.43E+00	1.69E-01	-2.56E+00	up
Genistein-7-O-galactoside-rhamnose	Flavonoids	Isoflavones	1.23E+05	1.20E+05	1.36E+05	1.73E+04	1.82E+04	3.32E+04	1.45E+00	1.82E-01	-2.46E+00	up
Kaempferol-3,7-O-dirhamnoside (Kaempferitrin)	Flavonoids	Flavonols	2.05E+06	2.47E+06	1.36E+06	1.58E+06	2.78E+05	2.21E+05	1.10E+00	3.54E-01	-1.50E+00	up
Apigenin-7-O-rutinoside (Isorhoifolin)	Flavonoids	Flavonoid	1.28E+05	1.27E+05	1.59E+05	2.27E+04	2.05E+04	5.29E+03	1.40E+00	1.17E-01	-3.09E+00	up
Pelargonidin-3-O-rutinoside	Flavonoids	Anthocyanins	5.00E+06	6.57E+06	3.99E+06	4.99E+06	9.87E+05	8.56E+05	1.04E+00	4.39E-01	-1.19E+00	up
Cyanidin-3-O-sambubioside [Cyanidin-3-O-(2"-O-xylosyl)glucoside]	Flavonoids	Anthocyanins	9.22E+04	1.25E+05	8.09E+04	9.56E+04	1.06E+04	1.62E+04	1.04E+00	4.11E-01	-1.28E+00	up
Luteolin-7-O-(6"-caffeoyl)rhamnoside	Flavonoids	Flavonoid	1.39E+05	6.28E+04	1.16E+05	1.67E+04	2.68E+04	1.53E+04	1.42E+00	1.85E-01	-2.43E+00	up
Kaempferol-3-O-(6"-p-Coumaroyl)glucoside (Tiliroside)	Flavonoids	Flavonols	3.87E+05	1.97E+05	3.01E+05	5.53E+04	9.18E+04	4.34E+04	1.41E+00	2.15E-01	-2.22E+00	up
Kaempferol-3-O-(6"-p-Coumaroyl)galactoside	Flavonoids	Flavonoid	1.04E+05	4.41E+04	6.87E+04	9.00E+00	9.00E+00	9.00E+00	1.49E+00	1.25E-04	-1.30E+01	up
Kaempferol-3-O-(2"-p-Coumaroyl)galactoside	Flavonoids	Flavonoid	1.05E+05	6.40E+04	7.07E+04	9.00E+00	9.00E+00	9.00E+00	1.49E+00	1.12E-04	-1.31E+01	up
Isovitexin-7-O-glucoside(Saponarin)	Flavonoids	Flavonoid carbonoside	4.95E+05	4.33E+05	4.93E+05	2.29E+05	1.95E+05	2.75E+05	1.42E+00	4.92E-01	-1.02E+00	up
Kaempferol-3-O-rhamnosyl(1→2)glucoside	Flavonoids	Flavonoid	2.99E+04	1.07E+04	2.58E+04	4.13E+04	6.79E+04	3.57E+04	1.09E+00	2.18E+00	1.12E+00	down
Poncirin (Isosakuranetin-7-O-neohesperidoside)	Flavonoids	Dihydroflavone	4.43E+05	2.52E+05	3.15E+05	6.36E+04	7.83E+04	6.37E+04	1.46E+00	2.04E-01	-2.30E+00	up
Kaempferol-3-O-(6"-galloyl)galactoside	Flavonoids	Flavonoid	2.60E+04	1.90E+04	3.66E+04	2.02E+04	1.06E+04	4.90E+03	1.11E+00	4.37E-01	-1.20E+00	up

Quercetin-3-O-(6''-p-Coumaroyl)galactoside	Flavonoids	Flavonols	1.15E+05	7.53E+04	1.06E+05	3.56E+04	2.11E+04	2.48E+04	1.43E+00	2.75E-01	-1.86E+00	up
Quercetin-3-O-(6''-p-Coumaroyl)glucoside	Flavonoids	Flavonols	1.33E+05	7.58E+04	9.15E+04	2.47E+04	2.35E+04	1.60E+04	1.45E+00	2.14E-01	-2.23E+00	up
Luteolin-6,8-di-C-glucoside	Flavonoids	Flavonoid carbonoside	1.66E+05	2.03E+05	1.95E+05	8.48E+04	3.86E+04	9.26E+04	1.29E+00	3.83E-01	-1.39E+00	up
Luteolin-6-C-glucoside-7-O-glucoside	Flavonoids	Flavonoid carbonoside	3.18E+04	3.72E+04	4.56E+04	2.04E+04	9.00E+00	9.00E+00	1.12E+00	1.78E-01	-2.49E+00	up
Kaempferol-6,8-di-C-glucoside	Flavonoids	Flavonoid	9.93E+04	1.00E+05	9.95E+04	4.51E+04	4.25E+04	5.89E+04	1.43E+00	4.90E-01	-1.03E+00	up
Quercetin-3-O-(6''-galloyl)galactoside	Flavonoids	Flavonols	1.36E+04	8.42E+03	1.33E+04	9.00E+00	9.00E+00	9.00E+00	1.50E+00	7.66E-04	-1.04E+01	up
Isorhamnetin-3-O-neohesperidoside	Flavonoids	Flavonols	1.85E+05	1.43E+05	2.03E+05	6.49E+04	1.04E+05	9.17E+04	1.36E+00	4.91E-01	-1.03E+00	up
Quercetin-3-O-sophoroside (Baimaside)	Flavonoids	Flavonols	1.39E+06	8.04E+05	1.35E+06	4.84E+05	6.94E+05	4.70E+05	1.31E+00	4.66E-01	-1.10E+00	up
Hesperetin-6-C-glucoside-7-O-glucoside	Flavonoids	Flavonoid carbonoside	1.06E+06	8.99E+05	9.19E+05	4.08E+05	4.93E+05	3.96E+05	1.47E+00	4.50E-01	-1.15E+00	up
Quercetin-3-O-(2''-O-arabinosyl)rutinoside	Flavonoids	Flavonols	7.61E+03	1.22E+04	4.57E+03	1.98E+04	2.70E+04	3.02E+04	1.35E+00	3.16E+00	1.66E+00	down
6-Hydroxykaempferol-3-O-rutin-6-O-glucoside	Flavonoids	Flavonols	2.46E+05	1.81E+05	2.34E+05	6.77E+04	1.00E+05	8.54E+04	1.44E+00	3.83E-01	-1.39E+00	up
Cyanidin-3-O-rutinoside-5,3'-di-O-glucoside	Flavonoids	Anthocyanins	3.34E+04	3.58E+04	3.05E+04	5.48E+04	8.83E+04	6.57E+04	1.38E+00	2.09E+00	1.07E+00	down

(C)at R6H_vs_Y6H

Compounds	Class I	Class II	Y6H1	Y6H2	Y6H3	R6H1	R6H2	R6H3	VIP	Fold_Change	Log2FC	Type
Apigenin	Flavonoids	Flavonoid	1.40E+04	1.63E+04	4.11E+04	7.89E+03	1.11E+04	8.63E+03	1.10E+00	3.87E-01	-1.37E+00	up
Galangin (3,5,7-Trihydroxyflavone)	Flavonoids	Flavonoid	3.24E+04	3.86E+04	7.17E+04	1.24E+04	1.80E+04	1.52E+04	1.30E+00	3.19E-01	-1.65E+00	up
Phloretin	Flavonoids	Chalcones	1.60E+04	1.36E+04	1.70E+04	3.04E+03	6.45E+03	3.10E+03	1.35E+00	2.70E-01	-1.89E+00	up

Catechin	Flavonoids	Flavanols	1.17E+06	1.50E+06	1.74E+06	3.68E+05	1.06E+06	5.96E+05	1.15E+00	4.58E-01	-1.13E+00	up
Epicatechin	Flavonoids	Flavanols	1.77E+06	2.13E+06	3.37E+06	5.60E+05	1.83E+06	9.58E+05	1.06E+00	4.60E-01	-1.12E+00	up
Dihydroquercetin(Taxifolin)	Flavonoids	Dihydroflavonol	3.45E+05	2.63E+05	2.79E+05	1.46E+04	8.11E+04	2.10E+04	1.32E+00	1.32E-01	-2.92E+00	up
5,7,3',4',5'-Pentahydroxydihydroflavone	Flavonoids	Dihydroflavone	4.96E+04	1.15E+05	1.00E+05	1.50E+04	6.18E+04	2.03E+04	1.09E+00	3.66E-01	-1.45E+00	up
Nepetin (5,7,3',4'-Tetrahydroxy-6-methoxyflavone)	Flavonoids	Flavonoid	4.74E+03	5.01E+03	9.74E+03	1.91E+03	3.96E+03	3.31E+03	1.09E+00	4.71E-01	-1.08E+00	up
Chrysin-5-O-glucoside (Toringin)	Flavonoids	Flavonoid	1.36E+05	1.86E+05	3.20E+05	5.37E+04	1.33E+05	9.11E+04	1.10E+00	4.32E-01	-1.21E+00	up
Sophoricoside	Flavonoids	Isoflavones	7.18E+05	9.86E+05	1.38E+06	3.60E+05	4.69E+05	5.47E+05	1.26E+00	4.46E-01	-1.17E+00	up
Genistein-7-O-galactoside	Flavonoids	Isoflavones	7.84E+06	1.15E+07	1.50E+07	3.96E+06	5.27E+06	5.47E+06	1.29E+00	4.28E-01	-1.23E+00	up
Galangin-7-O-glucoside	Flavonoids	Flavonoid	8.50E+05	1.71E+06	2.03E+06	6.24E+05	7.35E+05	6.15E+05	1.19E+00	4.30E-01	-1.22E+00	up
Kaempferol-7-O-rhamnoside	Flavonoids	Flavonols	1.54E+05	1.90E+05	2.88E+05	8.29E+03	1.12E+04	1.32E+04	1.44E+00	5.17E-02	-4.27E+00	up
Kaempferol-3-O-rhamnoside (Afzelin)(Kaempferin)	Flavonoids	Flavonols	2.12E+05	2.38E+05	3.89E+05	1.11E+04	1.53E+04	1.76E+04	1.44E+00	5.24E-02	-4.25E+00	up
Quercetin-3-O-arabinoside (Guaijaverin)	Flavonoids	Flavonols	1.61E+06	2.02E+06	2.11E+06	3.99E+05	4.53E+05	7.62E+05	1.39E+00	2.81E-01	-1.83E+00	up
Morin-3-O-xyloside	Flavonoids	Flavonols	4.45E+06	4.42E+06	5.75E+06	1.19E+06	1.38E+06	2.21E+06	1.37E+00	3.27E-01	-1.61E+00	up
Quercetin-3-O-xyloside (Reynoutrin)	Flavonoids	Flavonols	4.55E+06	4.07E+06	4.46E+06	8.58E+05	1.11E+06	1.94E+06	1.37E+00	2.99E-01	-1.74E+00	up
Avicularin(Quercetin-3-O-α-L-arabinofuranoside)	Flavonoids	Flavonols	3.79E+06	3.76E+06	4.69E+06	8.46E+05	1.08E+06	1.90E+06	1.35E+00	3.13E-01	-1.68E+00	up
Morin-3-O-arabinoside	Flavonoids	Flavonols	2.77E+06	3.28E+06	3.53E+06	7.43E+05	9.86E+05	1.73E+06	1.32E+00	3.62E-01	-1.47E+00	up
Butin-7-O-glucoside	Flavonoids	Flavonoid	9.91E+05	1.31E+06	1.24E+06	1.53E+05	4.76E+05	2.66E+05	1.32E+00	2.53E-01	-1.98E+00	up
Naringenin-4'-O-glucoside	Flavonoids	Flavonoid	1.09E+06	1.38E+06	1.32E+06	1.55E+05	4.74E+05	2.94E+05	1.33E+00	2.44E-01	-2.04E+00	up
Isosalipurposide (Phlorizin Chalcone)	Flavonoids	Chalcones	7.53E+05	9.56E+05	8.59E+05	1.01E+05	3.15E+05	1.93E+05	1.33E+00	2.37E-01	-2.08E+00	up

Dihydrocharcone-4'-O-glucoside	Flavonoids	Chalcones	8.02E+05	1.38E+06	1.44E+06	1.89E+05	4.44E+05	2.70E+05	1.33E+00	2.49E-01	-2.00E+00	up
Phloretin-2'-O-glucoside (Phlorizin)	Flavonoids	Chalcones	4.71E+05	7.30E+05	9.39E+05	7.38E+04	5.80E+05	1.83E+05	1.01E+00	3.91E-01	-1.36E+00	up
Acacetin-7-O-glucoside (Tilianin)	Flavonoids	Flavonoid	3.39E+04	4.76E+04	8.41E+04	9.15E+03	3.76E+04	1.86E+04	1.04E+00	3.95E-01	-1.34E+00	up
Acacetin-7-O-galactoside	Flavonoids	Flavonoid	4.36E+04	5.88E+04	1.13E+05	8.73E+03	5.17E+04	1.33E+04	1.05E+00	3.42E-01	-1.55E+00	up
Luteolin-3'-O-glucoside	Flavonoids	Flavonoid	2.52E+06	3.40E+06	4.84E+06	1.01E+07	1.29E+07	1.11E+07	1.38E+00	3.17E+00	1.66E+00	down
Luteolin-8-C-glucoside (Orientin)	Flavonoids	Flavonoid carbonoside	2.32E+05	4.69E+05	5.30E+05	1.57E+05	1.96E+05	1.47E+05	1.22E+00	4.06E-01	-1.30E+00	up
Luteolin-6-C-glucoside (Isoorientin)	Flavonoids	Flavonoid carbonoside	9.30E+04	1.20E+05	1.98E+05	5.63E+04	8.17E+04	4.36E+04	1.16E+00	4.42E-01	-1.18E+00	up
Kaempferol-3-O-galactoside (Trifolin)	Flavonoids	Flavonols	3.59E+05	3.93E+05	4.00E+05	7.60E+04	1.05E+05	1.82E+05	1.35E+00	3.15E-01	-1.67E+00	up
Quercetin-3-O-rhamnoside(Quercitrin)	Flavonoids	Flavonols	1.74E+06	2.01E+06	2.73E+06	1.60E+04	2.52E+04	3.91E+04	1.44E+00	1.24E-02	-6.33E+00	up
Cyanidin-3-O-glucoside (Kuromanin)	Flavonoids	Anthocyanins	3.00E+06	5.38E+06	3.04E+06	9.88E+05	7.83E+05	1.36E+06	1.37E+00	2.74E-01	-1.87E+00	up
Cyanidin-3-O-galactoside	Flavonoids	Anthocyanins	3.14E+06	4.50E+06	2.83E+06	9.04E+05	8.77E+05	1.27E+06	1.41E+00	2.91E-01	-1.78E+00	up
Aromadendrin-7-O-glucoside	Flavonoids	Flavonoid	8.23E+06	9.09E+06	5.76E+06	3.25E+06	2.23E+06	3.83E+06	1.34E+00	4.03E-01	-1.31E+00	up
Epicatechin glucoside	Flavonoids	Flavanols	3.98E+06	4.93E+06	6.67E+06	1.16E+06	2.97E+06	1.88E+06	1.23E+00	3.86E-01	-1.37E+00	up
Gallate catechin gallate	Flavonoids	Flavanols	4.59E+05	4.99E+05	2.27E+05	1.32E+04	1.10E+05	5.28E+04	1.24E+00	1.49E-01	-2.75E+00	up
Epigallocatechin-3-gallate	Flavonoids	Flavanols	4.44E+05	5.17E+05	2.44E+05	1.35E+04	1.20E+05	5.91E+04	1.23E+00	1.60E-01	-2.65E+00	up
Chrysoeriol-7-O-glucoside	Flavonoids	Flavonoid	2.15E+06	2.15E+06	3.36E+06	5.87E+05	1.74E+06	1.27E+06	1.10E+00	4.70E-01	-1.09E+00	up
6-Hydroxykaempferol-7-O-glucoside	Flavonoids	Flavonols	1.06E+06	1.65E+06	1.51E+06	6.21E+05	3.61E+05	9.99E+05	1.16E+00	4.69E-01	-1.09E+00	up
5,2'-Dihydroxy-7,8-dimethoxyflavone glycosides	Flavonoids	Flavonoid	5.79E+03	1.02E+04	1.09E+04	9.00E+00	9.44E+03	9.00E+00	1.00E+00	3.52E-01	-1.51E+00	up
Petunidin-3-O-glucoside	Flavonoids	Anthocyanins	6.18E+04	5.89E+04	5.07E+04	9.00E+00	9.00E+00	9.00E+00	1.45E+00	1.57E-04	-1.26E+01	up
Kaempferol-3-O-(6"-acetyl)glucoside	Flavonoids	Flavonols	3.47E+05	3.50E+05	2.85E+05	9.48E+04	4.83E+04	9.83E+04	1.39E+00	2.46E-01	-2.02E+00	up
Quercetin-3-O-(6"-acetyl)glucoside	Flavonoids	Flavonols	9.17E+03	1.87E+04	2.38E+04	9.00E+00	7.27E+03	9.00E+00	1.09E+00	1.41E-01	-2.83E+00	up
Quercetin-3-O-(6"-acetyl)galactoside	Flavonoids	Flavonols	2.95E+04	2.40E+04	2.92E+04	4.94E+03	6.33E+03	7.47E+03	1.43E+00	2.27E-01	-2.14E+00	up
Kaempferol-3-O-(6"-malonyl)glucosid	Flavonoids	Flavonols	8.35E+05	7.30E+05	7.06E+05	2.23E+05	1.55E+05	2.32E+05	1.43E+00	2.68E-01	-1.90E+00	up

e												
Kaempferol-3-O-(6"-malonyl)galactoside	Flavonoids	Flavonols	7.79E+05	6.85E+05	7.24E+05	2.26E+05	1.56E+05	2.64E+05	1.41E+00	2.96E-01	-1.76E+00	up
Robustaflavone	Flavonoids	Biflavones	6.14E+03	4.52E+03	9.33E+03	2.04E+03	2.13E+03	3.97E+03	1.21E+00	4.07E-01	-1.30E+00	up
Quercetin-7-O-(6"-malonyl)glucoside	Flavonoids	Flavonols	1.19E+05	1.18E+05	4.36E+05	3.76E+04	4.06E+04	4.59E+04	1.24E+00	1.84E-01	-2.44E+00	up
Quercetin-3-O-(6"-malonyl)galactoside	Flavonoids	Flavonols	5.68E+04	6.44E+04	6.60E+04	1.82E+04	1.15E+04	2.24E+04	1.40E+00	2.79E-01	-1.84E+00	up
Genistein-7-O-galactoside-rhamnose	Flavonoids	Isoflavones	8.35E+04	1.31E+05	1.43E+05	1.99E+04	1.26E+04	1.28E+04	1.42E+00	1.27E-01	-2.98E+00	up
Kaempferol-3,7-O-dirhamnoside (Kaempferitrin)	Flavonoids	Flavonols	9.11E+05	1.19E+06	1.89E+06	8.51E+04	5.94E+05	2.15E+05	1.19E+00	2.24E-01	-2.16E+00	up
Apigenin-7-O-rutinoside (Isorhoifolin)	Flavonoids	Flavonoid	8.95E+04	1.32E+05	1.27E+05	2.62E+04	3.25E+04	3.52E+04	1.42E+00	2.69E-01	-1.89E+00	up
Pelargonidin-3-O-rutinoside	Flavonoids	Anthocyanins	2.35E+06	2.96E+06	5.77E+06	4.76E+05	2.15E+06	1.05E+06	1.10E+00	3.32E-01	-1.59E+00	up
Quercetin-3-O-rhamnosyl(1→2)arabinoside	Flavonoids	Flavonols	1.88E+04	2.49E+04	3.48E+04	9.22E+03	8.74E+03	5.27E+03	1.33E+00	2.96E-01	-1.76E+00	up
Cyanidin-3-O-sambubioside [Cyanidin-3-O-(2"-O-xylosyl)glucoside]	Flavonoids	Anthocyanins	4.33E+04	5.18E+04	1.04E+05	7.66E+03	4.40E+04	1.65E+04	1.06E+00	3.43E-01	-1.55E+00	up
Luteolin-7-O-(6"-caffeoyl)rhamnoside	Flavonoids	Flavonoid	4.29E+04	6.11E+04	1.36E+05	8.81E+03	1.58E+04	2.98E+04	1.22E+00	2.27E-01	-2.14E+00	up
Kaempferol-3-O-(6"-p-Coumaroyl)glucoside (Tiliroside)	Flavonoids	Flavonols	1.28E+05	2.09E+05	3.58E+05	2.64E+04	4.17E+04	8.40E+04	1.26E+00	2.19E-01	-2.19E+00	up
Kaempferol-3-O-(6"-p-Coumaroyl)galactoside	Flavonoids	Flavonoid	2.04E+04	5.07E+04	9.35E+04	9.00E+00	9.00E+00	9.00E+00	1.45E+00	1.64E-04	-1.26E+01	up
Kaempferol-3-O-(2"-p-Coumaroyl)galactoside	Flavonoids	Flavonoid	2.41E+04	3.87E+04	9.04E+04	9.00E+00	9.00E+00	9.00E+00	1.45E+00	1.76E-04	-1.25E+01	up
Poncirin (Isosakuranetin-7-O-neohesperidoside)	Flavonoids	Dihydroflavone	1.34E+05	2.26E+05	4.22E+05	2.76E+04	4.38E+04	8.89E+04	1.26E+00	2.05E-01	-2.29E+00	up

Pelargonidin-3,5-O-diglucoside	Flavonoids	Anthocyanins	8.13E+04	6.23E+04	1.40E+05	2.38E+04	4.77E+04	5.21E+04	1.11E+00	4.36E-01	-1.20E+00	up
Kaempferol-3-O-(2''-galloyl)galactoside	Flavonoids	Flavonoid	6.11E+03	8.73E+03	5.54E+05	9.00E+00	9.00E+00	1.36E+04	1.04E+00	2.40E-02	-5.38E+00	up
Quercetin-3-O-(6''-p-Coumaroyl)galactoside	Flavonoids	Flavonols	3.29E+04	6.28E+04	1.74E+05	1.29E+04	1.58E+04	3.08E+04	1.13E+00	2.21E-01	-2.18E+00	up
Quercetin-3-O-(6''-p-Coumaroyl)glucoside	Flavonoids	Flavonols	3.34E+04	5.91E+04	1.67E+05	1.16E+04	1.48E+04	2.79E+04	1.16E+00	2.10E-01	-2.25E+00	up
Kaempferol-6,8-di-C-glucoside	Flavonoids	Flavonoid	5.14E+04	5.56E+04	9.53E+04	3.54E+04	3.02E+04	2.65E+04	1.26E+00	4.55E-01	-1.13E+00	up
Cyanidin-3-O-(6''-O-caffeoyl)glucoside	Flavonoids	Anthocyanins	2.49E+04	1.70E+04	4.00E+04	3.68E+04	1.04E+05	6.21E+04	1.11E+00	2.48E+00	1.31E+00	down
Quercetin-3-O-(6''-galloyl)galactoside	Flavonoids	Flavonols	5.76E+03	3.11E+03	4.60E+05	9.00E+00	9.00E+00	9.00E+00	1.34E+00	5.76E-05	-1.41E+01	up
Peonidin-3,5-O-diglucoside	Flavonoids	Anthocyanins	3.58E+04	3.34E+04	3.65E+04	1.03E+05	6.81E+04	7.11E+04	1.39E+00	2.29E+00	1.20E+00	down
6-Hydroxykaempferol-3,6-O-Diglucoside	Flavonoids	Flavonols	4.84E+05	4.36E+05	3.08E+05	8.73E+05	9.98E+05	9.82E+05	1.37E+00	2.32E+00	1.22E+00	down
Apigenin-7-O-rutinoside-4'-O-rhamnoside	Flavonoids	Flavonoid	4.28E+04	6.38E+04	7.67E+04	1.34E+05	1.63E+05	8.08E+04	1.17E+00	2.06E+00	1.04E+00	down
Vitexin-7-O-(6''-feruloyl)glucoside	Flavonoids	Flavonoid carbonoside	4.29E+04	3.65E+04	4.60E+04	2.34E+04	1.51E+04	1.92E+04	1.36E+00	4.60E-01	-1.12E+00	up
6-Hydroxykaempferol-3-O-rutin-6-O-glucoside	Flavonoids	Flavonols	2.06E+05	2.10E+05	2.31E+05	6.26E+04	7.94E+04	1.02E+05	1.40E+00	3.76E-01	-1.41E+00	up
Catechin-catechin-catechin	Flavonoids	Flavanols	2.57E+05	3.67E+05	1.07E+06	2.73E+04	1.43E+05	7.26E+04	1.21E+00	1.43E-01	-2.80E+00	up

(D)at R20H_vs_Y20H

Compounds	Class I	Class II	Y20H1	Y20H2	Y20H3	R20H1	R20H2	R20H3	VIP	Fold_Change	Log2FC	Type
Apigenin	Flavonoids	Flavonoid	4.40E+04	2.75E+04	3.93E+04	1.08E+04	6.89E+03	7.78E+03	1.35E+00	2.30E-01	-2.12E+00	up
Galangin (3,5,7-Trihydroxyflavone)	Flavonoids	Flavonoid	7.97E+04	6.67E+04	8.70E+04	2.83E+04	2.16E+04	1.90E+04	1.35E+00	2.95E-01	-1.76E+00	up
Naringenin	Flavonoids	Dihydroflavon	2.75E+04	3.37E+04	3.89E+04	1.77E+04	1.07E+04	1.58E+04	1.27E+00	4.41E-01	-1.18E+00	up

(5,7,4'-Trihydroxyflavanone)		e										
Naringenin chalcone	Flavonoids	Chalcones	3.06E+04	3.26E+04	3.42E+04	1.43E+04	1.53E+04	1.55E+04	1.37E+00	4.62E-01	-1.11E+00	up
Pinobanksin	Flavonoids	Dihydroflavonol	2.49E+04	3.23E+04	3.92E+04	1.58E+04	1.21E+04	1.41E+04	1.29E+00	4.36E-01	-1.20E+00	up
Phloretin	Flavonoids	Chalcones	2.33E+04	2.33E+04	2.75E+04	3.72E+03	1.77E+03	6.07E+03	1.31E+00	1.56E-01	-2.68E+00	up
2'-Hydroxygenistein	Flavonoids	Isoflavones	1.54E+04	1.59E+04	1.72E+04	4.48E+03	3.74E+03	7.07E+03	1.32E+00	3.15E-01	-1.67E+00	up
Isoscutellarein	Flavonoids	Flavonoid	1.18E+04	1.26E+04	1.63E+04	4.02E+03	4.34E+03	5.39E+03	1.34E+00	3.38E-01	-1.56E+00	up
Luteolin (5,7,3',4'-Tetrahydroxyflavone)	Flavonoids	Flavonoid	6.94E+04	8.86E+04	8.76E+04	2.62E+04	1.38E+04	2.31E+04	1.33E+00	2.57E-01	-1.96E+00	up
Dihydrokaempferol	Flavonoids	Dihydroflavonol	2.83E+04	3.31E+04	3.81E+04	1.24E+04	8.69E+03	1.66E+04	1.28E+00	3.79E-01	-1.40E+00	up
Catechin	Flavonoids	Flavanols	2.30E+06	2.35E+06	2.11E+06	3.83E+05	2.57E+05	7.39E+05	1.31E+00	2.04E-01	-2.29E+00	up
Epicatechin	Flavonoids	Flavanols	3.44E+06	4.13E+06	3.38E+06	6.60E+05	4.24E+05	1.17E+06	1.31E+00	2.06E-01	-2.28E+00	up
5,7,2'-Trhiyroxy-8-methoxyflavone	Flavonoids	Flavonoid	3.56E+05	4.38E+05	4.65E+05	1.76E+05	1.11E+05	1.51E+05	1.33E+00	3.48E-01	-1.52E+00	up
Hispidulin (5,7,4'-Trihydroxy-6-methoxyflavone)	Flavonoids	Flavonoid	3.35E+05	3.55E+05	3.85E+05	1.15E+05	7.41E+04	9.66E+04	1.36E+00	2.66E-01	-1.91E+00	up
6,7,8-Tetrahydroxy-5-methoxyflavone	Flavonoids	Flavonoid	3.34E+05	3.57E+05	3.92E+05	1.12E+05	7.37E+04	9.88E+04	1.36E+00	2.63E-01	-1.93E+00	up
Diosmetin (5,7,3'-Trihydroxy-4'-methoxyflavone)	Flavonoids	Flavonoid	4.06E+05	4.72E+05	5.04E+05	1.94E+05	1.22E+05	1.69E+05	1.34E+00	3.51E-01	-1.51E+00	up
Dihydroquercetin(Taxifolin)	Flavonoids	Dihydroflavonol	6.12E+05	3.77E+05	4.45E+05	4.33E+04	1.94E+04	1.67E+05	1.20E+00	1.61E-01	-2.64E+00	up
5,7,3',4',5'-Pentahydroxydihydroflavone	Flavonoids	Dihydroflavone	1.25E+05	9.08E+04	1.24E+05	1.60E+04	1.69E+04	5.43E+04	1.22E+00	2.57E-01	-1.96E+00	up
Nepetin	Flavonoids	Flavonoid	1.43E+04	1.06E+04	7.79E+03	5.79E+03	4.37E+03	3.25E+03	1.23E+00	4.10E-01	-1.29E+00	up

(5,7,3',4'-Tetrahydroxy-6-methoxyflavone)												
Chrysin-5-O-glucoside (Toringin)	Flavonoids	Flavonoid	3.70E+05	5.24E+05	4.13E+05	8.61E+04	4.94E+04	7.96E+04	1.36E+00	1.65E-01	-2.60E+00	up
Puerarin	Flavonoids	Isoflavones	7.07E+04	7.90E+04	6.45E+04	1.50E+04	9.32E+03	6.07E+04	1.04E+00	3.97E-01	-1.33E+00	up
Pinocembrin-7-O-glucoside (Pinocembroside)	Flavonoids	Flavonoid	5.54E+04	8.48E+04	6.41E+04	5.38E+03	3.17E+03	1.90E+04	1.25E+00	1.35E-01	-2.89E+00	up
Sophoricoside	Flavonoids	Isoflavones	1.53E+06	1.50E+06	1.43E+06	5.43E+05	4.67E+05	5.34E+05	1.38E+00	3.47E-01	-1.53E+00	up
Genistein-8-C-glucoside	Flavonoids	Isoflavones	1.31E+07	1.13E+07	1.06E+07	4.33E+06	3.94E+06	6.45E+06	1.31E+00	4.22E-01	-1.25E+00	up
Genistein-7-O-galactoside	Flavonoids	Isoflavones	1.72E+07	1.65E+07	1.57E+07	6.00E+06	4.94E+06	5.76E+06	1.38E+00	3.39E-01	-1.56E+00	up
Apigenin-5-O-glucoside	Flavonoids	Flavonoid	4.57E+06	3.36E+06	3.31E+06	1.48E+06	1.54E+06	2.29E+06	1.27E+00	4.73E-01	-1.08E+00	up
Galangin-7-O-glucoside	Flavonoids	Flavonoid	1.92E+06	3.03E+06	2.14E+06	4.97E+05	5.36E+05	6.50E+05	1.35E+00	2.38E-01	-2.07E+00	up
Kaempferol-7-O-rhamnoside	Flavonoids	Flavonols	2.73E+05	3.31E+05	2.60E+05	1.11E+04	1.34E+04	1.06E+04	1.38E+00	4.06E-02	-4.62E+00	up
Kaempferol-3-O-rhamnoside (Afzelin)(Kaempferin)	Flavonoids	Flavonols	3.58E+05	3.95E+05	2.94E+05	1.70E+04	1.15E+04	2.06E+04	1.38E+00	4.69E-02	-4.41E+00	up
Genistein-7-O-Glucoside (Genistin)	Flavonoids	Isoflavones	3.58E+04	4.62E+04	4.01E+04	1.60E+04	1.68E+04	1.54E+04	1.36E+00	3.95E-01	-1.34E+00	up
Quercetin-3-O-arabinoside (Guaijaverin)	Flavonoids	Flavonols	1.71E+06	2.45E+06	1.71E+06	5.10E+05	4.41E+05	4.47E+05	1.36E+00	2.39E-01	-2.07E+00	up
Morin-3-O-xyloside	Flavonoids	Flavonols	4.86E+06	7.78E+06	5.95E+06	1.76E+06	1.72E+06	1.65E+06	1.35E+00	2.77E-01	-1.85E+00	up
Quercetin-3-O-xyloside (Reynoutrin)	Flavonoids	Flavonols	3.88E+06	6.27E+06	4.60E+06	1.45E+06	1.42E+06	1.54E+06	1.34E+00	2.99E-01	-1.74E+00	up
Avicularin(Quercetin-3-O-α-L-arabinofuranoside)	Flavonoids	Flavonols	3.90E+06	5.91E+06	4.66E+06	1.42E+06	1.46E+06	1.44E+06	1.35E+00	2.99E-01	-1.74E+00	up
Morin-3-O-arabinoside	Flavonoids	Flavonols	3.12E+06	4.53E+06	3.60E+06	1.35E+06	1.14E+06	1.19E+06	1.35E+00	3.27E-01	-1.61E+00	up
Butin-7-O-glucoside	Flavonoids	Flavonoid	1.77E+06	1.88E+06	1.61E+06	2.03E+05	1.24E+05	3.20E+05	1.35E+00	1.23E-01	-3.02E+00	up
Naringenin-4'-O-glucoside	Flavonoids	Flavonoid	1.83E+06	1.67E+06	1.52E+06	2.07E+05	1.24E+05	3.45E+05	1.34E+00	1.35E-01	-2.89E+00	up
Isosalipurposide (Phlorizin Chalcone)	Flavonoids	Chalcones	1.19E+06	1.28E+06	1.00E+06	1.27E+05	7.99E+04	2.20E+05	1.34E+00	1.23E-01	-3.03E+00	up
Naringenin-7-O-glucoside (Prunin)	Flavonoids	Dihydroflavon	1.03E+06	1.48E+06	1.19E+06	1.92E+05	2.06E+05	3.76E+05	1.33E+00	2.09E-01	-2.26E+00	up

		e										
Delphinidin-3-O-arabinoside	Flavonoids	Anthocyanins	1.02E+05	1.09E+05	1.26E+05	1.39E+04	1.27E+04	3.53E+04	1.30E+00	1.84E-01	-2.44E+00	up
Dihydrocharcone-4'-O-glucoside	Flavonoids	Chalcones	1.23E+06	1.43E+06	1.21E+06	2.47E+05	1.85E+05	3.37E+05	1.36E+00	1.99E-01	-2.33E+00	up
Phloretin-2'-O-glucoside (Phlorizin)	Flavonoids	Chalcones	1.20E+06	1.25E+06	9.36E+05	9.34E+04	5.45E+04	4.17E+05	1.21E+00	1.66E-01	-2.59E+00	up
Phloretin-4'-O-glucoside (Trilobatin)	Flavonoids	Chalcones	8.33E+06	1.12E+07	7.35E+06	6.81E+05	4.96E+05	2.84E+06	1.24E+00	1.49E-01	-2.74E+00	up
Catechin gallate	Flavonoids	Flavanols	6.91E+06	1.09E+07	7.27E+06	4.10E+05	2.09E+05	3.88E+06	1.13E+00	1.79E-01	-2.48E+00	up
7-O-Galloyltricetiflavan	Flavonoids	Flavanols	3.96E+06	7.15E+06	4.83E+06	2.73E+05	1.51E+05	2.01E+06	1.17E+00	1.52E-01	-2.71E+00	up
Epicatechin gallate	Flavonoids	Flavanols	6.67E+06	9.80E+06	6.80E+06	3.91E+05	2.38E+05	3.04E+06	1.17E+00	1.58E-01	-2.66E+00	up
Acacetin-7-O-glucoside (Tilianin)	Flavonoids	Flavonoid	9.11E+04	1.22E+05	1.16E+05	1.74E+04	8.31E+03	2.87E+04	1.30E+00	1.65E-01	-2.60E+00	up
Acacetin-7-O-galactoside	Flavonoids	Flavonoid	1.24E+05	1.50E+05	1.73E+05	1.81E+04	1.90E+04	4.19E+04	1.32E+00	1.77E-01	-2.50E+00	up
Isorhamnetin-3-O-arabinoside	Flavonoids	Flavonols	6.56E+04	1.13E+05	5.81E+04	3.00E+04	4.18E+04	4.27E+04	1.14E+00	4.83E-01	-1.05E+00	up
Luteolin-8-C-glucoside (Orientin)	Flavonoids	Flavonoid carbonoside	6.20E+05	5.63E+05	4.88E+05	1.26E+05	1.16E+05	2.06E+05	1.34E+00	2.68E-01	-1.90E+00	up
Luteolin-6-C-glucoside (Isoorientin)	Flavonoids	Flavonoid carbonoside	2.65E+05	2.11E+05	1.73E+05	4.49E+04	3.95E+04	6.09E+04	1.36E+00	2.24E-01	-2.16E+00	up
Kaempferol-3-O-galactoside (Trifolin)	Flavonoids	Flavonols	2.97E+05	5.44E+05	4.53E+05	1.55E+05	1.35E+05	1.09E+05	1.30E+00	3.08E-01	-1.70E+00	up
Quercetin-3-O-rhamnoside(Quercitrin)	Flavonoids	Flavonols	2.40E+06	3.68E+06	2.65E+06	3.19E+04	3.55E+04	3.73E+04	1.38E+00	1.20E-02	-6.38E+00	up
Cyanidin-3-O-glucoside (Kuromanin)	Flavonoids	Anthocyanins	1.97E+06	7.47E+06	3.59E+06	1.13E+06	1.90E+06	1.26E+06	1.04E+00	3.29E-01	-1.60E+00	up
Cyanidin-3-O-galactoside	Flavonoids	Anthocyanins	1.87E+06	6.31E+06	3.38E+06	8.47E+05	1.75E+06	8.95E+05	1.09E+00	3.03E-01	-1.72E+00	up
Aromadendrin-7-O-glucoside	Flavonoids	Flavonoid	4.56E+06	7.25E+06	7.06E+06	3.14E+06	2.18E+06	3.12E+06	1.24E+00	4.47E-01	-1.16E+00	up
Epicatechin glucoside	Flavonoids	Flavanols	6.75E+06	1.03E+07	8.06E+06	1.71E+06	1.07E+06	2.15E+06	1.33E+00	1.96E-01	-2.35E+00	up
Gallate catechin gallate	Flavonoids	Flavanols	1.78E+06	5.93E+05	4.81E+05	6.28E+04	1.77E+04	2.18E+05	1.18E+00	1.04E-01	-3.26E+00	up
Epigallocatechin-3-gallate	Flavonoids	Flavanols	2.34E+06	8.67E+05	5.27E+05	3.94E+04	2.02E+04	2.04E+05	1.22E+00	7.05E-02	-3.83E+00	up
Diosmetin-7-O-galactoside	Flavonoids	Flavonoid	2.03E+07	2.62E+07	2.39E+07	5.36E+06	3.95E+06	8.85E+06	1.31E+00	2.58E-01	-1.96E+00	up

Chrysoeriol-6-C-glucoside (Isoscoparin)	Flavonoids	Flavonoid	9.39E+03	1.79E+04	1.04E+04	3.26E+04	4.99E+04	1.79E+04	1.11E+00	2.66E+00	1.41E+00	down
8-Methoxykaempferol-7-O-rhamnoside	Flavonoids	Flavonols	2.64E+04	1.42E+04	1.17E+04	9.56E+03	9.00E+00	9.00E+00	1.04E+00	1.83E-01	-2.45E+00	up
Chrysoeriol-7-O-glucoside	Flavonoids	Flavonoid	3.28E+06	5.48E+06	4.57E+06	8.39E+05	7.26E+05	1.44E+06	1.31E+00	2.26E-01	-2.15E+00	up
6-C-MethylKaempferol-3-glucoside	Flavonoids	Flavonoid	2.04E+07	2.86E+07	2.33E+07	5.42E+06	3.92E+06	8.65E+06	1.31E+00	2.49E-01	-2.00E+00	up
Hispidulin-7-O-Glucoside	Flavonoids	Flavonoid	2.09E+07	2.67E+07	2.33E+07	5.10E+06	4.16E+06	7.48E+06	1.35E+00	2.36E-01	-2.08E+00	up
Dihydroxy-dimethoxyflavone-7-O-glucoside	Flavonoids	Flavonoid	2.99E+04	4.58E+04	3.81E+04	1.07E+04	8.21E+03	1.85E+04	1.26E+00	3.28E-01	-1.61E+00	up
5,2'-Dihydroxy-7,8-dimethoxyflavone glycosides	Flavonoids	Flavonoid	2.61E+04	2.78E+04	2.62E+04	9.00E+00	9.00E+00	1.29E+04	1.05E+00	1.61E-01	-2.64E+00	up
Tricin-4'-methylether-3'-O-glucoside	Flavonoids	Flavonols	8.73E+05	1.02E+06	8.09E+05	4.37E+05	3.90E+05	3.44E+05	1.35E+00	4.34E-01	-1.20E+00	up
Petunidin-3-O-glucoside	Flavonoids	Anthocyanins	6.73E+04	6.44E+04	6.62E+04	9.00E+00	9.00E+00	9.00E+00	1.39E+00	1.36E-04	-1.28E+01	up
Delphinidin-3-O-glucuronide	Flavonoids	Anthocyanins	9.97E+04	9.74E+04	1.17E+05	3.89E+04	2.90E+04	7.73E+04	1.14E+00	4.62E-01	-1.11E+00	up
Kaempferol-3-O-(2''-acetyl)glucoside	Flavonoids	Flavonols	1.58E+04	2.07E+04	1.65E+04	3.93E+03	6.47E+03	6.76E+03	1.31E+00	3.24E-01	-1.63E+00	up
Kaempferol-3-O-(6''-acetyl)glucoside	Flavonoids	Flavonols	2.43E+05	4.65E+05	3.75E+05	1.42E+05	7.14E+04	1.08E+05	1.26E+00	2.98E-01	-1.75E+00	up
Quercetin-3-O-(6''-acetyl)galactoside	Flavonoids	Flavonols	3.35E+04	3.69E+04	4.45E+04	6.32E+03	3.97E+03	8.65E+03	1.34E+00	1.65E-01	-2.60E+00	up
Delphinidin-3-O-(6''-O-acetyl)glucoside	Flavonoids	Anthocyanins	1.02E+05	1.42E+05	1.04E+05	1.65E+04	1.63E+04	3.55E+04	1.32E+00	1.96E-01	-2.35E+00	up
Syringetin-7-O-glucoside	Flavonoids	Flavonoid	3.22E+04	2.74E+04	3.10E+04	1.09E+04	1.61E+04	1.54E+04	1.30E+00	4.68E-01	-1.10E+00	up
Kaempferol-3-O-(6''-malonyl)glucoside	Flavonoids	Flavonols	7.77E+05	1.14E+06	9.75E+05	2.59E+05	1.50E+05	2.20E+05	1.35E+00	2.18E-01	-2.20E+00	up
Kaempferol-3-O-(6''-malonyl)galactoside	Flavonoids	Flavonols	8.33E+05	1.09E+06	9.91E+05	2.70E+05	1.90E+05	2.50E+05	1.36E+00	2.44E-01	-2.04E+00	up
Hinokiflavone	Flavonoids	Biflavones	1.51E+06	2.93E+06	1.66E+06	9.76E+05	4.97E+05	5.43E+05	1.23E+00	3.30E-01	-1.60E+00	up
Amentoflavone	Flavonoids	Biflavones	5.38E+06	9.89E+06	6.05E+06	3.41E+06	1.65E+06	1.87E+06	1.24E+00	3.25E-01	-1.62E+00	up

Cupressuflavone	Flavonoids	Biflavones	1.51E+06	2.98E+06	1.86E+06	1.02E+06	5.07E+05	5.34E+05	1.23E+00	3.24E-01	-1.63E+00	up
Robustaflavone	Flavonoids	Biflavones	1.11E+04	2.30E+04	1.51E+04	8.23E+03	4.15E+03	4.03E+03	1.21E+00	3.33E-01	-1.58E+00	up
Quercetin-7-O-(6''-malonyl)glucoside	Flavonoids	Flavonols	9.35E+05	3.34E+05	1.92E+05	3.91E+04	4.27E+04	5.02E+04	1.29E+00	9.03E-02	-3.47E+00	up
Quercetin-3-O-(6''-malonyl)galactoside	Flavonoids	Flavonols	6.76E+04	8.36E+04	8.35E+04	2.25E+04	1.91E+04	2.29E+04	1.37E+00	2.75E-01	-1.86E+00	up
Isoschaftoside	Flavonoids	Flavonoid carbonoside	5.86E+04	9.61E+04	5.65E+04	3.26E+04	1.45E+04	3.07E+04	1.20E+00	3.68E-01	-1.44E+00	up
Genistein-7-O-galactoside-rhamnose	Flavonoids	Isoflavones	1.39E+05	1.55E+05	1.78E+05	1.54E+04	1.22E+04	3.01E+04	1.34E+00	1.22E-01	-3.03E+00	up
Kaempferol-3,7-O-dirhamnoside (Kaempferitrin)	Flavonoids	Flavonols	2.65E+06	3.30E+06	2.87E+06	1.47E+05	1.03E+05	4.62E+05	1.32E+00	8.06E-02	-3.63E+00	up
Apigenin-7-O-rutinoside (Isorhoifolin)	Flavonoids	Flavonoid	2.02E+05	1.25E+05	1.25E+05	1.55E+04	1.04E+04	1.37E+04	1.37E+00	8.76E-02	-3.51E+00	up
Malvidin-3-O-(6''-O-malonyl)glucoside	Flavonoids	Anthocyanins	4.93E+04	7.65E+04	7.18E+04	8.70E+03	8.42E+03	3.25E+04	1.19E+00	2.51E-01	-1.99E+00	up
Pelargonidin-3-O-rutinoside	Flavonoids	Anthocyanins	5.56E+06	7.59E+06	6.56E+06	7.55E+05	5.39E+05	1.33E+06	1.34E+00	1.33E-01	-2.91E+00	up
Quercetin-3-O-rhamnosyl(1→2)arabinoside	Flavonoids	Flavonols	4.24E+04	6.31E+04	3.21E+04	9.00E+00	9.00E+00	9.00E+00	1.38E+00	1.96E-04	-1.23E+01	up
Luteolin-8-C-glucoside-6-C-arabinoside	Flavonoids	Flavonoid carbonoside	1.60E+04	1.13E+04	1.20E+04	9.00E+00	9.00E+00	9.00E+00	1.39E+00	6.88E-04	-1.05E+01	up
Cyanidin-3-O-sambubioside [Cyanidin-3-O-(2''-O-xylosyl)glucoside]	Flavonoids	Anthocyanins	9.98E+04	1.38E+05	1.24E+05	1.37E+04	9.70E+03	2.35E+04	1.34E+00	1.30E-01	-2.95E+00	up
8,8'-Methylenebiscatechin	Flavonoids	Flavanols	5.14E+04	6.33E+04	5.48E+04	4.91E+03	3.83E+03	1.09E+04	1.33E+00	1.16E-01	-3.11E+00	up
Luteolin-7-O-(6''-caffeoyl)rhamnoside	Flavonoids	Flavonoid	1.62E+05	1.60E+05	9.54E+04	1.71E+04	1.81E+04	1.86E+04	1.37E+00	1.29E-01	-2.96E+00	up
Kaempferol-3-O-(6''-p-Coumaroyl)glucoside (Tiliroside)	Flavonoids	Flavonols	3.93E+05	3.79E+05	3.03E+05	4.79E+04	6.72E+04	5.85E+04	1.37E+00	1.61E-01	-2.63E+00	up

Kaempferol-3-O-(6"-p-Coumaroyl)galactoside	Flavonoids	Flavonoid	1.03E+05	7.32E+04	5.97E+04	9.00E+00	9.00E+00	9.00E+00	1.39E+00	1.14E-04	-1.31E+01	up
Kaempferol-3-O-(2"-p-Coumaroyl)galactoside	Flavonoids	Flavonoid	1.14E+05	9.40E+04	6.20E+04	9.00E+00	9.00E+00	9.00E+00	1.39E+00	1.00E-04	-1.33E+01	up
Kaempferol-3-O-rhamnosyl(1→2)glucoside	Flavonoids	Flavonoid	9.00E+00	9.00E+00	9.00E+00	5.76E+04	9.00E+04	4.84E+04	1.39E+00	7.26E+03	1.28E+01	down
Poncirin (Isosakuranetin-7-O-neohesperidoside)	Flavonoids	Dihydroflavone	5.37E+05	4.35E+05	3.86E+05	6.80E+04	7.41E+04	5.13E+04	1.37E+00	1.42E-01	-2.81E+00	up
Pelargonidin-3,5-O-diglucoside	Flavonoids	Anthocyanins	1.35E+05	1.93E+05	1.29E+05	4.19E+04	4.50E+04	3.94E+04	1.36E+00	2.77E-01	-1.85E+00	up
Kaempferol-3-O-(2"-galloyl)galactoside	Flavonoids	Flavonoid	7.21E+05	1.76E+05	1.18E+04	1.07E+04	5.69E+03	9.22E+03	1.04E+00	2.82E-02	-5.15E+00	up
Kaempferol-3-O-(6"-galloyl)galactoside	Flavonoids	Flavonoid	8.19E+05	1.97E+05	1.91E+04	5.68E+03	1.11E+04	5.45E+03	1.13E+00	2.14E-02	-5.54E+00	up
Kaempferol-3-O-(6"-galloyl)glucoside	Flavonoids	Flavonoid	7.85E+05	1.70E+05	2.83E+04	5.72E+03	3.99E+03	3.42E+03	1.24E+00	1.33E-02	-6.23E+00	up
"Eriodictyol-7-O-(6""-O-galloyl)glucoside"	Flavonoids	Dihydroflavone	1.00E+04	1.10E+04	1.98E+04	9.00E+00	9.00E+00	6.87E+03	1.04E+00	1.69E-01	-2.56E+00	up
Quercetin-3-O-(6"-p-Coumaroyl)galactoside	Flavonoids	Flavonols	2.23E+05	1.65E+05	1.19E+05	2.80E+04	2.25E+04	2.57E+04	1.37E+00	1.51E-01	-2.73E+00	up
Quercetin-3-O-(6"-p-Coumaroyl)glucoside	Flavonoids	Flavonols	2.03E+05	1.54E+05	1.12E+05	1.81E+04	1.89E+04	2.14E+04	1.37E+00	1.25E-01	-3.00E+00	up
Luteolin-6,8-di-C-glucoside	Flavonoids	Flavonoid carbonoside	2.44E+05	1.58E+05	1.76E+05	5.23E+04	5.91E+04	8.70E+04	1.30E+00	3.43E-01	-1.54E+00	up
Luteolin-6-C-glucoside-7-O-glucoside	Flavonoids	Flavonoid carbonoside	5.10E+04	3.36E+04	4.63E+04	9.49E+03	2.12E+04	2.00E+04	1.19E+00	3.87E-01	-1.37E+00	up

Kaempferol-6,8-di-C-glucoside	Flavonoids	Flavonoid	1.28E+05	9.71E+04	7.41E+04	2.44E+04	4.29E+04	4.93E+04	1.21E+00	3.90E-01	-1.36E+00	up
Petunidin-3-O-glucoside-5-O-arabinoside	Flavonoids	Anthocyanins	6.79E+04	1.12E+05	8.79E+04	4.86E+04	3.00E+04	3.52E+04	1.25E+00	4.25E-01	-1.23E+00	up
Cyanidin-3,5-O-diglucoside (Cyanin)	Flavonoids	Anthocyanins	8.86E+04	1.24E+05	1.01E+05	5.89E+04	3.30E+04	4.65E+04	1.26E+00	4.42E-01	-1.18E+00	up
Quercetin-3-O-(6"-galloyl)glucoside	Flavonoids	Flavonols	4.22E+05	8.79E+04	9.24E+03	9.00E+00	9.00E+00	9.00E+00	1.35E+00	5.20E-05	-1.42E+01	up
Quercetin-3-O-(6"-galloyl)galactoside	Flavonoids	Flavonols	4.18E+05	5.86E+04	1.13E+04	9.00E+00	9.00E+00	9.00E+00	1.36E+00	5.54E-05	-1.41E+01	up
Chrysoeriol-6-C-glucoside-4'-O-glucoside	Flavonoids	Flavonoid	7.14E+04	7.76E+04	8.05E+04	3.69E+04	2.47E+04	3.66E+04	1.33E+00	4.28E-01	-1.23E+00	up
Peonidin-3,5-O-diglucoside	Flavonoids	Anthocyanins	2.50E+04	2.16E+04	3.63E+04	8.76E+04	5.99E+04	8.05E+04	1.30E+00	2.75E+00	1.46E+00	down
6-Hydroxykaempferol-3,6-O-Diglucoside	Flavonoids	Flavonols	3.29E+05	5.70E+05	7.19E+05	1.13E+06	1.11E+06	1.15E+06	1.21E+00	2.09E+00	1.07E+00	down
Delphinidin-3-O-(6"-O-caffeoyl)glucoside	Flavonoids	Anthocyanins	7.21E+04	4.81E+04	3.95E+04	1.20E+04	1.96E+04	8.27E+03	1.27E+00	2.50E-01	-2.00E+00	up
Bilobetin-7-O-glucoside	Flavonoids	Biflavones	3.52E+06	2.88E+06	2.99E+06	1.31E+06	7.83E+05	1.51E+06	1.29E+00	3.84E-01	-1.38E+00	up
Quercetin-3-O-(2"-O-Rhamnosyl)rutinoside	Flavonoids	Flavonols	7.17E+06	1.42E+06	2.05E+03	9.00E+00	9.00E+00	9.00E+00	1.26E+00	3.14E-06	-1.83E+01	up
Vitexin-7-O-(6"-feruloyl)glucoside	Flavonoids	Flavonoid carbonoside	5.45E+04	2.71E+04	3.12E+04	2.28E+04	1.83E+04	8.15E+03	1.06E+00	4.36E-01	-1.20E+00	up
Catechin-catechin-catechin	Flavonoids	Flavanols	1.17E+06	1.57E+06	1.30E+06	5.73E+04	2.73E+04	1.20E+05	1.34E+00	5.07E-02	-4.30E+00	up
Cyanidin-3-O-rutinoside-5,3'-di-O-glucoside	Flavonoids	Anthocyanins	1.46E+04	3.78E+04	2.49E+04	7.42E+04	7.19E+04	6.16E+04	1.23E+00	2.68E+00	1.42E+00	down
