# Supplementary material for: Transcriptomics integrated with widely targeted metabolomics reveals the cold resistance mechanism in Hevea brasiliensis
Source: Front Plant Sci. 2023 Jan 10;13:1092411. doi: 10.3389/fpls.2022.1092411 (PMC9871781; doi:10.3389/fpls.2022.1092411)
Supplement: Supplementary file 2 [file DataSheet_2.pdf]

(A)

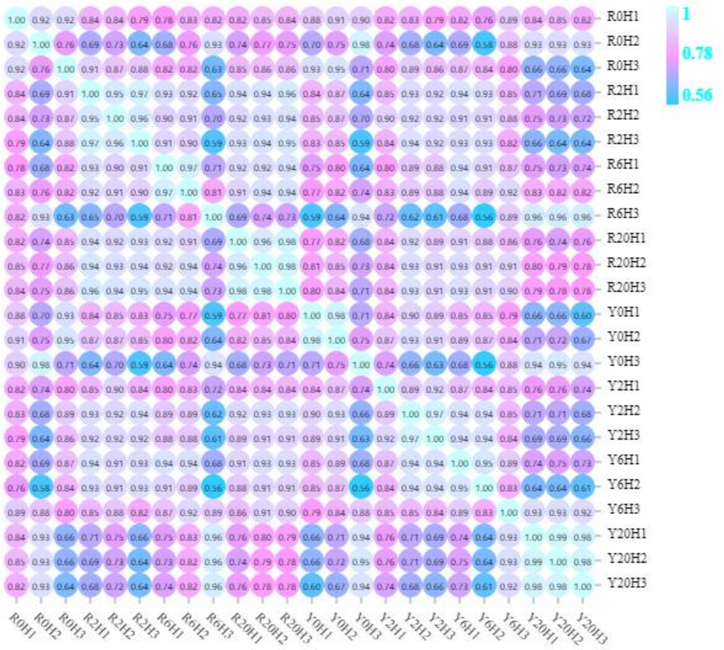

(B)

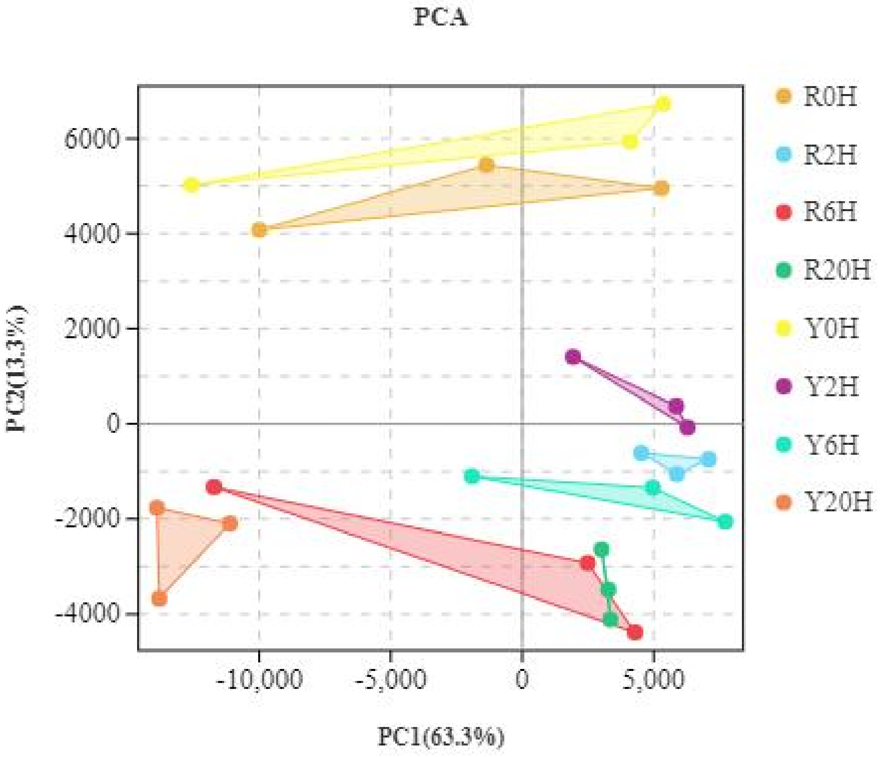

Supplementary Figure 1. The correlation heatmap (A) and PCA plot (B) for all samples'transcriptomic data of rubber tree

(A) R0H_vs_Y0H

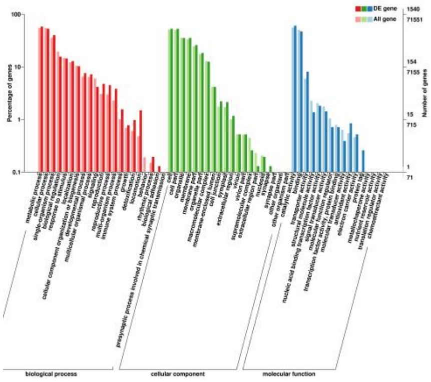

(B) R2H_vs_Y2H

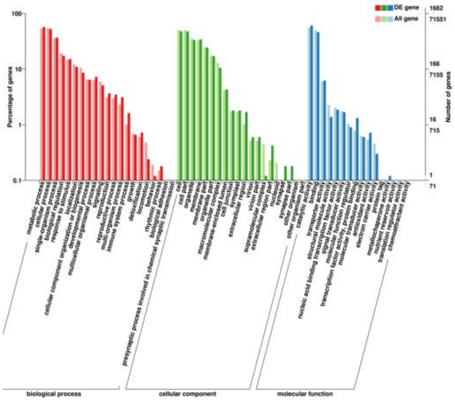

(C) R6H_vs_Y6H

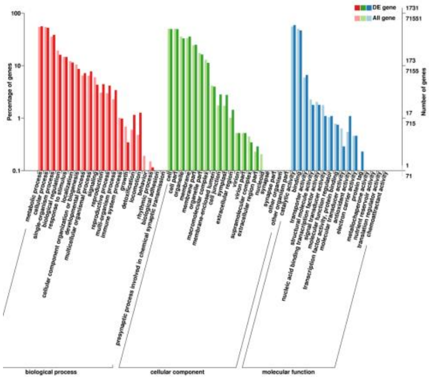

(D) R20H_vs_Y20H

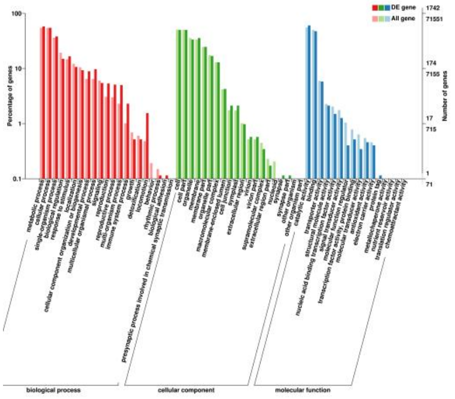

(E) R0H_vs_Y0H

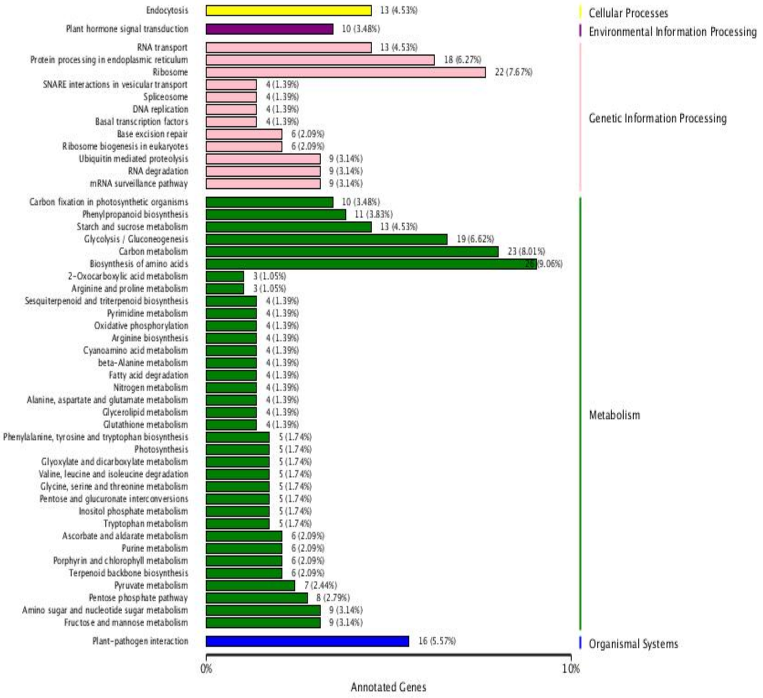

(F) R2H_vs_Y2H

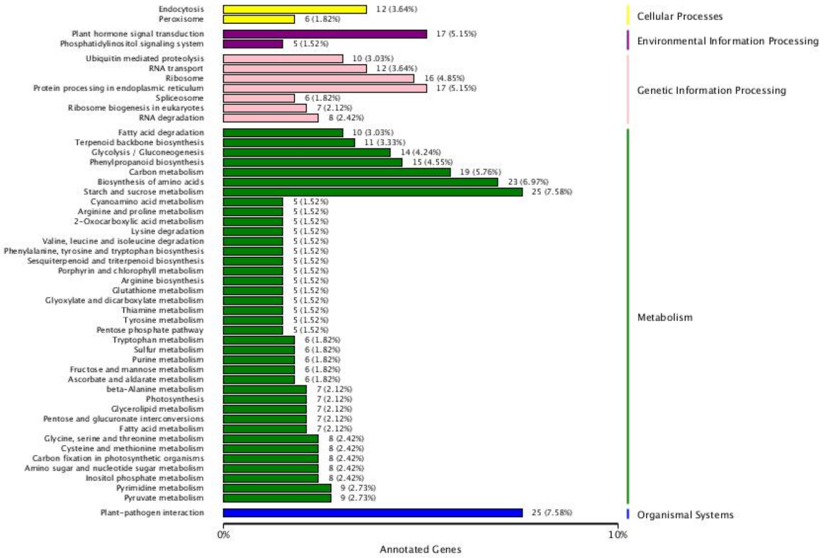

(G) R6H_vs_Y6H

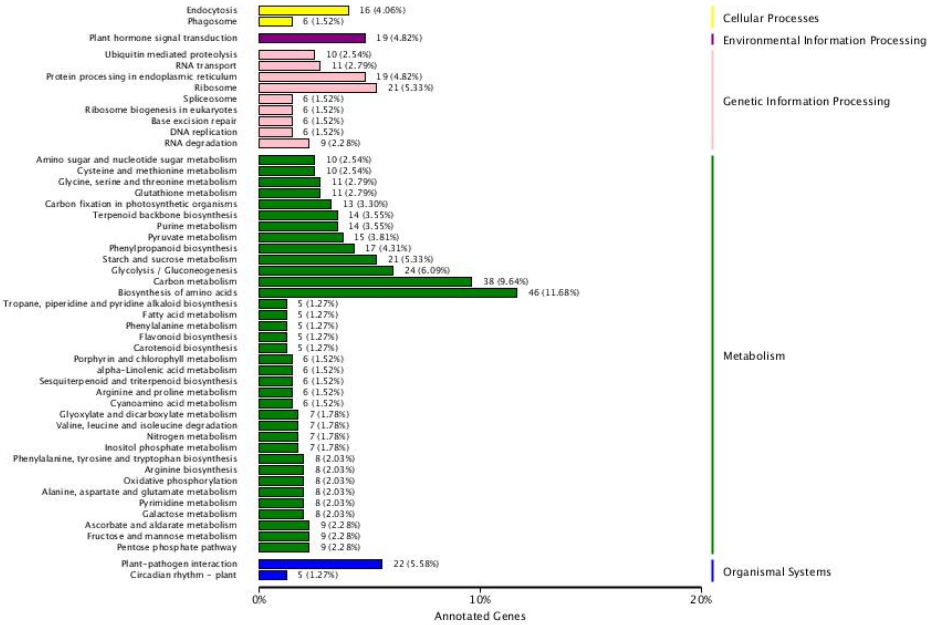

(H) R20H_vs_Y20H

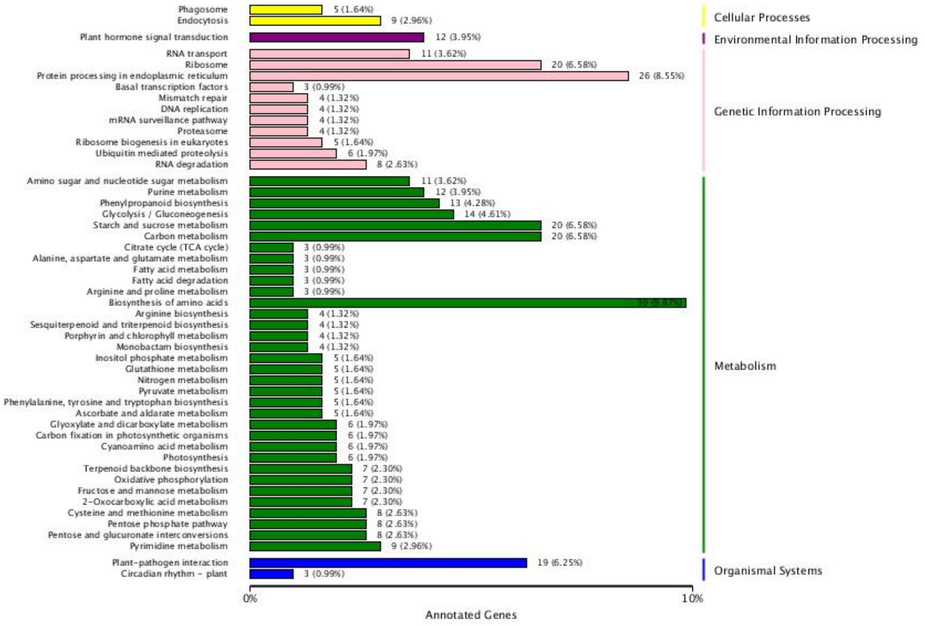

Supplementary Figure 2. GO annotation and KEGG annotation of DEGs of the rubber tree. A–D: GO annotation; E–H: KEGG pathway analysis

(A) positive ion mode

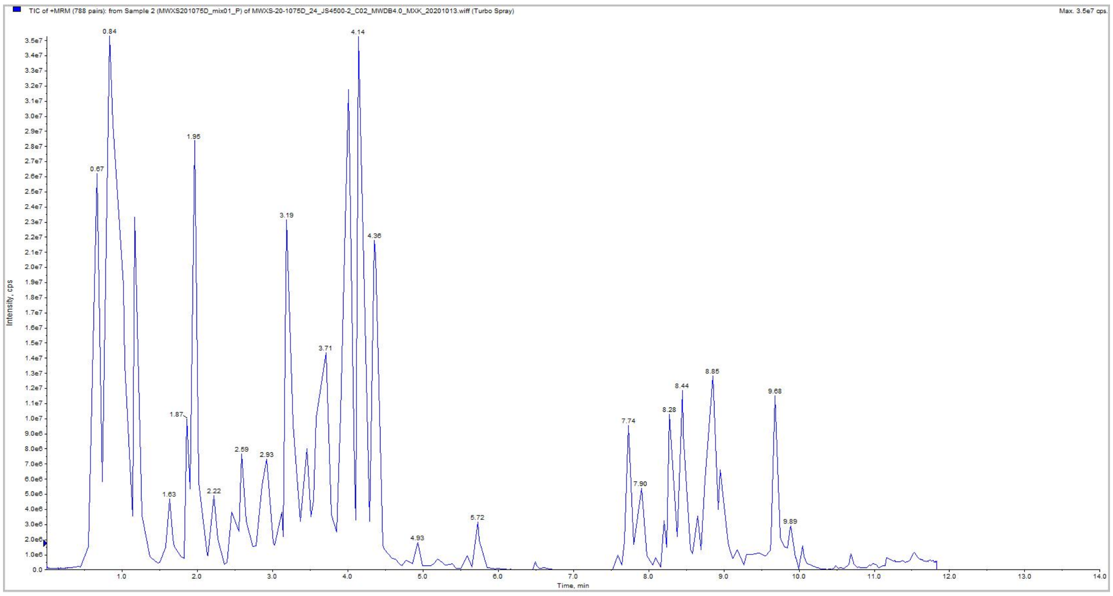

(B) positive ion mode

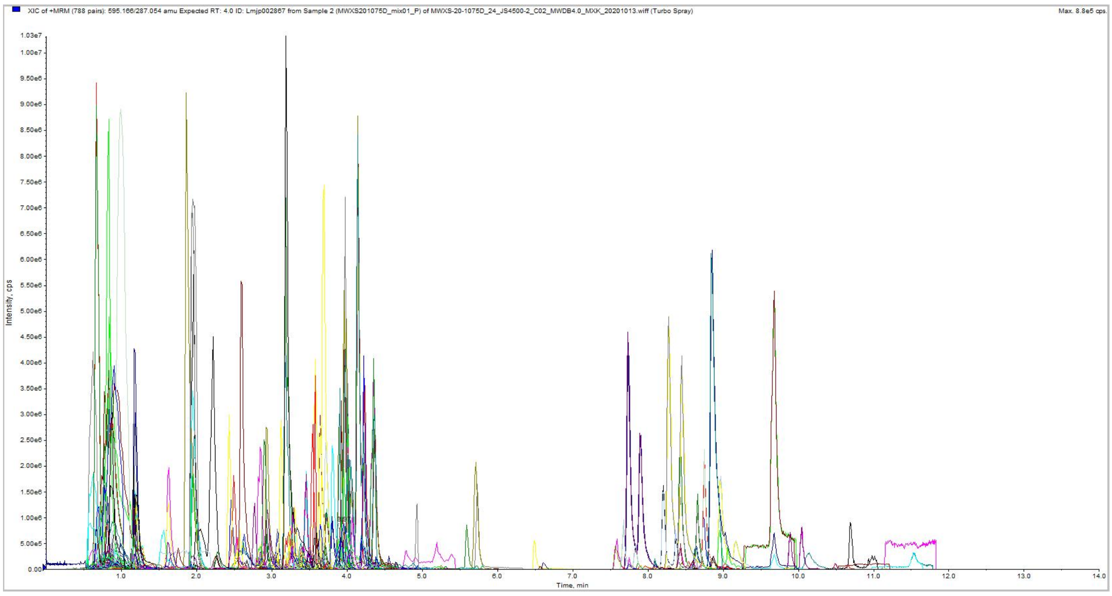
XIC of +MRM (788 pairs): 595.166/287.054 amu Expected RT: 4.0 ID: Lmjp002867 from Sample 2 (MWXS201075D_mix01_P) of MWXS-20-1075D_24_JS4500-2_C02_MWDB4.0_MXK_20201013.wiff (Turbo Spray)
Max. 8.8e5 cps.
Intensity, cps
Time, min

C

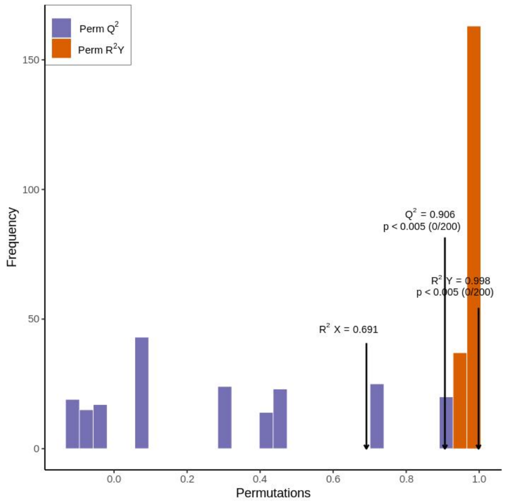
Perm Q2
Perm R2Y
Frequency
Permutations
Q2 = 0.906
p < 0.005 (0/200)
R2Y = 0.998
p < 0.005 (0/200)
R2X = 0.691

D

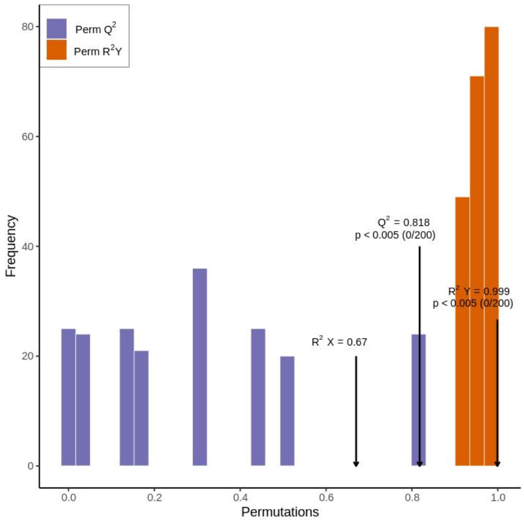
Perm Q2
Perm R2Y
Frequency
Permutations
Q2 = 0.818
p < 0.005 (0/200)
R2Y = 0.999
p < 0.005 (0/200)
R2X = 0.67

E

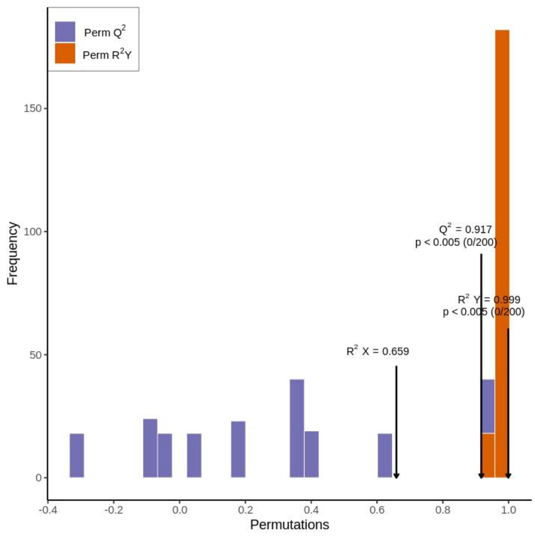

F

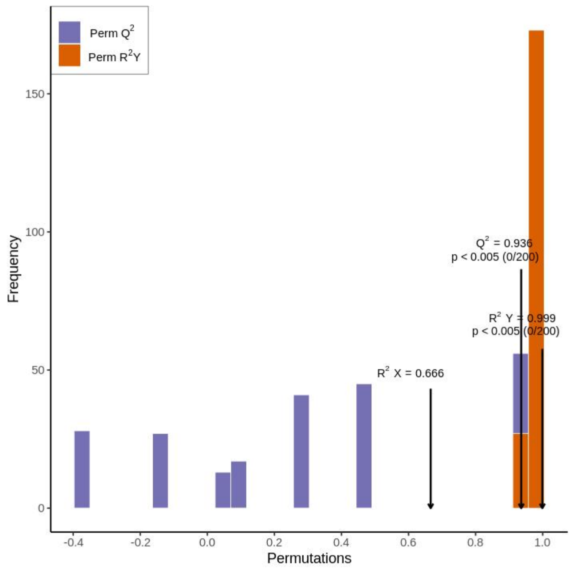

Supplementary Figure 3. The analysis of metabolites of all samples. (A–B) the TIC plots of the MS detection; OPLS-DA validation plots of metabolites identified between (C) R0H and Y0H, (D) R2H and Y2H, (E) R6H and Y6H, and (F) R20H and Y20H. The OPLS-DA model was verified with 200 random permutations

R0H_vs_Y0H

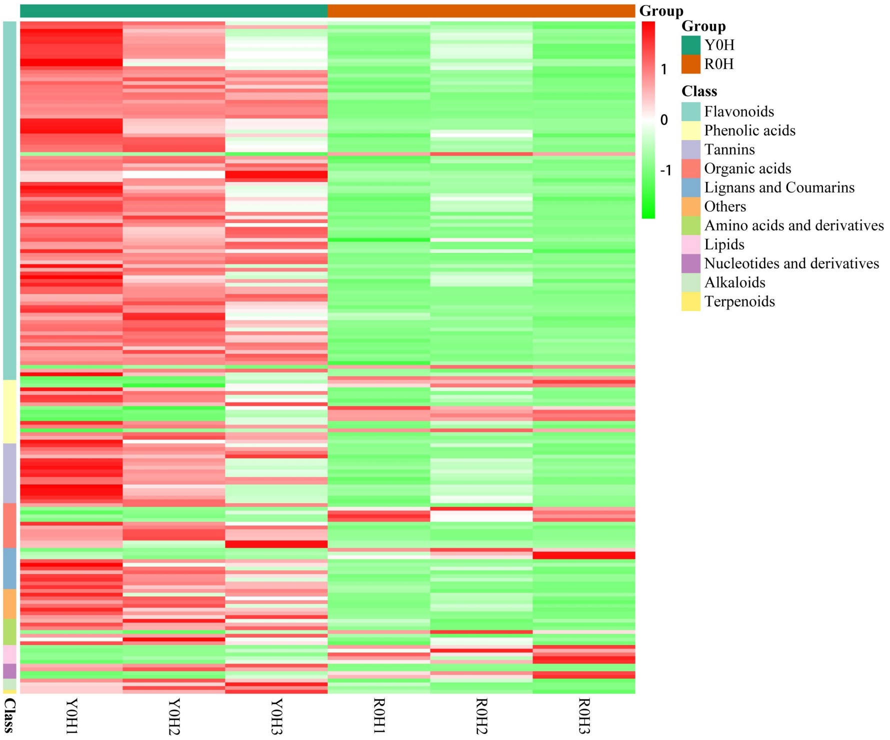

R2H_vs_Y2H

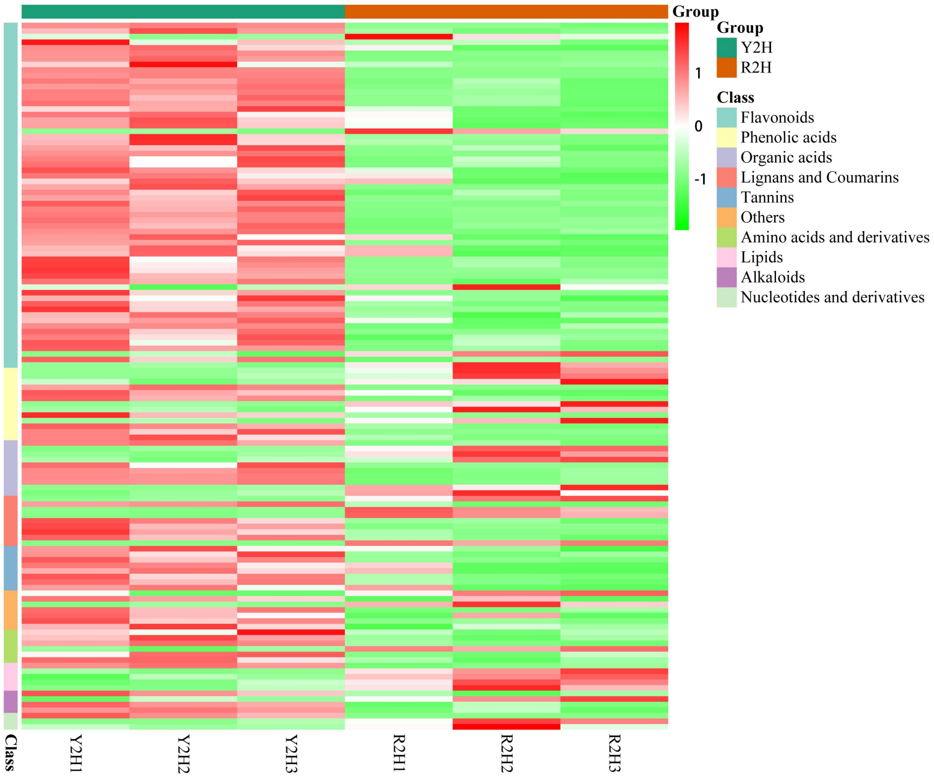

R6H_vs_Y6H

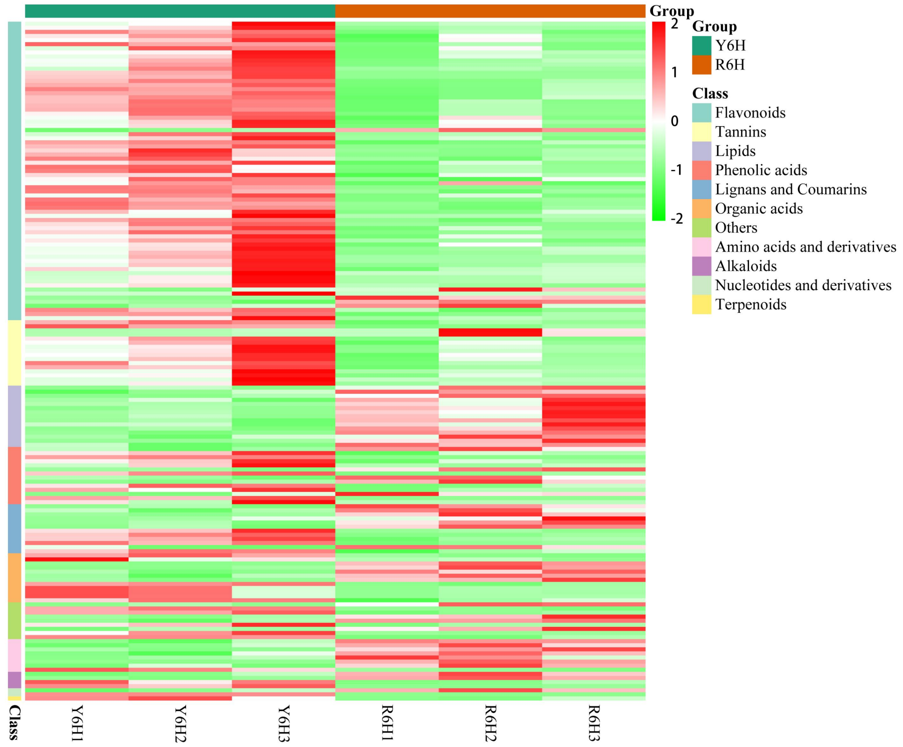

R20H_vs_Y20H

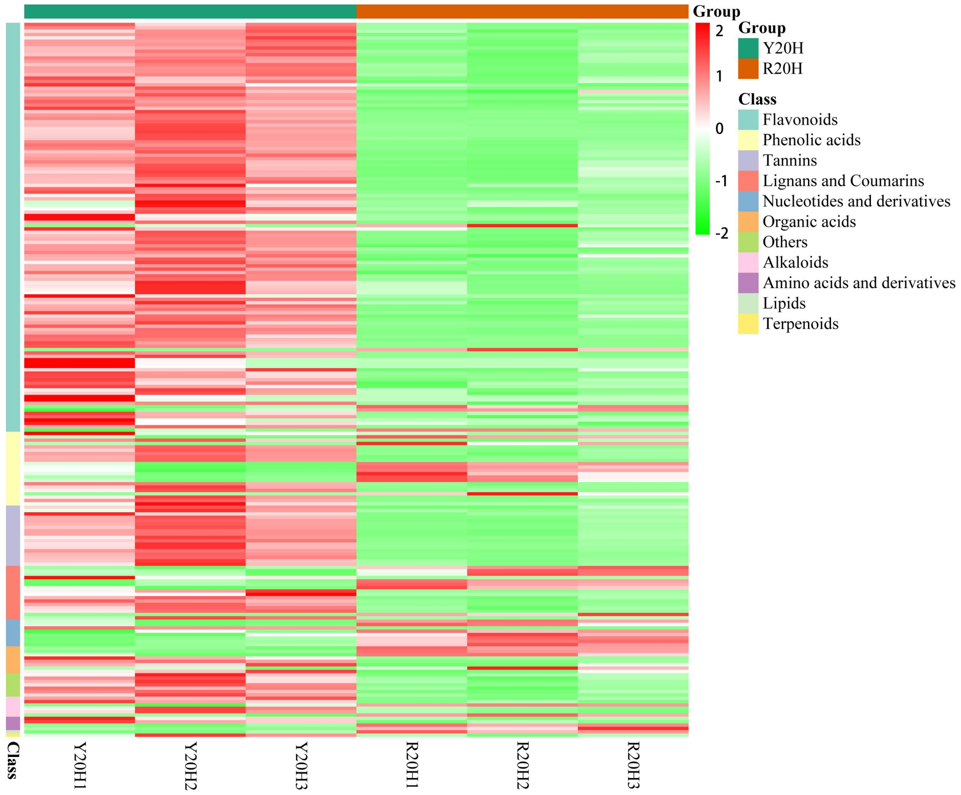

Supplementary Figure 4. The heatmap of the DAMs of rubber tree

(A) KEGG analysis of different treatment point

R0H_vs_Y0H

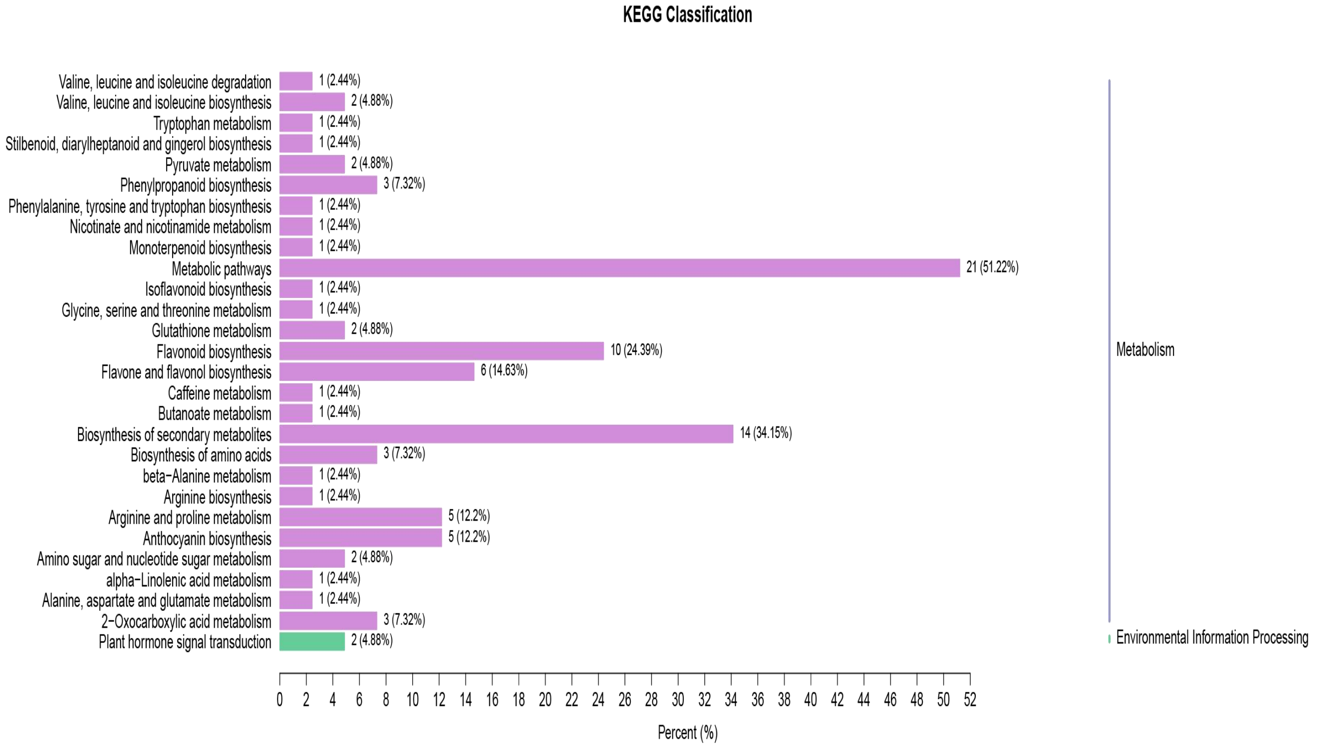

R2H_vs_Y2H

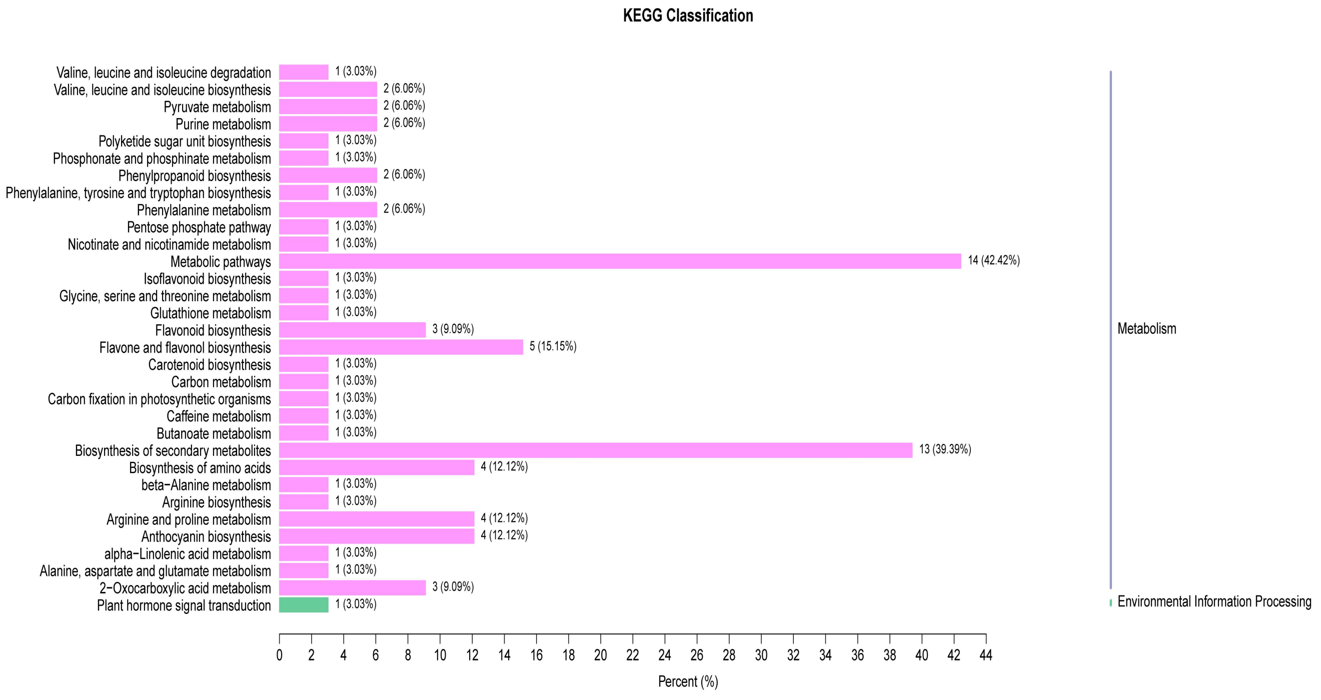

R6H_vs_Y6H

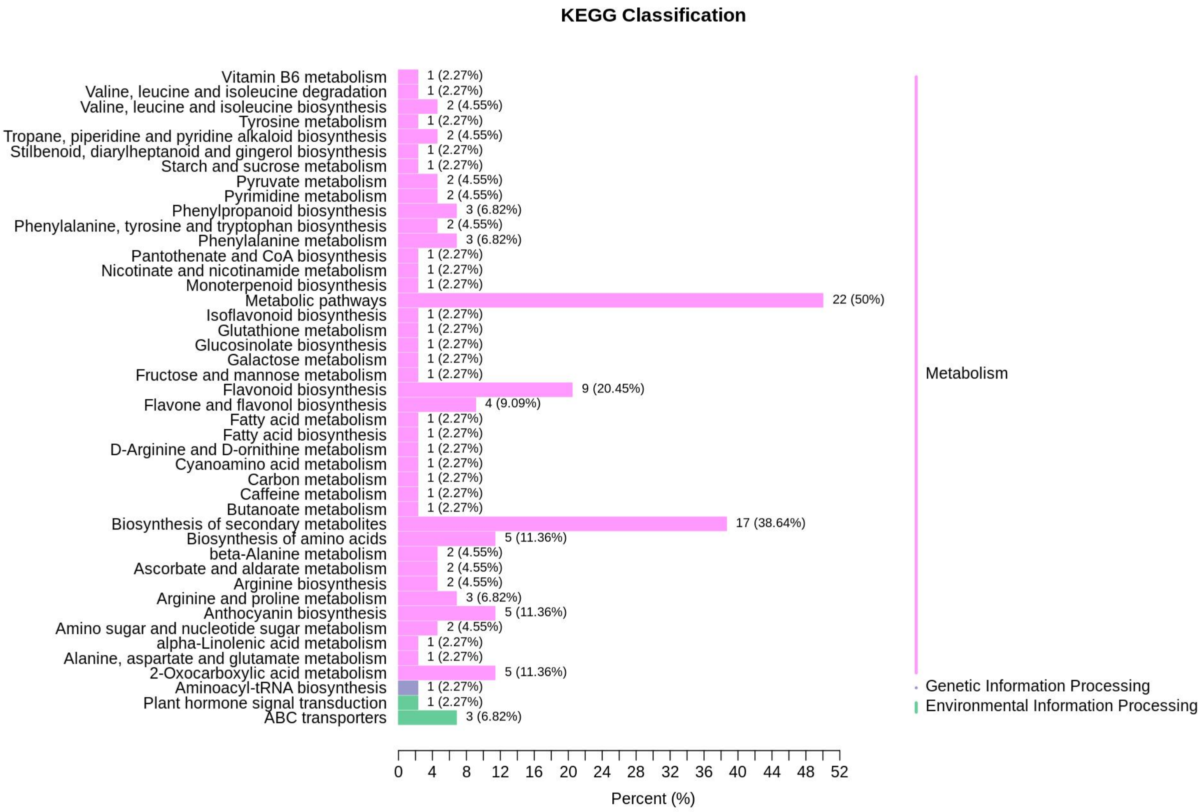

R20H_vs_Y20H

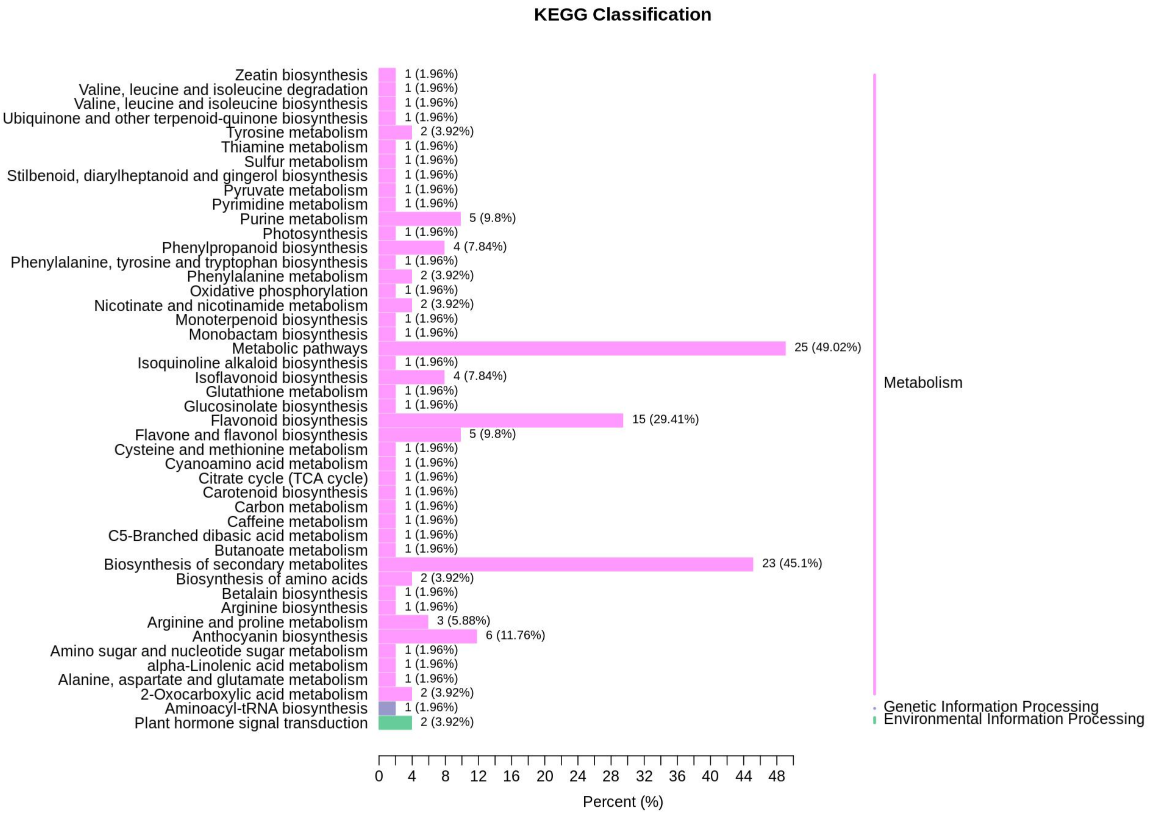

(B) KEGG enrichment of different treatment point

R0H_vs_Y0H

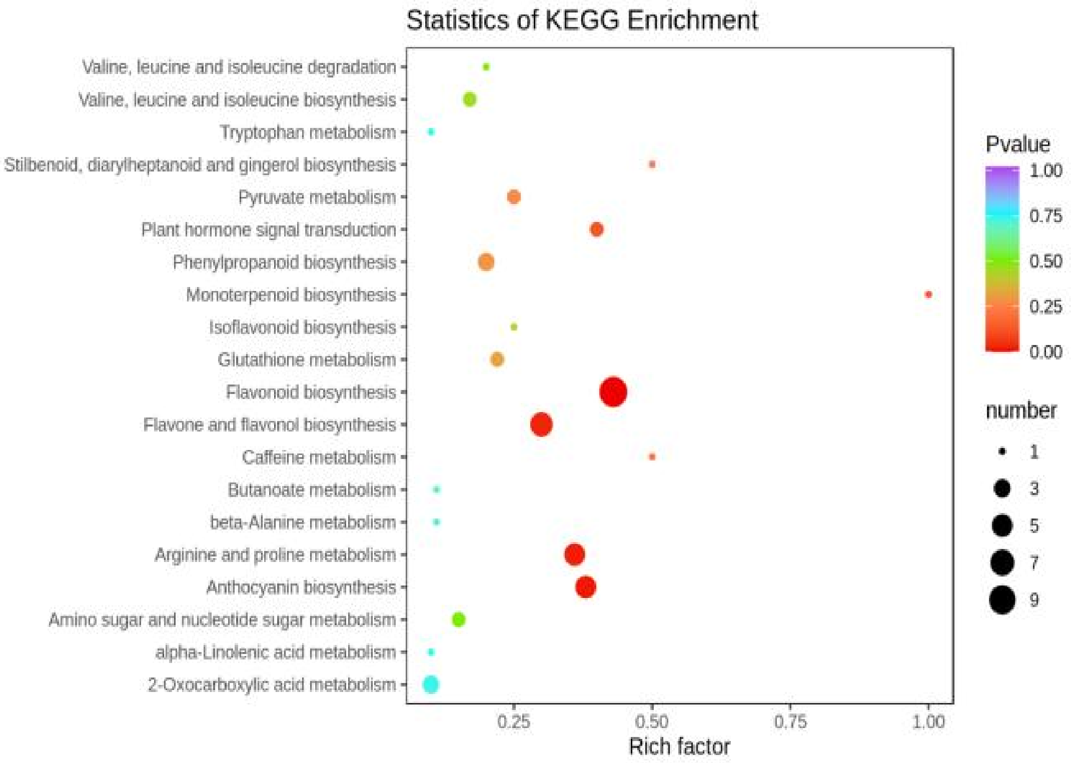

R2H_vs_Y2H

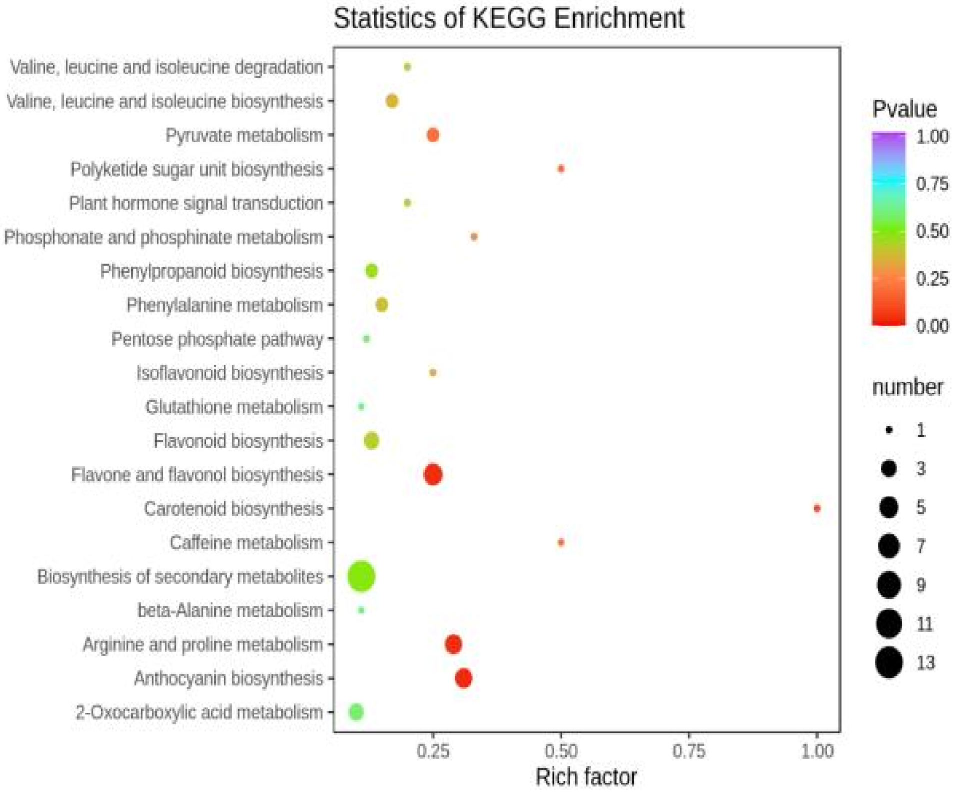

R6H_vs_Y6H

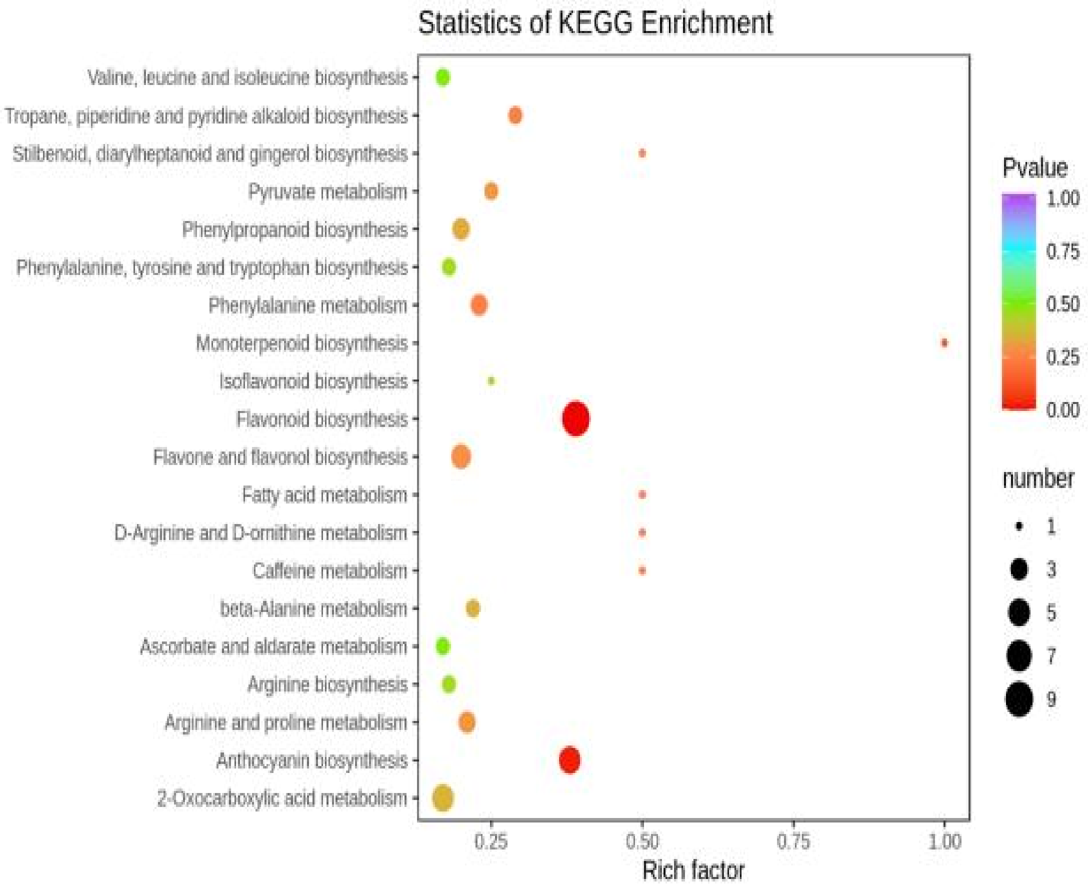

R20H_vs_Y20H

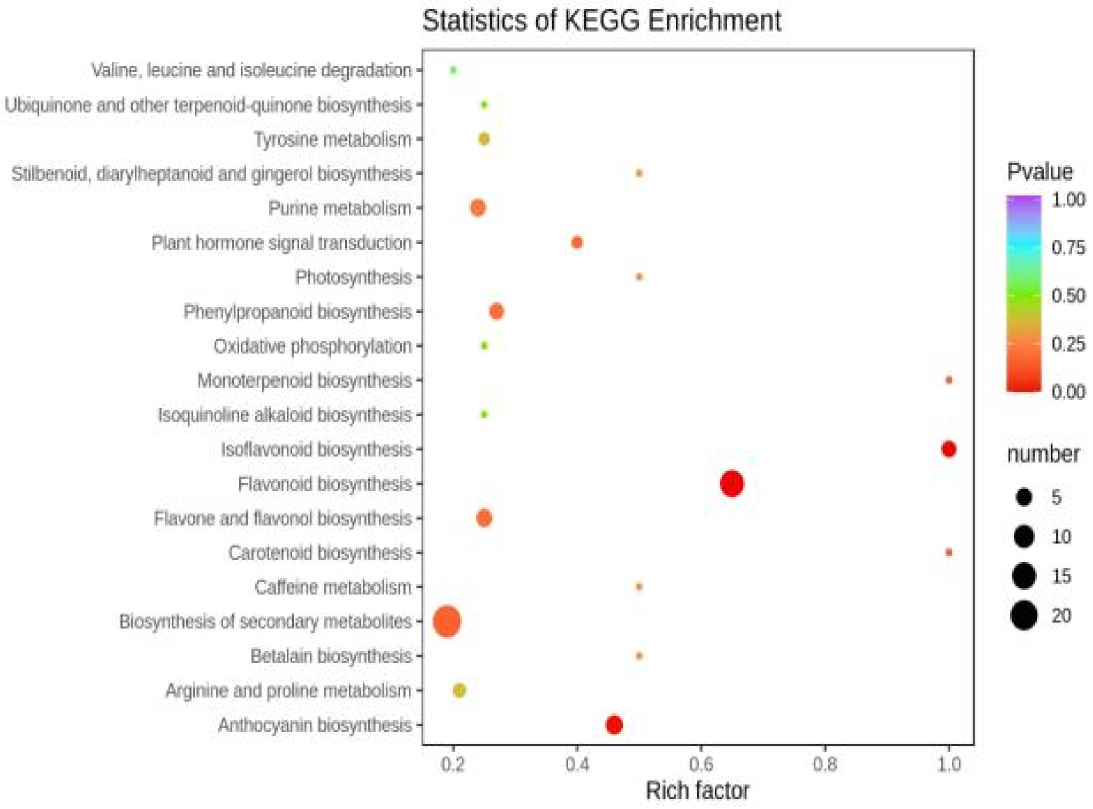

Supplementary Figure 5. KEGG analysis of DAMs. A: KEGG classification of DAMs identified after different treatment durations; B: KEGG enrichment of DAMs identified after different treatment durations. The abscissa represents the rich factor of each pathway (Rich factor was calculated as the ratio of the number of differentially expressed genes annotated in a pathway to the number of all genes annotated in this pathway), and the ordinate represents the pathway's name. The dot's color represents the p-value; the more significant the enrichment, the more intense the red. The size of the dot represents the number of differential metabolites enriched.

(A) The KEGG enrichment for the down-accumulated DAMs:

R0H_vs_Y0H

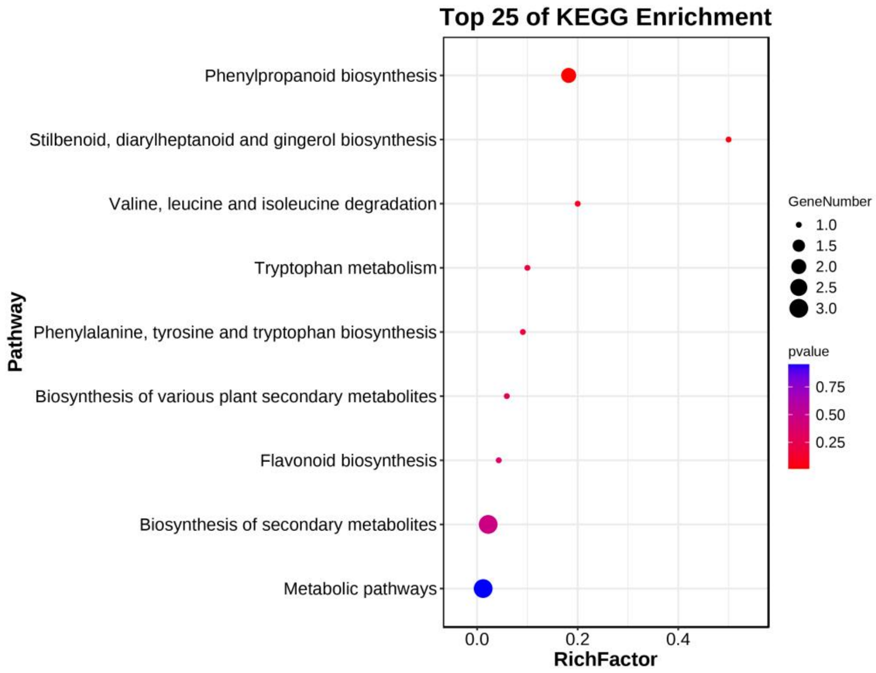

R2H_vs_Y2H

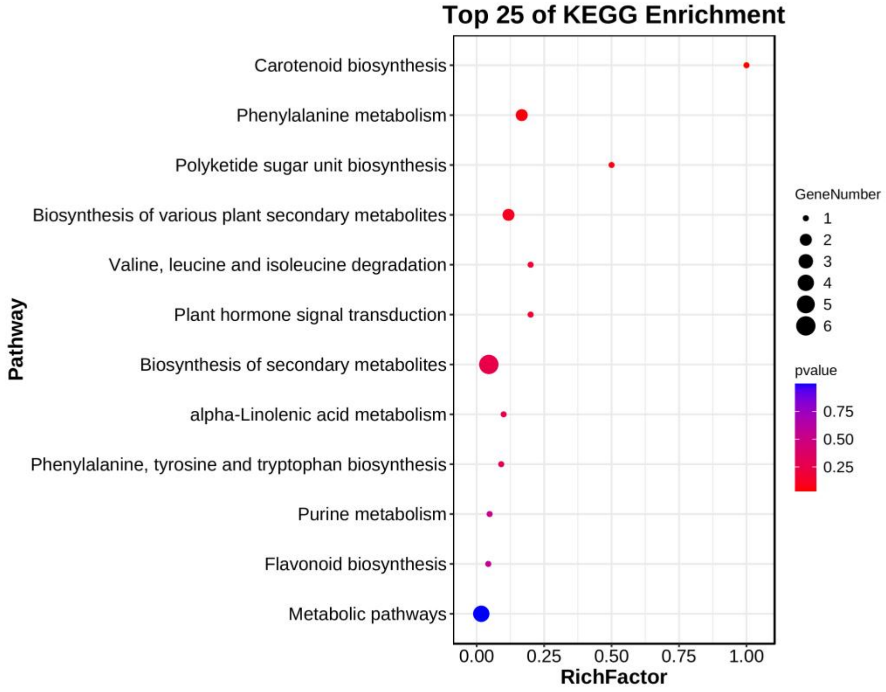

R6H_vs_Y6H

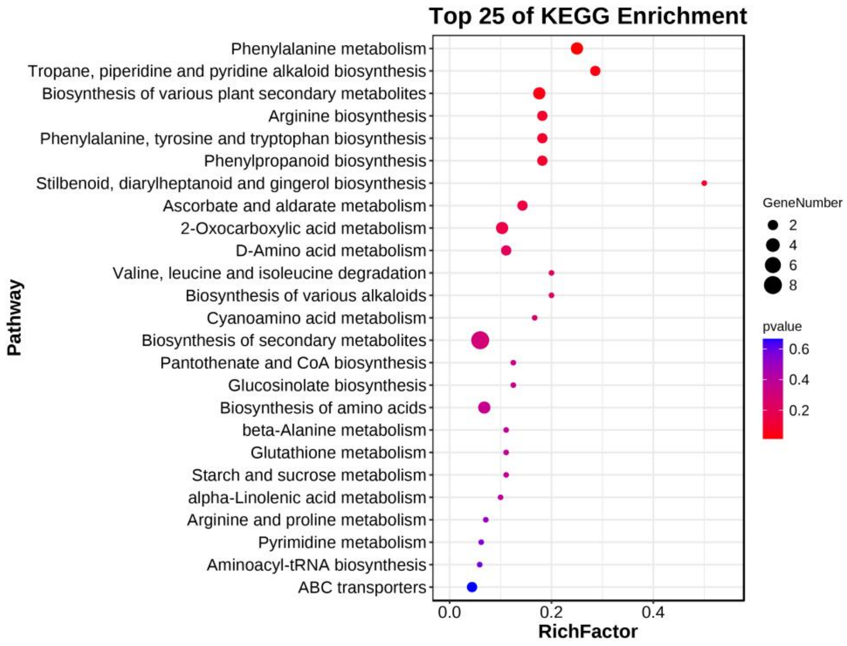

R20H_vs_Y20H

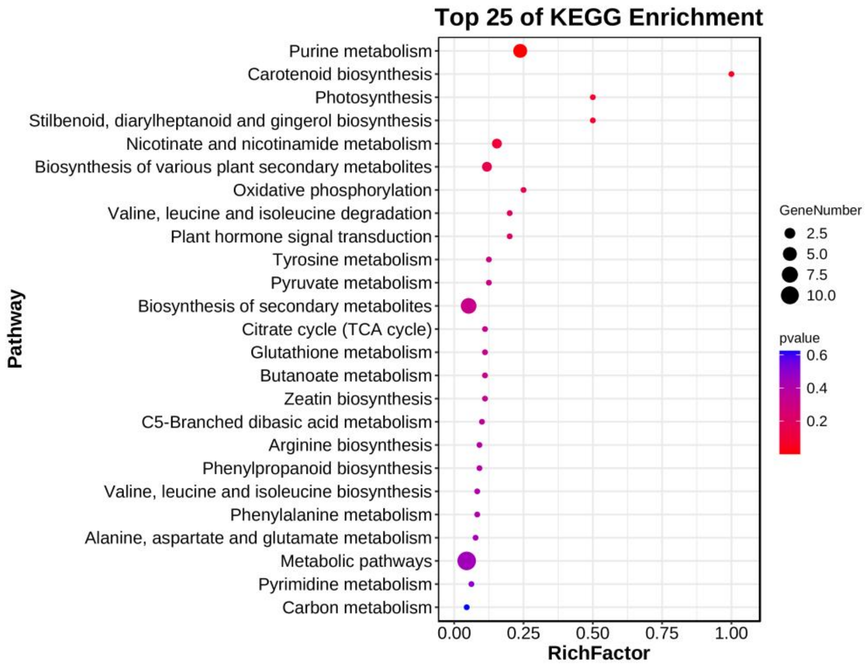

(B) The KEGG enrichment for the up-accumulated DAMs:

R0H_vs_Y0H

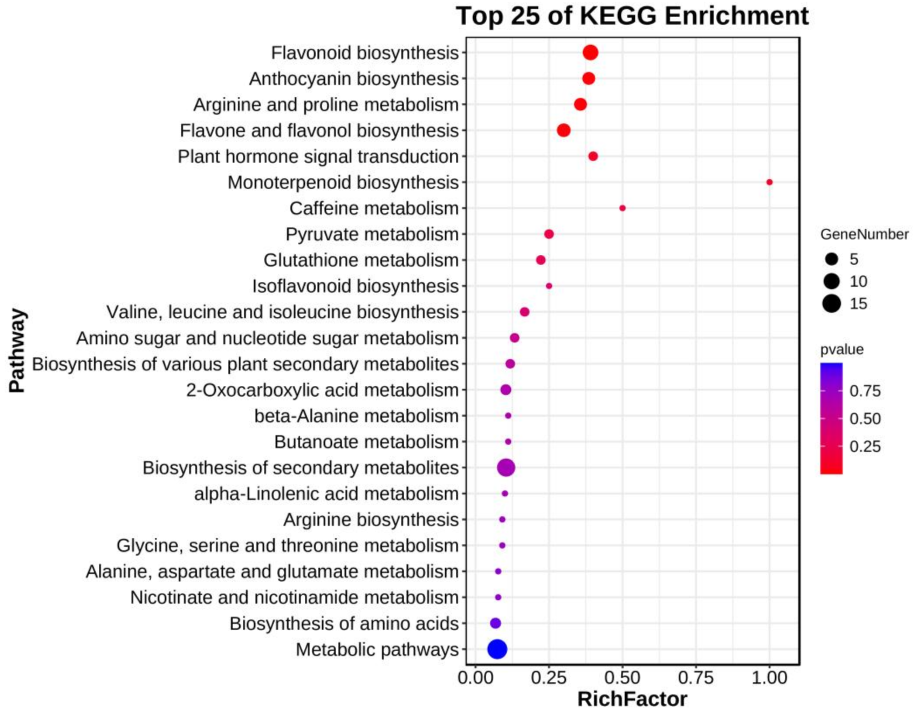

R2H_vs_Y2H

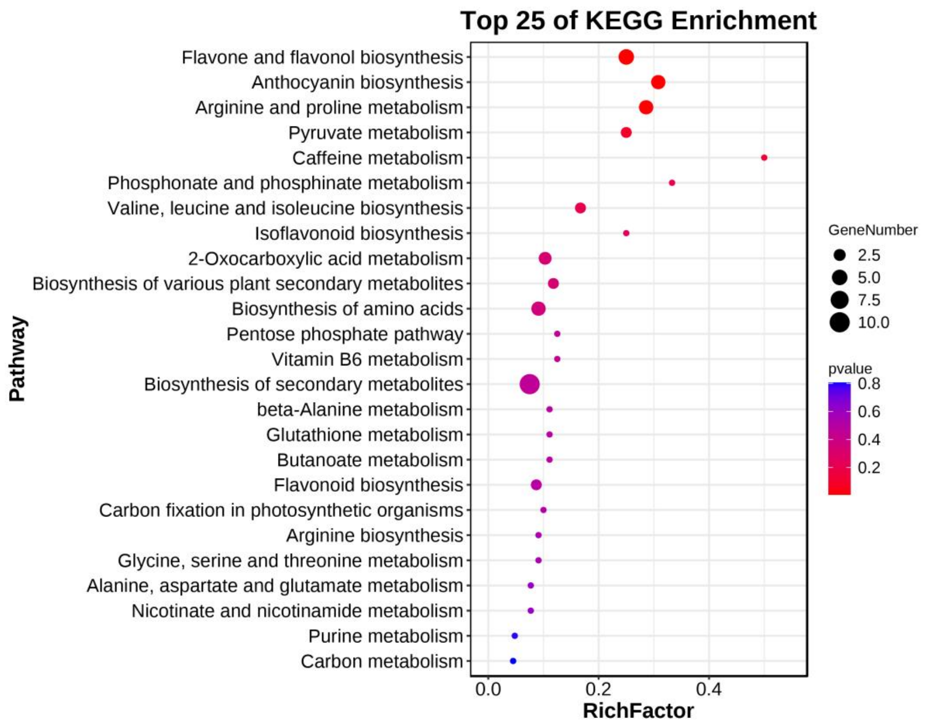

R6H_vs_Y6H

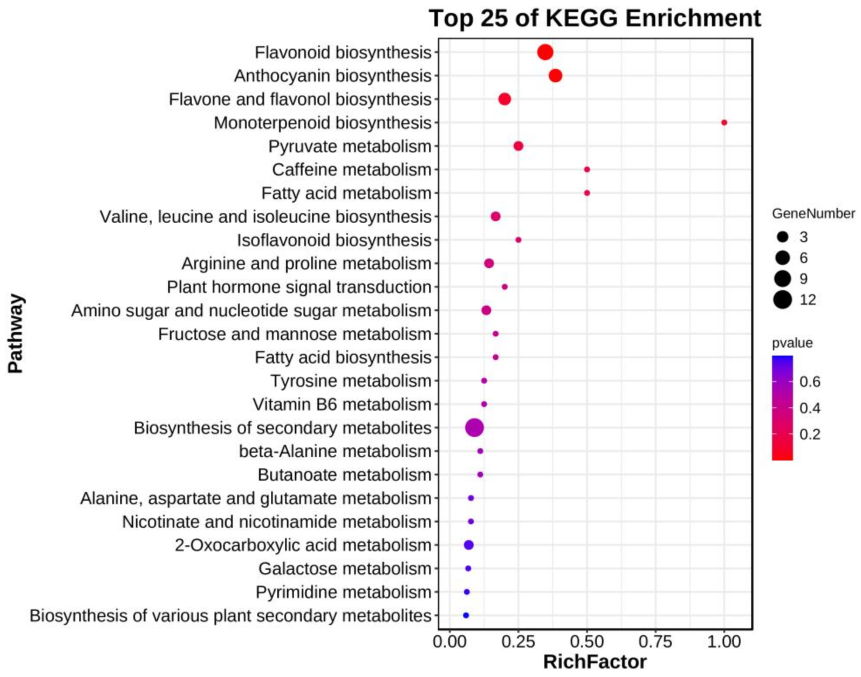

R20H_vs_Y20H

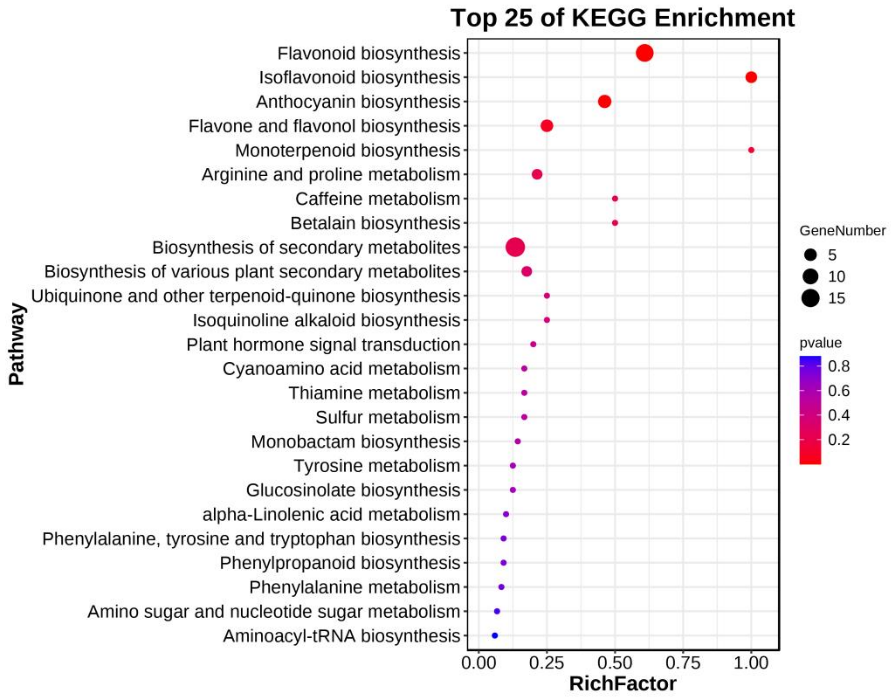

Supplementary Figure 6. The KEGG enrichment analysis of the upregulated and downregulated metabolites of rubber tree

(A) KEGG enrichment

R0H_vs_Y0H

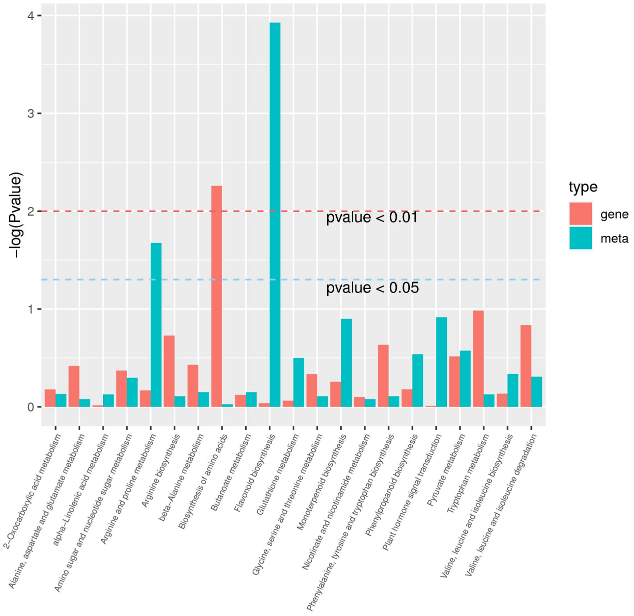

R2H_vs_Y2H

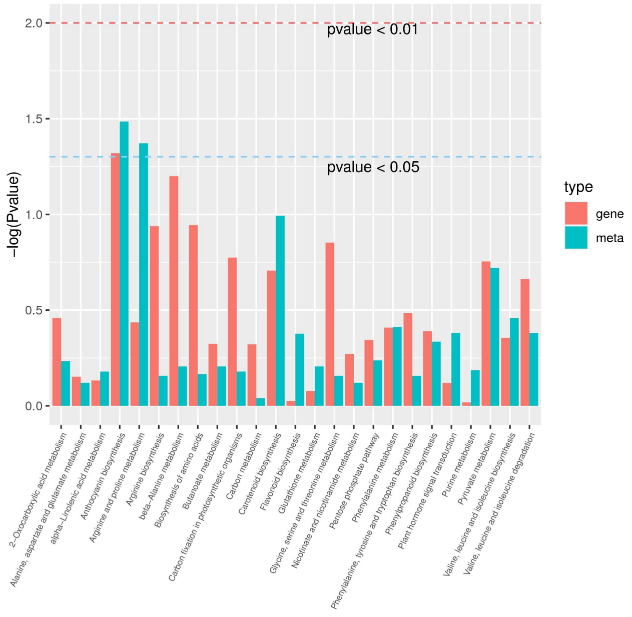

R6H_vs_Y6H

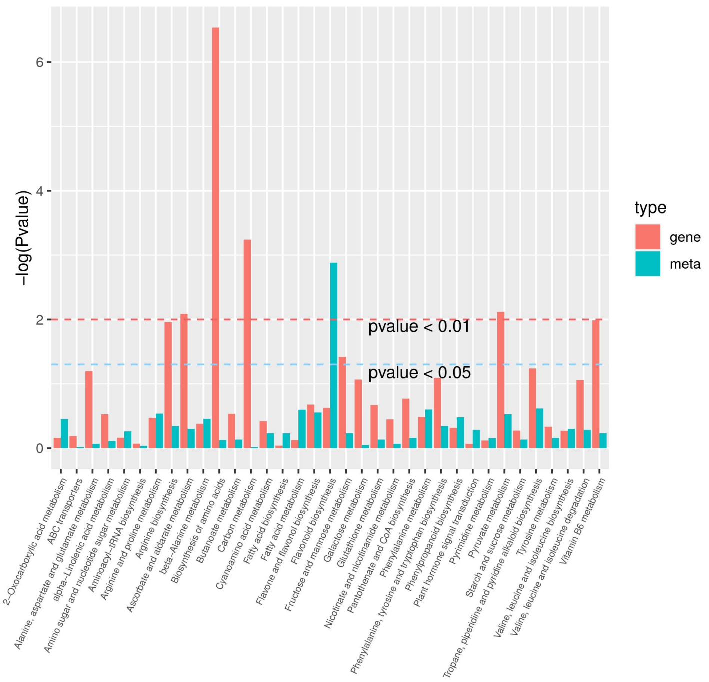

R20H_vs_Y20H

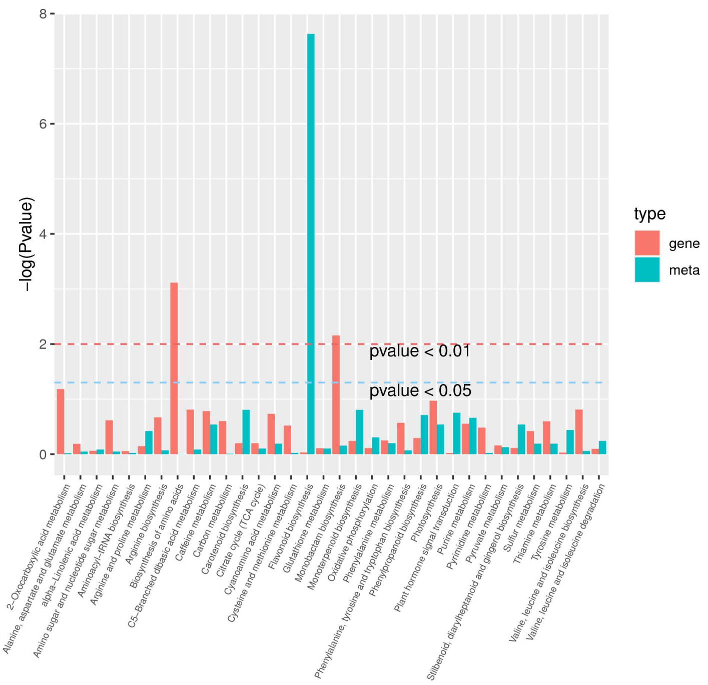

(B) heatmap

R0H_vs_Y0H

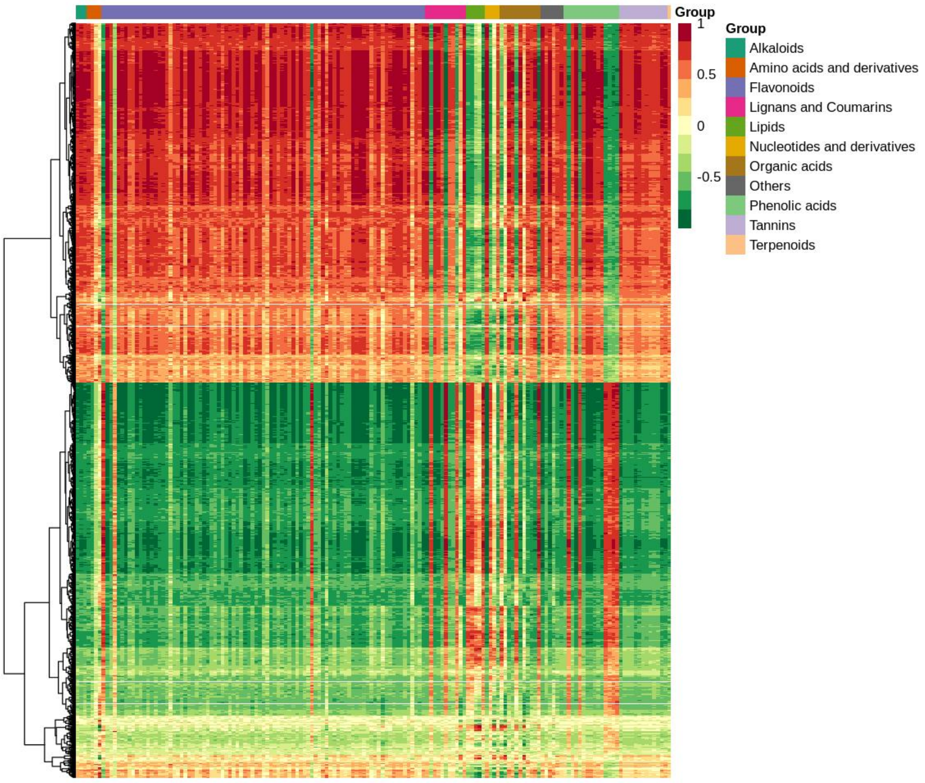

R2H_vs_Y2H

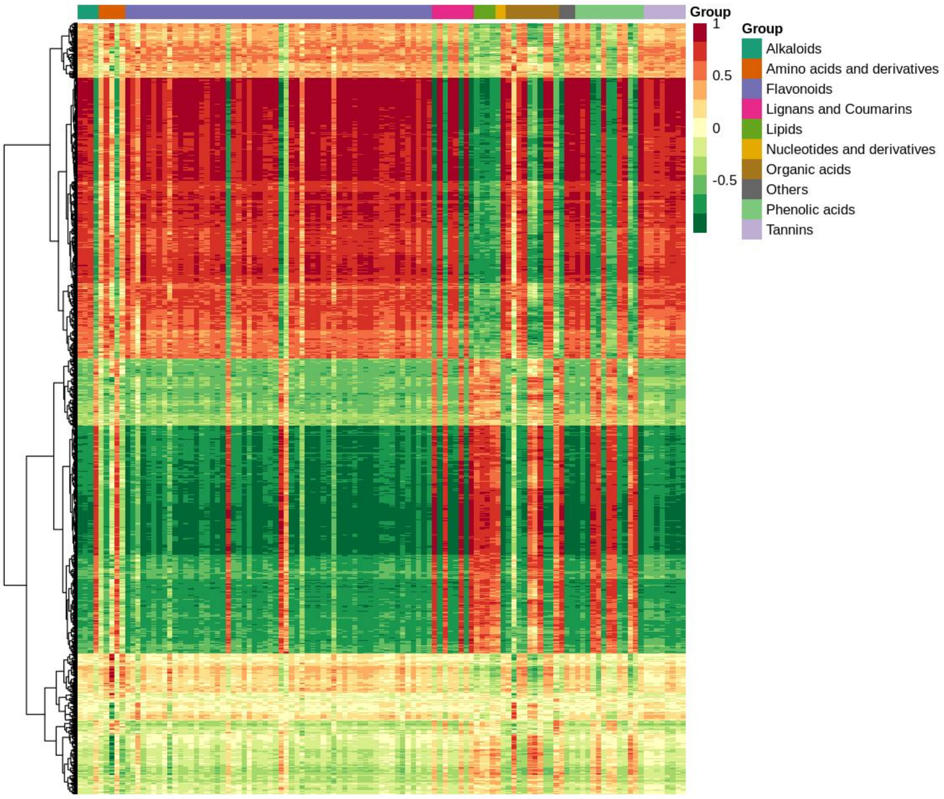

Group
1
0.5
0
-0.5
Group
Alkaloids
Amino acids and derivatives
Flavonoids
Lignans and Coumarins
Lipids
Nucleotides and derivatives
Organic acids
Others
Phenolic acids
Tannins

R6H_vs_Y6H

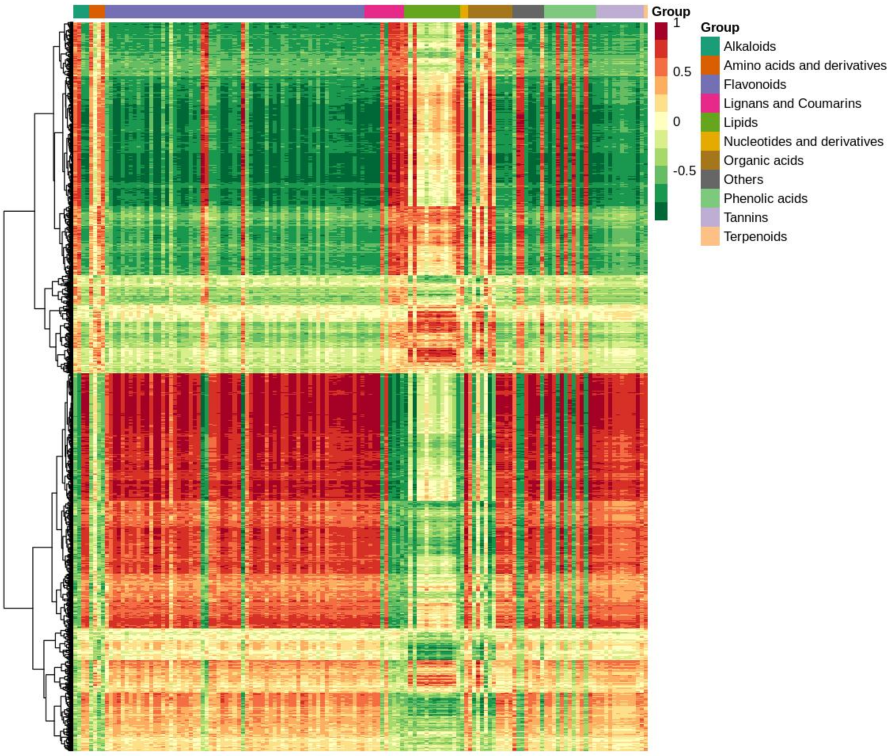

Group
1
0.5
0
-0.5
Group
Alkaloids
Amino acids and derivatives
Flavonoids
Lignans and Coumarins
Lipids
Nucleotides and derivatives
Organic acids
Others
Phenolic acids
Tannins
Terpenoids

R20H_vs_Y20H

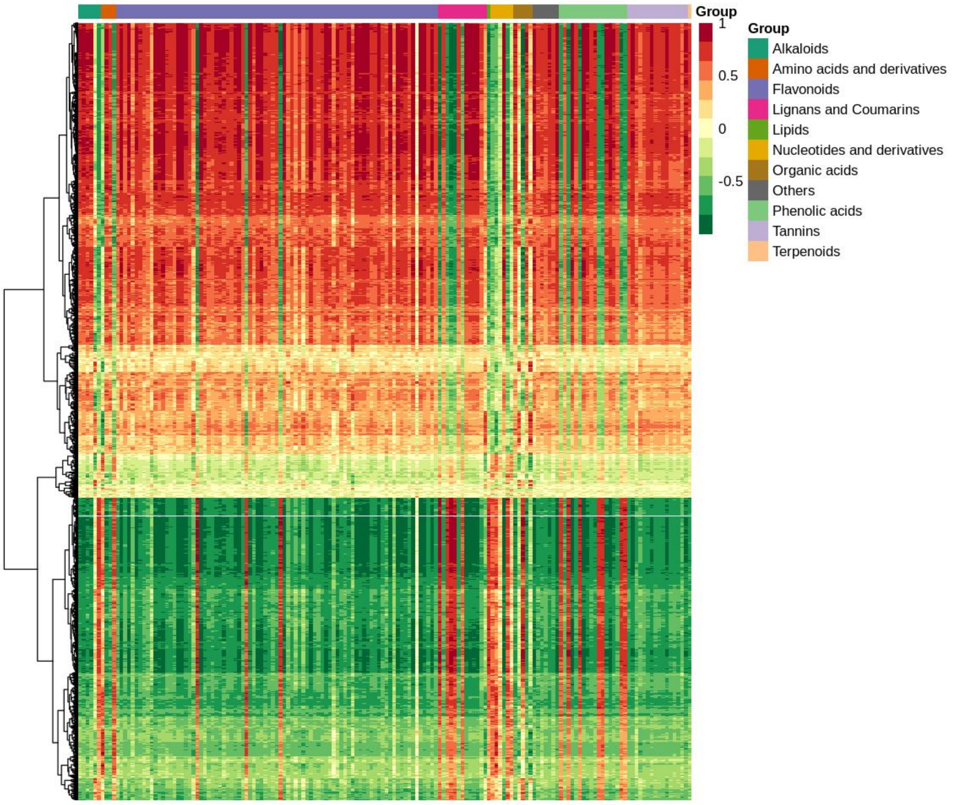

Supplementary Figure 7. KEGG enrichment diagram (A) and the heatmap (B) for the integrated DEGs and DAMs
